# Synthesis, Analysis, Cholinesterase-Inhibiting Activity and Molecular Modelling Studies of 3-(Dialkylamino)-2-hydroxypropyl 4-[(Alkoxy-carbonyl)amino]benzoates and Their Quaternary Ammonium Salts

**DOI:** 10.3390/molecules22122048

**Published:** 2017-11-23

**Authors:** Tereza Padrtova, Pavlina Marvanova, Klara Odehnalova, Renata Kubinova, Oscar Parravicini, Adriana Garro, Ricardo D. Enriz, Otakar Humpa, Michal Oravec, Petr Mokry

**Affiliations:** 1 Department of Chemical Drugs, Faculty of Pharmacy, University of Veterinary and Pharmaceutical Sciences, Palackeho 1, 61242 Brno, Czech Republic; padrtova.tereza@seznam.cz (T.P.); marvanovap@gmail.com (P.M.); odehnalovak@vfu.cz (K.O.); 2 Department of Natural Drugs, Faculty of Pharmacy, University of Veterinary and Pharmaceutical Sciences, Palackeho 1, 61242 Brno, Czech Republic; kubinovar@vfu.cz; 3 Facultad de Química, Bioquímica y Farmacia, Universidad Nacional de San Luis-IMIBIO-SL-CONICET, Chacabuco 915, San Luis 5700, Argentina; oparravicini@gmail.com (O.P.); adrianagarrosl@gmail.com (A.G.); danielenriz@gmail.com (R.D.E.); 4 CEITEC–Central European Institute of Technology, Masaryk University, Kamenice 753/5, 62500 Brno, Czech Republic; humpa@chemi.muni.cz; 5 Global Change Research Institute CAS, Belidla 986/4a, 60300 Brno, Czech Republic; oravec.m@czechglobe.cz

**Keywords:** arylcarbonyloxyaminopropanols, tertiary amines, quaternary ammonium salts, acetylcholinesterase, butyrylcholinesterase

## Abstract

Tertiary amines 3-(dialkylamino)-2-hydroxypropyl 4-[(alkoxycarbonyl)amino]benzoates and their quaternary ammonium salts were synthesized. The final step of synthesis of quaternary ammonium salts was carried out by microwave-assisted synthesis. Software-calculated data provided the background needed to compare fifteen new resulting compounds by their physicochemical properties. The acid dissociation constant (p*K*_a_) and lipophilicity index (log *P*) of tertiary amines were determined; while quaternary ammonium salts were characterized by software-calculated lipophilicity index and surface tension. Biological evaluation aimed at testing acetylcholinesterase and butyrylcholinesterase-inhibiting activity of synthesized compounds. A possible mechanism of action of these compounds was determined by molecular modelling study using combined techniques of docking; molecular dynamics simulations and quantum mechanics calculations.

## 1. Introduction

According to the latest World Health Organization (WHO) reports, around 47 million of people worldwide suffer from dementia, the most common cause of neurological disorder of the central nervous system—Alzheimer’s disease (AD). In addition, there are 9.9 million new cases every year. By 2050, more than 100 million individuals will have suffered from AD as estimated [1,2], where death usually occurs 5–10 years after a clinically diagnosed [3]. Approved AD-treatments (galantamine, donepezil, rivastigmine, tacrine and memantine) demonstrate symptomatic effects, but do not stem or at least slow disease progress [4,5]. Therefore, the development of new drugs, influencing parasympathomimetic nervous system, is still of the utmost importance. AD results in loss of neuronal functionality, causes synaptic damage and induces cholinergic neurotransmission dysfunction. This occurs due to reduced acetylcholine (ACh) levels in synapses as well as decreasing number of nicotinic and muscarinic receptors, increase in oxidative stress and massive neuronal loss [6]. Acetylcholinesterase (AChE) is a serine hydrolase responsible for deacetylation of ACh that causes termination of impulse transmission at cholinergic synapses in both central and peripheral nervous system [7].

Early kinetic studies indicated that the active site of AChE contains two subsites, the ‘esteratic’ and ‘anionic’ subsite, corresponding respectively to the catalytic machinery and the choline-binding pocket. The ‘esteratic’ subsite is likely to resemble the catalytic subsites of other serine hydrolases. The ‘anionic’ subsite interacts with the charged quaternary group of choline moiety of ACh and is believed to be the binding site for quaternary ligands, such as edrophonium and *N*-methylacridinium, which act as competitive inhibitors [7].

Quaternary ammonium salts (QUATs) are compounds with wide use in organic synthesis, pharmacy or other fields of industry. Thanks to their specific physical and chemical properties, due to their amphiphilic character, they are already used as phase-transfer catalysts, ionic liquids, dyes, antimicrobial agents and disinfectants, antiarrhythmics, bronchodilators, AChE inhibitors, reactivators of inhibited AChE etc., and there are still efforts to prepare new active compounds [8].

The main goal of our research was synthesizing various series of compounds with the purpose of evaluating the inhibiting activity against AChE. Due to presence of carbamate moiety and ammonium group (NR_4_^+^) in their structure, these compounds can be considered as AChE inhibitors. Physicochemical properties and structure of the compounds put together important information which will later help us determine “chemical structure-biological effect” relationship. Furthermore, in order to determine a possible mechanism of action of the compounds reported here, we performed a molecular modelling study using combined techniques (docking, MD simulations and QTAIM calculations). In this study, a well-known AChE inhibitor, rivastigmine, was included in the analysis. Thus, the possible stereo-electronic requirements for these new ligands were also discussed regarding to their different affinities.

## 2. Results

### 2.1. Chemistry

**Scheme 1:**
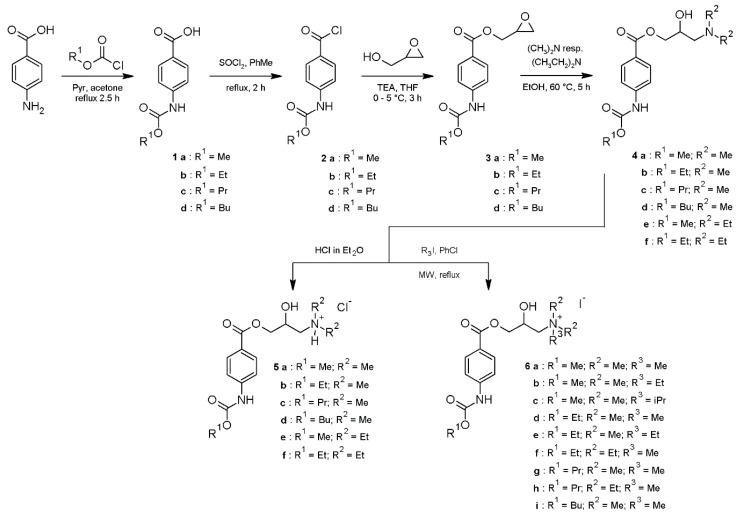
Synthesis of compounds **1**–**6**.

The synthesis (Scheme 1.) started with the reaction between 4-aminobenzoic acid and alkyl chloroformates in acetone giving the appropriate 4-[(alkyloxycarbonyl)amino]benzoic acids **1a**–**d**. The following alkyl [4-(chlorocarbonyl)phenyl]carbamate intermediates **2a**–**d** were prepared via reaction of acids **1a**–**d** from the previous step with the thionyl chloride in dry toluene. These unstable acyl chlorides were immediately subjected to the next reaction when alkyl[4-(chlorocarbonyl)phenyl]carbamates **2a**–**d** was added dropwise to the mixture of (oxiran-2-yl)methanol and triethylamine in tetrahydrofuran giving the appropriate (oxiran-2-yl)methyl 4-[(alkyloxycarbonyl)amino]benzoates **3a**–**d**. This reaction was carried out at 0–5 °C, resp. ambient temperature to not open the oxirane ring prematurely [9]. The oxirane ring was later opened by the addition of secondary amines (*N*,*N*-dimethyl- or *N*,*N*-diethylamine, respectively) to form the appropriate 3-(dialkylamino)-2-hydroxypropyl 4-[(alkoxycarbonyl)amino]benzoate tertiary amines **4a**–**f** [10]. These bases were converted to hydrochloride salts **5a**–**f** to enhance their solubility in water. To synthesize final QUATs **6a**–**i**, it was necessary to convert the hydrochloride salts back to their bases with NaHCO_3_. Subsequently, the final QUATs were prepared via Menshutkin reaction between tertiary amines and alkylhalogenides [11]. First, this final step was accomplished using conventional laboratory synthesis. To prepare the compound **6c**, the appropriate tertiary amine reacted with 2-iodopropane in butan-2-one. However, due to the long reaction time and low yield, the decision was taken to use a more up-to-date way—microwave synthesis. Also, the solvent was changed from butan-2-one to chlorobenzene. The yield increased (from 22% to 70%) and the reaction time was significantly reduced (from 10 h to 1 h). From this point on, all QUATs have been synthesized this way. Finally, the reactions of tertiary amines **5a**–**f** and alkylating agents were performed in a microwave reactor using dry chlorobenzene as a solvent. The reaction time depended on the alkylation agent while alkylating ability rises in the order: isopropyl iodide < ethyl iodide < methyl iodide (see Table 1).

### 2.2. Acetylcholinesterase (AChE) and Butyrylcholinesterase (BuChE) Inhibiting Activity

A slightly modified method of measuring AChE and BuChE inhibitory activity, originally developed by Ellman [12], was performed in a 96-well microplate reader. Thus, 170 μL of 0.1 M phosphate buffer (pH 7.0), 20 μL of AChE (electric eel) or BuChE (equine serum) in phosphate solution (2.3 U/mL), 20 μL of tested compound in methanol and 20 μL of 10 mM (5,5′-dithiobis(2-nitrobenzoic acid, DTNB, Ellman’s Reagent)) were being mixed and incubated at 37 °C for 15 min. Then, 20 μL of 7.5 mM acetylthiocholine iodide (ATCI)/butyrylthiocholine iodide (BTCI) were added. The absorbance was measured at 405 nm (Abs_sample_). Each measurement was repeated five times. One set of mixtures prepared with equal volume of methanol instead of tested samples was used as a control (Abs_control_). As a first step, all tertiary amines and QUATs were tested for their percentage ability to inhibit AChE and BuChE at concentration 100 μM. The inhibitory rates IR (%) were calculated according to the Equation (1). After that, the compounds that exhibited inhibitory potency higher than 50% at concentration 100 μM, were selected for determination of IC_50_ values. These values were calculated by a non-linear regression of the log(concentration)-response curve using SigmaPlot software (v. 11.0; Systat Software GmbH, Erkrath, Germany):

(1) $$IR=\left[1-\left(Abs_{sample}/Abs_{control}\right)\right]\;\times \;100$$

According to the results in Table 2, the QUATs **6a**–**i** showed a stronger tendency to inhibit AChE than tertiary amines **5a**–**f**. Compounds with the most promising IC_50_ were **6f**,**h**, their common feature is a *N*,*N,N*-diethylmethylamine moiety in the basic part of the chemical structure. Generally, compounds with more bulky substituents in the basic part of the molecule had stronger ability to inhibit both enzymes. When it comes to the inhibition of BuChE, the longer alkyl chain in carbamate moiety (propyl-, resp. butyl-) was a common feature of compound **6h**,**i**, the most active BuChE inhibitors. If we compare the results of the tested compounds with rivastigmine, it is clear that they possess ten times lower inhibition activity against AChE than rivastigmine, which is in accordance to a molecular modelling study that was also performed, see Section 2.3. Rivastigmine is highly selective for BuChE, whereas synthetized compounds demonstrated greater potency for AChE over BuChE.

### 2.3. Molecular Modelling

To better understand the experimental results described in the previous sections, we performed a molecular modelling study for the different compounds reported here. This study was conducted in three different stages. In the first stage, we performed a docking analysis using Autodock program [14]. In the second stage of this study, we carried out molecular dynamics (MD) simulations using AMBER software package [15]. Using the trajectories obtained from MD simulations, we performed an analysis per residue for the respective compounds. Finally, to better appreciate the molecular interactions involved in the different L-R complexes, we performed a Quantum Theory of Atoms in Molecules (QTAIM) [16] study for the most active structures of this series. In this study, a well-known AChE inhibitor, rivastigmine, was also included.

It should be noted that compounds reported here possess one chiral centre and therefore they are enantiomeric with the possibility of existence in two isomers (*S* and *R*). However, we didn’t perform an enantiomeric resolution for the previously mentioned biological assays; thus, in order to consider such situation, both isomers of each compound were evaluated in the simulations.

**Figure 1:**
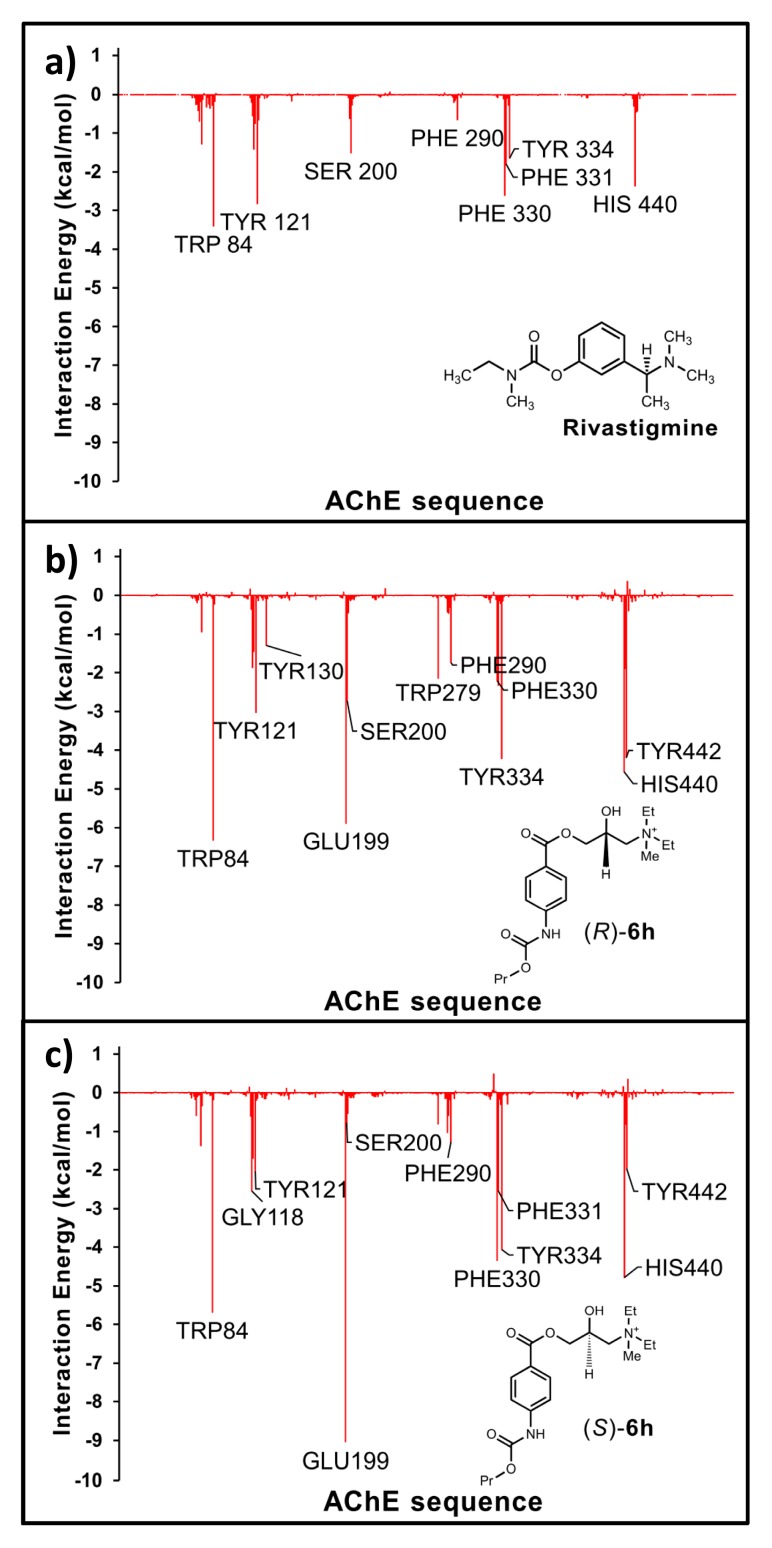
Histograms of interaction energies partitioned with respect to the AChE, amino acids in complex with (**a**) rivastigmine; (**b**) (*R*)-**6h** and (**c**) (*S*)-**6h**. The *X*-axis denotes the residue number of AChE, and the *Y*-axis denotes the interaction energy between the compounds and specific residue. Negative values and positive values are favorable or unfavorable to binding, respectively.

Our molecular dynamics simulations suggest that the compounds reported here show interactions closely related to those of rivastigmine (see Figure 1). In this figure, interactions obtained for compound **6h** in its two enantiomeric forms are shown. Due to the fact that the results obtained for both isomers are quite similar, it would be reasonable to expect similar activities for both of them. The only difference observed for these isomers is that for the (*S*) form the interaction with Glu199 is stronger than that obtained for the (*R*)-isomer; related to this, the interaction obtained for Ser200 in the S enantiomer is significantly weaker than that observed for (*R*)-form. For reasons of space in the text, we only here discuss the results obtained for compound **6h**, but similar results were obtained also for the rest of the compounds of this series. Some of them are shown as Supplementary Material (Figure S1).

The main interactions stabilizing the different complexes are: Trp84, Tyr121, Glu199, Ser200, Phe330, Tyr334 and His440, among others. Considering the similar interaction profile obtained for these compounds compared to that obtained for rivastigmine, it is reasonable to assume that these compounds are interacting in the same active site of the enzyme (compare the histograms shown in Figure 1a–c). However, there is a significant difference in the behaviour of rivastigmine and the QUATs. Note that QUATs show a strong interaction with Glu199, however this interaction is not present in rivastigmine. This is an important difference that could explain why these compounds possess a significantly lower activity in comparison to rivastigmine. In order to evaluate these interaction differences more precisely and in detail, we performed a QTAIM study for the different complexes.

Rivastigmine is a well-known pseudo-irreversible inhibitor of AChE [17]. It should be noted that the carbamates reported in this work have a marked structural resemblance to rivastigmine, at least much higher than with galantamine-type alkaloids. Therefore, the comparative study has been carried out with rivastigmine. The experimental results clearly show that these carbamates are significantly weaker inhibitors than rivastigmine. By comparing the structures, it is clear that the amines are poor leaving-groups compared to the phenol group of rivastigmine. This could explain, at least partially, the lower inhibitory activity of these carbamates in comparison with rivastigmine. The compounds reported here might be considered as starting structures, structural modifications in this portion of the molecule would be important to increase their inhibitory capacity.

#### Evaluating the Molecular Interactions for the Different Complexes

The topological analysis of the electron density allows a deep examination of the molecular interactions. QTAIM analysis offers an unequivocal way to determine strong and weak interactions between two atoms observing the existence of Bond Critical Points (BCPs) and their respective bond paths. We successfully used this type of analysis in different biological sites [18,19,20,21,22].

*X*-ray crystallography data fully support the kinetic findings indicating that rivastigmine reacts stoichiometrically (1:1) with Ser200 in the active site of AChE to form a stable ethylmethylcarbamylated enzyme [23]. Such results support the model of Wilson [24,25], which treated carbamates as slow substrates of AChE. Interaction with Ser200 plays key role in mechanism of how rivastigmine reacts, as these results clearly suggest.

**Figure 2:**
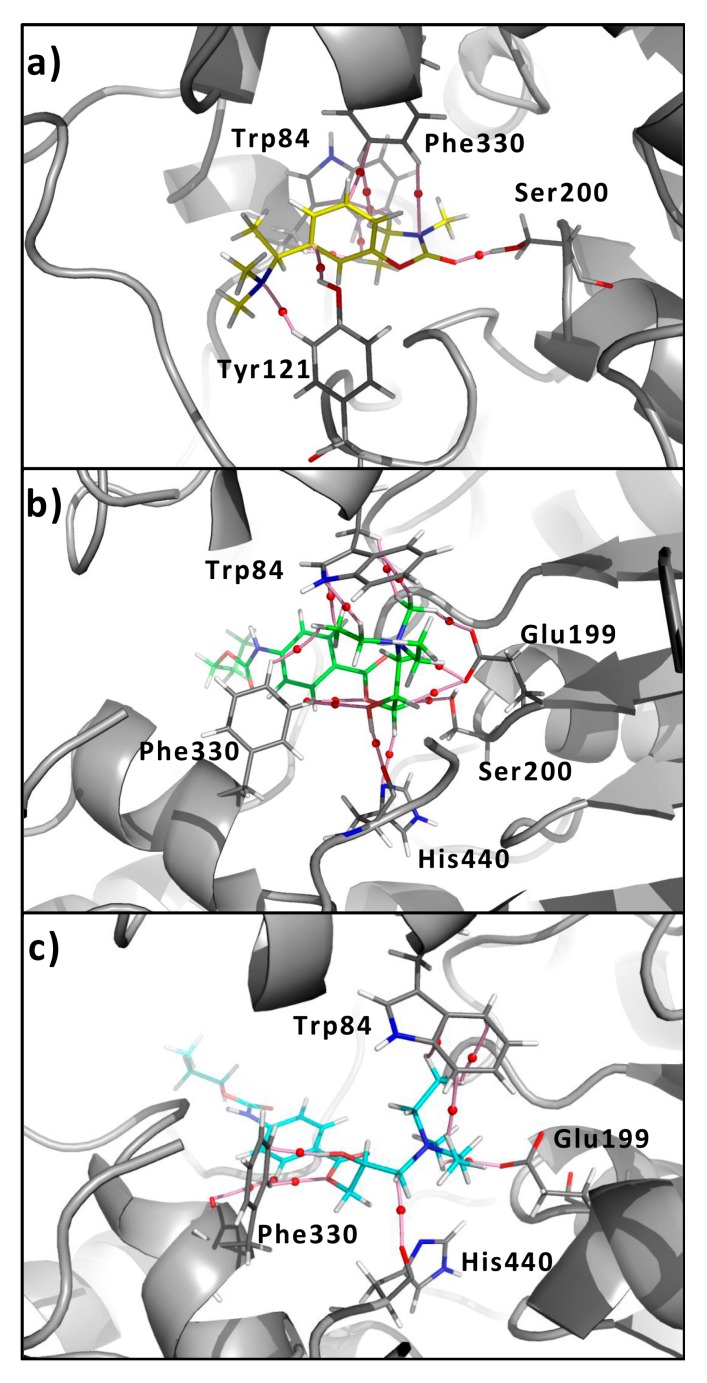
Molecular graph of the non-covalent interactions between the main residues of AChE with (**a**) rivastigmine (yellow sticks); (**b**) compound (*R*)-**6h** (green sticks); and (**c**) (*S*)-**6h** (light-blue sticks). The elements of the electron density topology are shown. The bond paths connecting the nuclei are represented in pink sticks and the bond critical points are shown as red spheres.

Figure 2a shows the major molecular interactions responsible for stabilizing the rivastigmine/AChE complex. In this figure, strong interactions can be observed with Ser200, Trp84, Tyr121 and Phe330. Following Figure 2b,c portray the interactions obtained for the complexes of compound **6h** with AChE in its (*R*)- and (*S*)-form, respectively. It is important to note that while compound **6h** interacts at the same site as rivastigmine, due to its different spatial arrangement, it shows different interactions. Being the most noticeable difference, the interaction with Ser200 is missing for the (*S*)-isomer; whereas in the case of the (*R*)-isomer there is a very weak interaction with Ser200. This interaction is very weak (only 0.01), compared to the interaction of the carbamate group of rivastigmine, which displays a roh value of 0.04. Very similar results were obtained for the rest of the active compounds of this series.

QTAIM analysis results are in full compliance with previous experiments. Thus, Figure 2a shows a strong interaction between the carbamate group of rivastigmine and the OH group of Ser200. In contrast, compound **6h** fails to make this interaction. Because the spatial ordering of these compounds at the active site is different to that of rivastigmine, it doesn’t allow interaction with Ser200. This is an important difference observed between the QUATs reported here and rivastigmine. This different behaviour could explain, at least in part, the lower inhibitory activity obtained for these compounds when compared to that of rivastigmine. Since these QUATs do not possess the same pharmacophoric pattern as rivastigmine, it would be necessary to introduce structural changes into these compounds in order to increase their inhibiting effect on AChE.

### 2.4. Physicochemical Properties Determination

Physicochemical properties as lipophilicity (log *P*), distribution coefficient (log *D*) and acidobasic dissociation constant (p*K*_a_) can provide a base to estimate pharmacokinetic parameters (ADME). The dissociation constant (p*K*_a_) describes a situation when acid/base character sets the charge of a molecule in solution at a particular pH. Charge state of the molecule is necessary for understanding the absorption, transport and receptor binding of drugs at the molecular level. Lipophilicity is another molecular feature of immense importance in medicinal chemistry. Based on their value we could predict possibility of passive transport of drugs through the lipoid membranes in the human body. Although logarithm of octanol/water partition coefficient (log *P_oct_*) is the most extensively used parameter to quantify lipophilicity, nowadays extrapolated log *k_w_* values is a popular alternative of lipophilicity assessment which correlate well with log *P_oct_* values [26,27,28].

#### 2.4.1. Determination of Lipophilicity and Acidobasic Constant of Tertiary Amines

HPLC provides a user’s friendly, rapid, and compound sparing methodology, which is successfully applied to determine drug lipophilicity. The lipophilicity determination is based on monitoring of the logarithmic retention factor (log *k*) of the analyte with changing ratio of organic mobile phase fraction. Under suitable chromatographic conditions, isocratic and extrapolated retention factors correlate well with octanol-water partition or distribution coefficients [27,28]. The lipophilicity index measured by HPLC is derived from the retention time *t_r_* that is converted to the logarithm of the retention factor log *k* according to the Equation (2):

(2) $$\log k=\log\left(\frac{t_{r}-t_{0}}{t_{0}}\right)$$

Equation (2): *t_r_*—retention time of analyte, *t*_0_—retention time of an unretained solute (KI). Isocratic retention factors represent a relative scale of lipophilicity. However, extrapolated retention factors log *k_w_*, corresponding to pure water as mobile phase, are considered to be more representative lipophilicity indices. Extrapolated log *k_w_* values are derived using the linear part of the log *k*/Ф relationships, where Ф is the concentration of the organic modifier in the mobile phase [27,28]. The substances were analysed at pH value of 9.0, when they are partially ionized. It is important to note that at lower pH (e.g., physiological pH = 7.4) poor retention on the column (in a given range of Ф, which shouldn’t decrease under 0.2) was observed [27]. The retention time (*t_r_*) was close to the retention time of an unretained solute (*t*_0_).

The p*K_a_* values were determined from monitoring the dependence of effective mobility of the analyte, measured by means of capillary electrophoresis (CE), on pH of the background electrolyte (BGE) using non-linear regression analysis. Monitoring of the effective mobilities of an ionizable compound were performed in a series of electrolyte solutions of constant ionic strength and various pH values [29,30,31].

All the measured, calculated and predicted lipophilicity value are shown in Table 3. The lipophilicity indexes (log *k_w_*^9.0^) of synthetized tertiary amines ranged from 0.42 to 1.97. The correlation coefficients were greater than 0.987 with covariance equal or less than 0.02 units (*n* = 2). The lipophilicity increases with the prolongation of carbon chain in order from methyl to butylcarbamate (–CH_3_ < –CH_2_CH_3_ < –CH_2_CH_2_CH_3_ < –CH_2_CH_2_CH_2_CH_3_), that is in accordance with similar derivatives already described in the literature [32]. Also prolongation of alkyl substitution of secondary amine nitrogen leads to increase of lipophilicity indexes (–NH^+^(CH**_3_**)_2_ < –NH^+^(CH_2_CH_3_)_2_). All the prediction software share the same trend, in other words the software calculated values correlate with experimental data but the calculated values are higher than the experimental ones. With the increasing lipophilicity inhibiting activity against AChE also increases. On the other hand, experimental data for BuChE inhibition did not show any correlation with lipophilicity.

In Table 3, the dissociation constants of studied compounds are given as well. The correlation coefficients of the experimentally determined p*K*_a_ values were greater than 0.992 and standard deviation equal or less than 0.04 units (*n* = 4). The experimental values are in the range from 8.44 to 9.36, with the highest values recorded for the *N*,*N*-diethoxy compounds **5e**,**f**. The value was slightly decreased with prolongation of the carbon chain in carbamates from methyl to butyl (–CH_3_ > –CH_2_CH_3_ > –CH_2_CH_2_CH_3_ > –CH_2_CH_2_CH_2_CH_3_), with the same value for ethyl and propyl derivatives. The software predictions, on the contrary, did not predict any change in connection with prolongation of the carbon chain of the carbamates. Rivastigmine and galantamine, the clinically used AChE inhibitors, have p*K*_a_ values 8.99 and 8.32 [33], respectively, which are very similar to the studied compounds.

#### 2.4.2. Software Calculated Data of Lipophilicity and Surface Tension Determination of QUATs

Surface activity of quaternary ammonium derivatives was determined using a stalagmometric method based on the correlation between surface tension of a liquid and weight of drops dripping from a stalagmometer. The measurement consisted of weighting 20 drops of reference liquid, which was water in this case, and 20 drops of tested compound (c = 0.001 M, T = 21 °C) [34]. Surface tension σ (N.m^−1^) was calculated according to the following Equation (3):

(3) $$\sigma =\sigma_{H2O}\times \frac{m}{m_{H2O}}$$

where: *σ_H_2*O*__* = 0.07259 N·m^−1^ at 21 °C; *m* = weight of 20 drops of a test substance, *m_H_*_2*O*_ = weight of 20 drops of water. The resulting values (N·m^−1^) indicate the percentage (%) where the substance lowers the surface tension of water at a concentration of 0.001 M.

As you can see in Table 4, the ability to lower surface tension was connected with the extension of carbon chain of carbamates (in case of propyl- and butyl-carbamates) and/or with prolongation of alkyl substitution on the quaternary nitrogen (*N*,*N*-diethyl and *N*-methyl). Compounds **6a**, **b** and **d** with *N*,*N*,*N*-trimethyl or *N*,*N*-dimethyl and *N*-ethyl-substitution on the quaternary nitrogen showed the lowest calculated lipophilicity among the QUATs and were not lowering the surface tension at all. Compound **6c** with average lipophilicity showed five time higher activity than the other tested compounds, that is probably related with having as the only one *N*-isopropyl substitution on the quaternary nitrogen.

## 3. Materials and Methods

### 3.1. General Information

All reagents were procured from Sigma-Aldrich (St. Louis, MO, USA) and Acros Organics (Geel, Belgium) in sufficient purity, solvents from Lach-Ner (Neratovice, Czech Republic) were dried or freshly distilled if necessary. TLC Kieselgel 60 F_254_ plates (Merck, Darmstadt, Germany) visualized by UV irradiation (254 nm) were utilized to monitor reactions and purity of prepared substances and reverse-phase TLC RP-18 F_254_ plates (Merck) for the final compounds. Melting points were determined on Kofler hot-plate apparatus HMK (Franz Kustner Nacht KG, Dresden, Germany) and remain uncorrected.

Monitoring of purity of the resulting compounds ran on Dionex Ultimate 3000 Series HPLC instrument (Thermo Fisher Scientific, Waltham, MA, USA). The Chromeleon^®^ software (version 7.2, Thermo Fisher Scientific, Waltham, MA, USA) was employed to collect the data. The purity of compound was formulated as relative proportion of the peak area of the analyte to the total area of all peaks in the chromatogram after subtracting the peak areas in the solvent chromatogram (blank). The mobile phase consisted of acetonitrile: 25 mM formate buffer (pH = 5.0) in the ratio of 3:7 (*v*/*v*). For the distribution of substances, the YMC-Triart C_18_ column (150 × 2 mm; 3 μm) maintained at 30 °C was used. The flow rate was set up to 0.2 mL min^−1^, and detection of compounds at 273 nm.

The log *k_w_*^9.0^ values of synthetized compounds were measured by a Dionex Ultimate 3000 Series HPLC instrument (Thermo Fisher Scientific) controlled through the Chromeleon^®^ software (version 7.2). The separation was performed on ZORBAX Extend-C_18_ (3 × 150 mm, 3.5 μm) column (Agilent Technologies, Waldbronn, Germany). Mobile phase consists of 0.02 M *N*-[tris(hydroxymethyl)-methyl]-3-aminopropanesulfonic acid with addition of 0.15% of *n*-decylamine (pH = 9.0; constituent A) and methanol to which 0.25% of *n*-octanol was added (constituent B). Total flow rate reached 0.4 mL min^−1^, the injection volume was 1 μL, and the column temperature was maintained at 25 °C. The detection wavelength of 254 nm was set up. One milligram of sample was dissolved in 1 mL of methanol and diluted to concentration 0.1 mg mL^−1^ with 50% methanol. Retention factors of the compound were measured under isocratic conditions in the range 50:50–70:30 (A:B; *v*/*v*), 5 values for each factor in duplicate. Linear regression analysis was performed alongside with log *k_w_*^9.0^ values calculation based on Equation (4):

(4) $$\log\;k=-S\Phi +\log\;k_{w}$$

where log *k* represents a logarithm of an individual isocratic factor, Φ is the organic phase concentration, and *S* is a constant derived from linear regression analysis. Retention factor corresponding with the neutral form (log *k_w_*) was estimated from the apparent log *k_w_*^9.0^ by using Equation (5):

(5) $$\log\;k_{w}=\log\;k_{w}^{app}+\log\left(1+10^{pKa-pH}\right)$$

CE experiments were carried out on Agilent 3D CE (Agilent Technologies, Santa Clara, CA, USA) capillary electrophoresis system equipped with an autosampler, automatic injector, photodiode array detector, and an air cooling unit for the capillary. Instrument control and analysis were performed with 3D-CE ChemStation software (Agilent Technologies). Uncoated fused-silica capillary (Agilent Technologies) of 50 μm internal diameter, the total length of 33.0 cm and effective length (to the detector) of 24.5 cm was used. Capillary cassette temperature was maintained at 25 °C with air cooling. Three milligrams of the sample were dissolved in 1 mL of 50% methanol and 10 times diluted with appropriate background electrolyte (BGE) before analysed. Samples were injected hydrodynamically at 40 mbar pressure for 4.0 s. Operating voltage was 15 kV of positive polarity. UV detection was performed at 254 nm. Mesityl oxide was used as a marker of electroosmotic flow. Effective mobilities were recorded for BGE pH values in the range from 3.5 to 11.1 (ionic strength equal to 10 mM), 4 runs at 14 different buffer pH values. The effective mobilities μ_eff_ were calculated according to Equation (6):

(6) $$\mu_{eff}=\frac{L_{tot}L_{eff}}{U}\left(\frac{1}{t_{mig}}-\frac{1}{t_{EOF}}\right)$$

where *t_mig_* is migration time of the analyte and *t_EOF_* is migration time of a neutral marker compound that is of the same velocity as the electroosmotic flow (EOF). *L_eff_* is distance from the injection end of the capillary to the detector and *L_tot_* is the total length of the capillary over which the voltage *U* was applied. SigmaPlot for Windows version 11.0 (Systat Software GmbH, Erkrath, Germany) was employed for the nonlinear regression analysis of μ_eff_/pH relationship having characteristic sigmoidal shape with inflexion point indicating p*K*_a_ value. The pH measurements were taken with a combination glass electrode (HI 1332B, HANNA Instruments, Woonsocket, RI, USA), using a Thermo Orion 370 PerpHect^®^ pH meter (Thermo Fisher Scientific, Waltham, MA, USA).

High-resolution mass spectra of the final compounds were measured using a Dionex Ultimate 3000 Series high-performance liquid chromatograph (Thermo Fisher Scientific) coupled with a LTQ Orbitrap XL™ Hybrid Ion Trap-Orbitrap Fourier Transform Mass Spectrometer (Thermo Fisher Scientific, Waltham, MA, USA) with injection into HESI II.

The NMR spectra of compounds **5c,d** and **6a**–**i** in their liquid state were measured in DMSO-*d*_6_ solution on a Bruker Avance III HD NMR spectrometer 700 MHz (700.25 MHz for ^1^H and 176.08 MHz for a ^13^C nucleus; Bruker BioSpin GmbH, Rheinstetten, Germany) equipped with 5 mm sensitive triple-resonance (^1^H-^13^C-^15^N) cryoprobe optimized for ^13^C detection. The NMR spectra of all intermediates and final compounds **5a**, **b**, **e** and **f** in the liquid state were measured in DMSO-*d*_6_ on a JNM-ECZ4OOR FT-NMR spectrometer 9.39 T (399.78 MHz for ^1^H and 100.53 MHz for ^13^C nucleus; Jeol Resonance, Tokyo, Japan) equipped with a 5 mm High Sensitivity PulseField Gradient Autotune™ probe. Chemical shifts are reported in ppm, referenced to the chemical shifts of residual solvent resonance (DMSO, 2.5 ppm for ^1^H and 39.5 ppm for ^13^C). The coupling constants (*J*) are reported in Hz. Spectra were recorded at the temperature 30 °C. All major signals were assigned on the basis ^1^H, ^13^C{H}, ^13^C-APT, ^1^H-^1^H COSY, ^1^H-^13^C HSQC, ^1^H-^13^C HMBC and ^1^H-^15^N HMBC experiments.

Infrared (IR) spectra were measured on a Nicolet Nexus FT-IR spectrometer (Thermo Scientific) using ATR (ZnSe) instrumentation. The spectra were obtained by accumulation of 12 scans with 4 cm^−1^ resolution in the region 4000–600 cm^−1^. The instrument was controlled by software Omnic v. 8.3 (Thermo Scientific).

The last reaction (Section 3.2.5) was carried out in monomodal StartSYNTH, Microwave reactor Synthesis Labstation at 2.45 GHz (M/s, Milestone S.r.l., Milan, Italy). The device is equipped with an industrial magnetron and a microwave diffuser is located above microwave chamber with a continuous output of 0–1400 W. Temperatures were measured by infrared sensor.

### 3.2. Synthesis

#### 3.2.1. Synthesis of Carbamate Intermediates

An equivalent of pyridine (0.15 mol) was added to a solution of 4-aminobenzoic acid (0.15 mol) in acetone (200 mL). Subsequently, the appropriate methyl, ethyl, propyl or butyl chloroformate (0.15 mol) was added dropwise and the reaction mixture was heated under reflux to 70 °C for 2.5 h. After evaporation of the solvent, the resulting crystals were washed with distilled water on a glass frit and were recrystallized from 50% ethanol.

*4-[(Methoxycarbonyl)amino]benzoic acid* (**1a**). Yield: 89%; R_f_: 0.54 (ethyl acetate/petroleum ether/MeOH 10:1:1); m.p.: 204–207 °C; ^1^H-NMR (400 MHz, DMSO-*d_6_*) δ (ppm): 12.67 (s, 1H, –COOH); 10.04 (s, 1H, –NH); 7.86 (d, *J* = 8.6 Hz, 2H, –C**H**_Ar(–COOH)_); 7.56 (d, *J* = 8.6 Hz, 2H, –C**H**_Ar(–NHCOO–)_); 3.69 (s, 3H, –CH_3_); ^13^C-NMR (100 MHz, DMSO-*d_6_*) δ (ppm): 166.9 (–COOH); 153.8 (–NHCOO–); 143.4 (–**C**_Ar(–NHCOO–)_); 130.5 (–**C**H_Ar(–COOH)_); 124.4 (–**C**_Ar(–COOH)_); 117.3 (–**C**H_Ar(–NHCOO–)_); 51.8 (–CH_3_).

*4-[(Ethoxycarbonyl)amino]benzoic acid* (**1b**). Yield: 96%; R_f_: 0.69 (ethyl acetate/petroleum ether/MeOH 10:1:1); m.p.: 197–200 °C; ^1^H-NMR (400 MHz, DMSO-*d*_6_) δ (ppm): 12.66 (s, 1H, –COOH); 10.02 (s, 1H, –NH); 7.86 (d, *J* = 8.6 Hz, 2H, –C**H**_Ar(–COOH)_); 7.57 (d, *J* = 8.6 Hz, 2H, –C**H**_Ar(–NHCOO–)_); 4.14 (q, *J* = 7.3 Hz, 2H, –CH_2_–); 1.25 (t, *J* = 7.3, 3H, –CH_3_); ^13^C-NMR (100 MHz, DMSO-*d_6_*) δ (ppm): 166.8 (–COOH); 153.3 (–NHCOO–); 143.3 (–**C**_Ar(–NHCOO–)_); 130.3 (–**C**H_Ar(–COOH)_); 124.2 (–**C**_Ar(–COOH)_); 117.2 (–**C**H_Ar(–NHCOO–)_); 60.4 (–CH_2_-); 14.3 (–CH_3_).

*4-[(Propoxycarbonyl)amino]benzoic acid* (**1c**). Yield: 91%; R_f_: 0.80 (ethyl acetate/petroleum ether/MeOH 10:1:1); m.p.: 190–194 °C; ^1^H-NMR (400 MHz, DMSO-*d_6_*) δ (ppm): 12.66 (s, 1H, –COOH); 10.01 (s, 1H, –NH); 7.86 (d, *J* = 8.7 Hz, 2H, –C**H**_Ar(–COOH)_); 7.57 (d, *J* = 8.7 Hz, 2H, –C**H**_Ar(–NHCOO–)_); 4.05 (t, *J* = 6.6 Hz, 2H, –COOCH_2_–); 1.73–1.55 (m, 2H, –CH_2_C**H_2_**CH_3_); 0.93 (t, *J* = 7.5 Hz, 3H, –CH_3_); ^13^C-NMR (100 MHz, DMSO-*d_6_*) δ (ppm): 166.8 (–COOH); 153.4 (–NHCOO–); 143.3 (–**C**_Ar(–NHCOO–)_); 130.3 (–**C**H_Ar(–COOH)_); 124.2 (–**C**_Ar(–COOH)_); 117.2 (–**C**H_Ar(–NHCOO–)_); 65.9 (–COO**C**H_2_–); 21.7 (–**C**H_2_CH_3_); 10.1(–CH_3_).

*4-[(Butoxycarbonyl)amino]benzoic acid* (**1d**). Yield: 70%; R_f_: 0.83 (ethyl acetate/petroleum ether/MeOH 10:1:1); m.p.: 179–182 °C; ^1^H-NMR (400 MHz, DMSO-d_6_) δ (ppm): 12.66 (s, 1H, –COOH); 10.00 (s, 1H, –NH); 7.86 (d, *J* = 8.6 Hz, 2H, –C**H**_Ar(–COOH)_); 7.57 (d, *J* = 8.6 Hz, 2H, –C**H**_Ar(–NHCOO–)_); 4.10 (t, *J* = 6.8 Hz, 2H, –COOCH_2_–); 1.67–1.54 (m, 2H, –CH_2_C**H_2_**CH_2_CH_3_); 1.46–1.28 (m, 2H, –CH_2_CH_2_C**H_2_**CH_3_); 0.91 (t, *J* = 7.3 Hz, 3H, –CH_3_); ^13^C-NMR (100 MHz, DMSO-*d_6_*) δ (ppm): 166.8 (–COOH); 153.4 (–NHCOO–); 143.3 (–**C**_Ar(–NHCOO–)_); 130.3 (–**C**H_Ar(–COOH)_); 124.2 (–**C**_Ar(–COOH)_); 117.2 (–**C**H_Ar(–NHCOO–)_); 64.1 (–COO**C**H_2_–); 30.4 (–**C**H_2_CH_2_CH_3_); 18.4(–CH_2_**C**H_2_CH_3_); 13.4 (–CH_3_).

#### 3.2.2. Synthesis of Acyl Chloride Intermediates

Alkyl [4-(chlorocarbonyl)phenyl]carbamate **2a**–**d** preparation: thionyl chloride (0.12 mol) was added to suspension of compound **1a**–**d** (0.06 mol) in 150 mL of dry toluene (over P_2_O_5_). The reaction mixture was then being heated under reflux to 120 °C for 2 h. Hot solution was filtered through a frit, maternal liquor was cooled down and the precipitated crystals were filtered on sintered glass, washed with petroleum ether and dried in vacuum. Acyl chloride was used immediately to proceed to the next reaction.

#### 3.2.3. Synthesis of Oxirane Intermediates

(Oxiran-2-yl)methanol (0.03 mol) and THF (100 mL) were added to a three-necked flask equipped with a drying calcium chloride tube, a thermometer and a dropping funnel. The mixture was cooled down to −5 °C and triethylamine (0.03 mol) was added to the mixture. Subsequently, appropriate acyl chloride (**2a**–**d**) (0.03 mol) dissolved in THF (50 mL) was dropwise added to the suspension at a rate allowing to maintain the temperature between −5 °C and 5 °C, after which the mixture was being stirred for 3 h at room temperature. The precipitated triethylammonium chloride was then filtered out of the reaction mixture. The solvent from maternal liquor was evaporated under vacuum, producing the crystals of epoxide.

*Oxiran-2-ylmethyl 4-[(methoxycarbonyl)amino]benzoate* (**3a**). Yield: 82%; R_f_: 0.79 (acetone/petroleum ether 2:3); m.p.: 125–129 °C; ^1^H-NMR (400 MHz, DMSO-*d_6_*) δ (ppm): 10.06 (s, 1H, –NH); 7.91 (d, *J* = 8.8 Hz, 2H, –C**H**_Ar(–COO–)_); 7.60 (d, *J* = 8.8 Hz, 2H, –C**H**_Ar(–NHCOO–)_); 4.61 (dd, *^2^J* = 12.4, ^3^*J* = 2.6 Hz, 1H, ArCOOCH_2_–); 4.09 (dd, *^2^J* = 12.4 Hz, *^3^J* = 6.4 Hz, 1H, ArCOOCH_2_–); 3.69 (s, 3H, –CH_3_); 3.31 (m, 1H, CH-oxiran); 2.83 (m, 1H, CH_2_-oxiran); 2.74 (dd, *^2^J* = 5.0 Hz, *^3^J* = 2.6, 1H, CH_2_-oxiran); ^13^C-NMR (100 MHz, DMSO-*d*_6_), δ (ppm): 165.1 (–COO–); 153.5 (–NHCOO–); 143.9 (–**C**_Ar(–NHCOO–)_); 130.4 (–**C**H_Ar(–COO–)_); 122.8 (–**C**_Ar(–COO–)_); 117.4 (–**C**H_Ar(–NHCOO–)_); 65.0 (ArCOO**C**H_2_–); 51.8 (–CH_3_); 49.0 (CH-oxiran); 43.8 (CH_2_–oxiran).

*Oxiran-2-ylmethyl 4-[(ethoxycarbonyl)amino]benzoate* (**3b**). Yield: 93%; R_f_: 0.86 (acetone/petroleum ether 2:3); m.p.: 129–132 °C; ^1^H-NMR (400 MHz, DMSO-*d_6_*) δ (ppm): 10.05 (s, 1H, –NH); 7.91 (d, *J* = 8.8 Hz, 2H, –C**H**_Ar(–COO–)_); 7.61 (d, *J* = 8.8 Hz, 2H, –C**H**_Ar(–NHCOO–)_); 4.59 (dd, *^2^J* = 12.4 Hz, *^3^J* = 2.6 Hz, 1H, ArCOOCH_2_–); 4.16 (q, *J* = 7.1 Hz, 2H, –NHCOOC**H**_2_–); 4.05 (dd, *^2^J* = 12.4 Hz, *^3^J* = 6.4 Hz, 1H, ArCOOCH_2_–); 3.31 (m, 1H, CH-oxiran); 2.83 (m, 1H, CH_2_–oxiran); 2.72 (dd, *^2^J* = 5.0 Hz, *^3^J* = 2.6 Hz, 1H, CH_2_-oxiran); 1.26 (t, *J* = 7.1 Hz, 3H, –CH_3_); ^13^C-NMR (100 MHz, DMSO-*d*_6_) δ (ppm): 165.1 (–COO–); 153.8 (–NHCOO–); 144.0 (–C_Ar(–NHCOO–)_); 130.4 (–**C**H_Ar(–COO–)_); 122.7 (–**C**_Ar(–COO–)_); 117.4 (–**C**H_Ar(–NHCOO–)_); 65.0 (ArCOO**C**H_2_–); 60.5 (–**C**H_2_CH_3_); 49.0 (CH-oxiran); 43.8 (CH_2_-oxiran); 14.4 (–CH_3_).

*Oxiran-2-ylmethyl 4-[(propoxycarbonyl)amino]benzoate* (**3c**). Yield: 80%; R_f_: 0.87 (acetone/petroleum ether 2:3); m.p.: 110–113 °C; ^1^H-NMR (400 MHz, DMSO-*d_6_*) δ (ppm): 10.05 (s, 1H, –NH); 7.91 (d, *J* = 8.8 Hz, 2H, –C**H**_Ar(–COO–)_); 7.62 (d, *J* = 8.8 Hz, 2H, –C**H**_Ar(–NHCOO–)_); 4.60 (dd, *^2^J* = 12.4 Hz, *^3^J* = 2.7 Hz, 1H, ArCOOCH_2_–); 4.05 (m, 3H, –NHCOOC**H**_2_– and ArCOOC**H**_2_–); 3.31 (m, 1H, CH-oxiran); 2.83 (m, 1H, CH_2_-oxiran); 2.72 (dd, *^2^J* = 5.0 Hz, *^3^J* = 2.6 Hz, 1H, CH_2_-oxiran); 1,65 (m, 2H, –CH_2_C**H**_2_CH_3_); 0.94 (t, *J* = 7.4 Hz, 3H, –CH_3_); ^13^C-NMR (100 MHz, DMSO-*d*_6_) δ (ppm): 165.1 (–COO–); 153.4 (–NHCOO–); 144.0 (–**C**_Ar(–NHCOO–)_); 130.4 (–**C**H_Ar(–COO–)_); 122.7 (–**C**_Ar(–COO–)_); 117.4 (–**C**H_Ar(–NHCOO–)_); 66.0 (–**C**H_2_CH_2_CH_3_); 65.0 (ArCOO**C**H_2_–); 49.0 (CH-oxiran); 43.8 (CH_2_-oxiran); 21.7 (–CH_2_**C**H_2_CH_3_); 10.2 (–CH_3_).

*Oxiran-2-ylmethyl 4-[(butoxycarbonyl)amino]benzoate* (**3d**). Yield: 72%; R_f_: 0.90 (acetone/petroleum ether 2:3); m.p.: 98–101 °C; ^1^H-NMR (400 MHz, DMSO-*d_6_*) δ (ppm): 10.04 (s, 1H, –NH); 7.90 (d, *J* = 8.9 Hz, 2H, –C**H**_Ar(–COO–)_); 7.61 (d, *J* = 8.9 Hz, 2H, –C**H**_Ar(–NHCOO–)_); 4.59 (dd, *^2^J* = 12.4 Hz, *^3^J* = 2.7 Hz, 1H, ArCOOCH_2_–); 4.11 (t, *J* = 6.5 Hz, 2H, –NHCOOC**H**_2_–); 4.05 (dd, *^2^J* = 12.4 Hz, *^3^J* = 6.4 Hz, 1H, ArCOOCH_2_–); 3.31 (m, 1H, CH–oxiran); 2.83 (m, 1H, CH_2_–oxiran); 2.72 (dd, *^2^J* = 5.0 Hz, *^3^J* = 2.6 Hz, 1H, CH_2_-oxiran); 1.62 (m, 2H, –CH_2_C**H**_2_CH_2_CH_3_); 1.38 (m, 2H, –CH_2_CH_2_C**H**_2_CH_3_); 0.92 (t, *J* = 7.2 Hz, 3H, –CH_3_); ^13^C-NMR (100 MHz, DMSO-*d_6_*) δ (ppm): 165.1 (–COO–); 153.4 (–NHCOO–); 144.0 (–**C**_Ar(–NHCOO__–)_); 130.4 (–**C**H_Ar(–COO–)_); 122.8 (–**C**_Ar(–COO–)_); 117.3 (–**C**H_Ar(–NHCOO–)_); 65.0 (ArCOO**C**H_2_–); 64.2 (–**C**H_2_CH_2_CH_2_CH_3_); 49.0 (CH-oxiran); 43.8 (CH_2_–oxiran); 30.4 (–CH_2_**C**H_2_CH_2_CH_3_); 18.5 (–CH_2_CH_2_**C**H_2_CH_3_); 13.5 (–CH_3_).

#### 3.2.4. Synthesis of Tertiary Amines as Final Compounds

Compound **3a**–**d** (0.02 mol) in 100 mL of ethanol were added to a round bottom flask. The mixture was carefully heated to 70 °C until the oxirane-compound dissolved. Then, the mixture was cooled down to room temperature and secondary amine (*N*,*N*-dimethyl- or *N*,*N*-diethylamine respectively, 0.02 mol) was added. The reaction mixture was heated to 60 °C and allowed to stir for 5 h. Then, the solvent was evaporated under reduced pressure producing a residue which was suspended in diethyl ether (30 mL). The resulting crystalline impurities were filtered off and ethereal HCl (30 mL) was added to the filtrate producing crystals that were filtered out and recrystallized from acetone: methanol 3:1. The final products were obtained in the form of hydrochloride salt. See the ^1^H- and ^13^C-NMR spectra of compounds **5a**–**f** as Supplementary Materials (Figures S2–S13).

*(2-Hydroxy-3-{4-[(methoxycarbonyl)amino]benzoyloxy}propyl)dimethylammonium chloride* (**5a**). Yield: 75%; R_f_: 0.46 (ethyl acetate/diethylamine 10:1); R_f_ (rev.): 0.75 (0.1 M HCl/acetone 3:2); m.p.: 199–202 °C; HPLC pur. 95.22% (254 nm); IR (Zn/Se ATR): ν (cm^−1^) = 3330 w; 3257 br; 2949 w; 2668 w; 1740 s; 1689 s; 1600 s; 1541 s; 1222 s; 1185 s; ^1^H-NMR (400 MHz, DMSO-*d*_6_): δ (ppm) = 10.15 (s, 1H, –NHCOO–); 9.97 (bs, 1H, –NH^+^); 7.97 (d, *J* = 8.7 Hz, 2H, –C**H**_Ar(–COO–)_); 7.62 (d, *J* = 8.7 Hz, 2H, –C**H**_Ar(–NHCOO–)_); 6.04 (d, *J* = 5.0 Hz, 1H, –OH); 4.30 (m, 1H, –COOCH_2_C**H**–); 4.20 (m, 2H, –COOC**H_2_**CH–); 3.69 (s, 3H, –CH_3_); 3.23 (m, 2H, –CH_2_NH^+^–); 2.82 (s, 6H, –NH^+^(C**H_3_**)_2_); ^13^C-NMR (100 MHz, DMSO-*d*_6_): δ (ppm) = 165.1 (–COO–); 153.8 (–NHCOO–); 144.0 (–**C**_Ar(–NHCOO–)_); 130.7 (–**C**H_Ar(–COO–)_); 122.9 (–**C**_Ar(–COO–)_); 117.3 (–**C**H_Ar(–NHCOO–)_); 66.0 (–COO**C**H**_2_**CH–); 63.4 (–COOCH_2_**C**H–); 58.8 (–CH_2_NH^+^–); 52.0 (–CH_3_); 43.7 and 41.9 (–NH^+^(CH_3_)_2_); HR-MS: C_14_H_19_N_2_O_5_ [M − H]^−^ calculated 295.1299 *m*/*z*; found 295.1304 *m*/*z*.

*(3-{4-[(ethoxycarbonyl)amino]benzoyloxy}-2-hydroxypropyl)dimethylammonium chloride* (**5b**). Yield: 71%; R_f_: 0.54 (ethyl acetate/diethylamine 10:1); R_f_ (rev.): 0.68 (0.1 M HCl/acetone 3:2); m.p.: 168–171 °C; HPLC pur. 96.42% (254 nm); IR (Zn/Se ATR): ν (cm^−1^) = 3346 w; 3261 br; 2987 w; 2671 s; 1737 s; 1693 s; 1597 s; 1539 s; 1216 s; 1182 s; ^1^H-NMR (400 MHz, DMSO-*d_6_*): δ (ppm) = 10.08–10.06 (overlap m, 2H, –NHCOO– + –NH^+^); 7.96 (d, *J* = 8.7 Hz, 2H, –C**H**_Ar(–COO–)_); 7.62 (d, 2H, *J* = 8.7 Hz, –C**H**_Ar(–NHCOO–)_); 6.03 (d, *J* = 5.0 Hz, 1H, –OH); 4.31 (m, 1H, –COOCH_2_C**H**–); 4.20 (m, 2H, –COOC**H_2_**CH–); 4.15 (q, *J* = 7.1 Hz, 2H, –C**H**_2_CH_3_); 3.23 (m, 2H, –CH_2_NH^+^–); 2.83 (s, 6H, –NH^+^(C**H_3_**)_2_); 1.25 (t, *J* = 7.1 Hz, 3H, –CH_2_C**H**_3_); ^13^C-NMR (100 MHz, DMSO-*d_6_*): δ (ppm) = 165.1 (–COO–); 153.3 (–NHCOO–); 144.0 (–**C**_Ar(–NHCOO–)_); 130.6 (–**C**H_Ar(–COO–)_); 122.8 (–**C**_Ar(–COO–)_); 117.3 (–**C**H_Ar(–NHCOO–)_); 66.0 (–COO**C**H**_2_**CH–); 63.4 (–COOCH_2_**C**H–); 60.5 (–**C**H_2_CH_3_); 58.8 (–CH_2_NH^+^–); 43.8 and 41.9 (–NH^+^(CH_3_)_2_); 14.4 (–CH_3_); HR-MS: C_15_H_21_N_2_O_5_ [M − H]^−^ calculated 309.1456 *m*/*z*; found 309.1459 *m*/*z*.

*(2-Hydroxy-3-{4-[(propoxycarbonyl)amino]benzoyloxy}propyl)dimethylammonium chloride* (**5c**). Yield: 60%; R_f_: 0.59 (ethyl acetate/diethylamine 10:1); R_f_ (rev.): 0.61 (0.1 M HCl/acetone 3:2); m.p.: 151–154 °C; HPLC pur. 95.15% (254 nm); IR (Zn/Se ATR): ν (cm^−1^) = 3356 w; 2972 w; 2672 w; 1745 s; 1699 s; 1592 s; 1537 s; 1213 s; 1177 s; ^1^H-NMR (700 MHz, DMSO-*d_6_*): δ (ppm) = 10.17 (bs, 1H, NH^+^); 10.09 (bs, 1H, –NHCOO–); 7.96 (d, *J* = 8.9 Hz, 2H, –C**H**_Ar(–COO–)_); 7.62 (d, *J* = 8.9 Hz, 2H, –C**H**_Ar(–NHCOO–)_); 6.04 (d, *J* = 5.3 Hz, 1H, –OH); 4.32 (m, 1H, –COOCH_2_C**H**–); 4.20 (m, 2H, –COOC**H_2_**CH–); 4.06 (t, *J* = 6.7 Hz, 2H, –C**H**_2_CH_2_CH_3_); 3.24 (m, 2H, –CH_2_NH^+^–); 2.83 (s, 6H, –NH^+^(C**H_3_**)_2_); 1.64 (m, 2H, –CH_2_C**H_2_**CH_3_); 0.93 (t, *J* = 7.4 Hz, 3H, –CH_2_CH**_2_**C**H_3_**); ^13^C-NMR (176 MHz, DMSO-*d_6_*): δ (ppm) = 165.10 (–COO–); 153.42 (–NHCOO–); 144.06 (–**C**_Ar(–NHCOO–)_); 130.67 (–**C**H_Ar(–COO–)_); 122.82 (–**C**_Ar(–COO–)_); 117.30 (–**C**H_Ar(–NHCOO–)_); 66.08 (–COO**C**H**_2_**CH–) and 66.04 (–**C**H_2_**C**H_2_CH_3_); 63.43 (–COOCH_2_**C**H–); 58.83 (–CH_2_N^+^–); 43.76 and 42.01 (–NH^+^(CH_3_)_2_); 21.82 (–CH_2_**C**H_2_CH_3_); 10.24 (–CH_3_); ^15^N (DMSO-*d_6_*): δ (ppm) = 33.5 (–NH^+^(CH_3_)_2_); 103.9 (–NHCOO–); HR-MS: C_16_H_23_N_2_O_5_ [M − H]^−^ calculated 323.1612 *m*/*z*; found 323.1614 *m*/*z.*

*(3-{4-[(Butoxycarbonyl)amino]benzoyloxy}-2-hydroxypropyl)dimethylammonium chloride* (**5d**). Yield: 58%; R_f_: 0.64 (ethyl acetate/diethylamine 10:1); R_f_ (rev.): 0.51 (0.1 M HCl/acetone 3:2); m.p.: 119–122 °C; HPLC pur. 94.73% (254 nm); IR (Zn/Se ATR): ν (cm^−1^) = 3350 w; 2966 w; 2669 w; 1748 s; 1696 s; 1590 s; 1538 s; 1222 s; 1178 s; ^1^H-NMR (700 MHz, DMSO-*d_6_*): δ (ppm) = 10.25 (bs, 1H, NH^+^); 10.11 (s, 1H, –NHCOO–); 7.96 (d, *J* = 8.9 Hz, 2H, –C**H**_Ar(–COO–)_); 7.62 (m, *J* = 8.9 Hz, 2H, –C**H**_Ar(–NHCOO–)_); 6.08 (d, *J* = 5.0 Hz, 1H, –OH), 4.32 (m, 1H, –COOCH_2_C**H**–); 4.20 (m, 2H, –COOC**H_2_**CH–); 4.10 (t, *J* = 6.6 Hz, 2H, –C**H_2_**CH_2_CH_2_CH_3_); 3.24 (m, 2H, –C**H_2_**N^+^–); 2.82 (s, 6H, –NH^+^(C**H_3_**)_2_); 1.60 (m, 2H, –CH_2_C**H_2_**CH_2_CH_3_), 1.37 (m, 2H, –CH_2_CH_2_C**H_2_**CH_3_), 0.91 (t, *J* = 7.3 Hz, 3H, –CH_2_CH_2_CH_2_C**H_3_**); ^13^C-NMR (176 MHz, DMSO-*d*_6_): δ (ppm) = 165.11 (–COO–); 153.40 (–NHCOO–); 144.00 (–**C**_Ar(–NHCOO–)_); 130.66 (–**C**H_Ar(–COO–)_); 122.82 (–**C**_Ar(–COO–)_); 117.29 (–**C**H_Ar(–NHCOO–)_); 66.08 (–COO**C**H**_2_**CH–); 64.23 (–**C**H_2_**C**H_2_CH_2_CH_3_); 63.44 (–COOCH_2_**C**H–); 58.84 (–CH_2_N^+^–); 43.72 and 42.09 (–NH^+^(CH_3_)_2_); 30.48 (–CH_2_**C**H_2_CH_2_CH_3_); 18.58 (–CH_2_CH_2_**C**H_2_CH_3_); 13.59 (–CH_3_); HR-MS: C_17_H_25_N_2_O_5_ [M − H]^−^ calculated 337.1769 *m*/*z*; found 337.1770 *m*/*z.*

*Diethyl-(2-hydroxy-3-{4-[(methoxycarbonyl)amino]benzoyloxy}propyl)ammonium chloride* (**5e**). Yield: 78%; R_f_: 0.71 (ethyl acetate/diethylamine 10:1); R_f_ (rev.): 0.67 (0.1 M HCl/acetone 3:2); m.p.: 165–168 °C; HPLC pur. 88.42% (254 nm); IR (Zn/Se ATR): ν (cm^−1^) = 3310 w; 2957 w; 2603 w; 1728 s; 1686 s; 1598 s; 1539 s; 1229 s; 1182 s; ^1^H-NMR (400 MHz, DMSO-*d_6_*): δ (ppm) = 10.11 (s, 1H, –NHCOO–); 9.80 (bs, 1H, NH^+^); 7.96 (d, *J* = 8.8 Hz, 2H, –C**H**_Ar(–COO–)_); 7.62 (d, *J* = 8.8 Hz, 2H, –C**H**_Ar(–NHCOO–)_); 5.99 (d, *J* = 5.2 Hz, 1H, –OH), 4.33 (m, 1H, –COOCH_2_C**H**–); 4.22 (m, 2H, –COOC**H_2_**CH–); 3.69 (s, 1H, –CH_3_); 3.25–3.09 (overlap m, 6H, –(C**H_2_**)NH^+^(C**H_2_**CH_3_)_2_); 1.24 (t, *J* = 7.2 Hz, 6H, –NH^+^(CH**_2_**C**H_3_**)_2_); ^13^C-NMR (100 MHz, DMSO-*d*_6_): δ (ppm) = 165.1 (–COO–); 153.8 (–NHCOO–); 143.9 (–**C**_Ar(–NHCOO–)_); 130.5 (–**C**H_Ar(–COO–)_); 122.9 (–**C**_Ar(–COO–)_); 117.3 (–**C**H_Ar(–NHCOO–)_); 66.0 (–COO**C**H**_2_**CH–); 63.5 (–COOCH_2_**C**H–); 53.7 (–CH_2_N^+^–); 51.8 (–CH_3_); 47.6 and 46.7 (–NH^+^(**C**H_2_CH_3_)_2_); 8.5 and 8.3 (–NH^+^(CH_2_**C**H_3_)_2_); HR-MS: C_16_H_23_N_2_O_5_ [M − H]^−^ calculated 323.1612 *m*/*z*; found 323.1612 *m*/*z.*

*(3-{4-[(Ethoxycarbonyl)amino]benzoyloxy}-2-hydroxypropyl)diethylammonium chloride* (**5f**). Yield: 71%; R_f_: 0.83 (ethyl acetate/diethylamine 10:1); R_f_ (rev.): 0.61 (0.1 M HCl/acetone 3:2); m.p.: 153–156 °C; HPLC pur. 98.17% (254 nm); IR (Zn/Se ATR): ν (cm^−1^) = 3326 w; 2985 w; 2608 w; 1740 s; 1685 s; 1595 s; 1536 s; 1216 s; 1177 s; ^1^H-NMR (700 MHz, DMSO-*d_6_*): δ (ppm) = 10.08 (s, 1H, –NHCOO–); 10.02 (bs, 1H, NH^+^); 7.95 (d, *J* = 8.8 Hz, 2H, –C**H**_Ar(–COO–_); 7.62 (d, *J* = 8.8 Hz, 2H, –C**H**_Ar(–NHCOO–)_); 6.01 (d, *J* = 5.1 Hz, 1H, –OH), 4.34 (m, 1H, –COOCH_2_C**H**–); 4.23 (m, 2H, –COOC**H_2_**CH–); 4.15 (q, *J* = 7.1 Hz, 2H, –C**H_2_**CH_3_); 3.27–3.14 (overlap m, 6H, –(C**H_2_**)NH^+^(C**H_2_**CH_3_)_2_); 1.27–1.22 (overlap m, 9H, –CH_2_C**H**_3_ + –NH^+^(CH**_2_**C**H_3_**)_2_); ^13^C-NMR (176 MHz, DMSO-*d_6_*): δ (ppm) = 165.10 (–COO–); 153.29 (–NHCOO–); 143.97 (–**C**_Ar(–NHCOO–)_); 130.51 (–**C**H_Ar(–COO–)_); 122.82 (–**C**_Ar(–COO–)_); 117.28 (–**C**H_Ar(–NHCOO–)_); 66.03 (–COO**C**H**_2_**CH–); 63.49 (–COOCH_2_**C**H–); 60.48 (–**C**H_2_CH_3_); 53.68 (–CH_2_N^+^–); 47.59 and 46.70 (–NH^+^(**C**H_2_CH_3_)_2_); 14.36 (–CH_3_); 8.44 and 8.24 (–NH^+^(CH_2_**C**H_3_)_2_); HR-MS: C_17_H_25_N_2_O_5_ [M − H]^−^ calculated 337.1769 *m*/*z*; found 337.1771 *m*/*z.*

#### 3.2.5. Synthesis of Quaternary Ammonium Salts as Final Compounds

Firstly, the salt was converted to a free base. Hydrochlorides **5a**–**f** were dissolved in water, NaHCO_3_ (5 M) was added and the mixture was extracted with chloroform. The organic layer was dried over anhydrous Na_2_CO_3_, filtered and chloroform was evaporated under vacuum which provided the residue in a form of yellow oily liquid. The obtained base **4a**–**d** (0.01 mol), pre-dried chlorobenzene (50 mL) and the appropriate alkylating agent (0.013 mol) were added to the boiling flask and reaction was then carried out in a microwave reactor. For the specific reaction conditions see Table 1. When the microwave reaction ended, the solution was allowed cooled down to −20 °C from 14 to 48 h and the solid compound precipitated. The crystals of the quaternary ammonium salts were filtered, washed with diethyl ether and recrystallized with ethanol. See the ^1^H and ^13^C-NMR spectra of compounds **6a**–**i** as Supplementary Material (Figures S14–S31).

*(2-Hydroxy-3-{4-[(methoxycarbonyl)amino]benzoyloxy}propyl)trimethylammonium iodide* (**6a**). Yield: 76%; R_f_: 0.38 (*n*-PrOH/EtOH/HOAc/H_2_O 8:4:4:3); R_f_ (rev.): 0.68 (0.1 M HCl/acetone 3:2); m.p.: 190–193 °C; HPLC pur. 96.40% (254 nm); IR (Zn/Se ATR): ν (cm^−1^) = 3330 w, 1717 s, 1702 s, 1600 m, 1538 s, 1415 m, 1322 m, 1211 s, 1179 m, 1130 w; ^1^H-NMR (700 MHz, DMSO-*d_6_*): δ (ppm) = 10.04 (s, 1H, –NHCOO–); 7.97 (d, *J* = 8.8 Hz, 2H, –C**H**_Ar(–COO–)_); 7.61 (d, *J* = 8.8 Hz, 2H, –C**H**_Ar(–NHCOO–)_); 5.77 (d, *J* = 5.1 Hz, 1H, –OH); 4.44 (m, 1H, –COOCH_2_C**H**–); 4.21 (m, 2H, –COOC**H_2_**CH–); 3.70 (s, 3H, –CH_3_); 3.54 (m, 2H, –CH_2_N^+^–); 3.19 (s, 9H, –N^+^(CH_3_)_3_); ^13^C-NMR (176 MHz, DMSO-*d*_6_): δ (ppm) = 165.00 (–COO–); 153.68 (–NHCOO–); 143.85 (–**C**_Ar(–NHCOO–)_); 130.56 (–**C**H_Ar(–COOH)_); 122.77 (–**C**_Ar(–COOH)_); 117.25 (–**C**H_Ar(–NHCOO–)_); 67.55 (–CH_2_N^+^–); 66.20 (–COO**C**H**_2_**CH–); 63.52 (–COOCH_2_**C**H–); 53.47 (–N^+^(CH_3_)_3_); 51.83 (–CH_3_); HR-MS: C_15_H_23_N_2_O_5_ [M^+^] calculated 311.1644 *m*/*z*; found 311.1623 *m*/*z.*

*Ethyl(2-hydroxy-3-{4-[(methoxycarbonyl)amino]benzoyloxy}propyl)dimethylammonium iodide* (**6b**). Yield: 75%; R_f_: 0.35 (*n*-PrOH/EtOH/HOAc/H_2_O 8:4:4:3); R_f_ (rev.): 0.63 (0.1 M HCl/acetone 3:2); m.p.: 198–201 °C; HPLC pur. 96.11% (254 nm); IR (Zn/Se ATR): ν (cm^−1^) = 3310 w, 1728 s, 1720 s, 1593 m, 1531 s, 1263 m, 1220 s, 1175 s, 1123 m, 1059 m; ^1^H-NMR (700 MHz, DMSO-*d_6_*): δ (ppm) = 10.08 (s, 1H, –NHCOO–); 7.97 (d, *J* = 8.8 Hz, 2H, –C**H**_Ar(–COOH)_); 7.61 (d, *J* = 8.8 Hz, 2H, –C**H**_Ar(–NHCOO–)_); 5.79 (bs, 1H, –OH); 4.43 (m, 1H, –COOCH_2_C**H**–); 4.21 (m, 2H, –COOC**H_2_**CH–); 3.70 (s, 3H, –CH_3_); 3.49–3.46 (overlap m, 4H, –C**H_2_**N^+^C**H_2_**CH_3_); 3.11 (s, 3H, –N^+^CH_3_); 3.10 (s, 3H, –N^+^CH_3_); 1.28 (t, *J* = 7.1 Hz, 3H, –N^+^CH_2_C**H_3_**); ^13^C-NMR (176 MHz, DMSO-*d_6_*): δ (ppm) = 165.05 (–COO–); 153.72 (–NHCOO–); 143.90 (–**C**_Ar(–NHCOO–)_); 130.62 (–**C**H_Ar(–COO–)_); 122.78 (–**C**_Ar(–COO–)_); 117.25 (–**C**H_Ar(–NHCOO–)_); 66.28 (–COO**C**H_2_CH–); 64.78 (–CH_2_N^+^–); 63.29 (–COOCH_2_**C**H–); 59.99 (–N^+^**C**H_2_CH_3_); 51.88 (–CH_3_); 50.67 and 50.46 (–N^+^(CH_3_)_2_); 7.92 (–N^+^CH_2_**C**H_3_); HR-MS: C_16_H_25_N_2_O_5_ [M^+^] calculated 325.1753 *m*/*z*; found 325.1318 *m*/*z.*

*(2-Hydroxy-3-{4-[(methoxycarbonyl)amino]benzoyloxy}propyl)dimethyl(propan-2-yl)ammonium iodide* (**6c**). Yield: 70%; R_f_: 0.39 (*n*-PrOH/EtOH/HOAc/H_2_O 8:4:4:3); R_f_ (rev.): 0.60 (0.1 M HCl/acetone 3:2); m.p.: 164–167 °C; HPLC pur. 98.49% (254 nm); IR (Zn/Se ATR): ν (cm^−1^) = 3311 w, 1702 s, 1698 s, 1593 m, 1528 s, 1220 s, 1174 s, 1698 s, 1528 s, 1220 m; ^1^H-NMR (700 MHz, DMSO-*d_6_*): δ (ppm) = 10.07 (s, 2H, –NHCOO–); 7.97 (d, *J* = 8.8 Hz, 2H, –C**H**_Ar(–COO–)_); 7.61 (d, *J* = 8.8 Hz, 2H, –C**H**_Ar(–NHCOO–)_); 5.81 (d, *J* = 5.5 Hz, 2H, –OH); 4.46 (m, 2H, –COOCH_2_C**H**–); 4.22 (m, 2H, –COOC**H_2_**CH–); 3.86 (m, 1H, –C**H**(CH_3_)_2_); 3.70 (s, 3H, –CH_3_); 3.46 (m, 2H, –CH_2_N^+^–); 3.07 (s, 3H, –N^+^CH_3_); 3.05 (s, 3H, –N^+^CH_3_); 1.33 (m, 6H, –CH(C**H_3_**)_2_); ^13^C-NMR (176 MHz, DMSO-*d_6_*): δ (ppm) = 165.06 (–COO–); 153.72 (–NHCOO–); 143.88 (–**C**_Ar(–NHCOO–)_); 130.60 (–**C**H_Ar(–COO–)_); 122.82 (–**C**_Ar(–COO–)_); 117.27 (–**C**H_Ar(–NHCOO–)_); 67.24 (–CH_2_N^+^–); 66.39 (–COO**C**H**_2_**CH–); 63.31 (–COOCH_2_**C**H–); 51.87 (–CH_3_); 48.44 and 47.75 (–N^+^(CH_3_)_2_); 45.65 (–**C**H(CH_3_)_2_); 16.11 and 15.97 (–CH(**C**H_3_)_2_); HR-MS: C_17_H_27_N_2_O_5_ [M^+^] calculated 339.1909 *m*/*z*; found 339.2194 *m*/*z.*

*(3-{4-[(Ethoxycarbonyl)amino]benzoyloxy}-2-hydroxypropyl)trimethylammonium iodide* (**6d**). Yield: 82%; R_f_: 0.41 (*n*-PrOH/EtOH/HOAc/H_2_O 8:4:4:3); R_f_ (rev.): 0.59 (0.1 M HCl/acetone 3:2); m.p.: 204–206 °C; HPLC pur. 97.71% (254 nm); IR (Zn/Se ATR): ν (cm^−1^) = 3321 w, 1698 s, 1597 m, 1538 s, 1417 m, 1318 m, 1223 s, 1178 m, 1063 m; ^1^H-NMR (700 MHz, DMSO-*d_6_*): δ (ppm) = 10.01 (s, 1H, –NHCOO–); 7.97 (d, *J* = 8.8 Hz, 2H, –C**H**_Ar(–COO–)_); 7.62 (d, *J* = 8.8 Hz, 2H, –C**H**_Ar(–NHCOO–)_); 5.77 (d, *J* = 5.3 Hz, 1H, –OH); 4.44 (m, 1H, –COOCH_2_C**H**–); 4.21 (m, 2H, –COOC**H_2_**CH–); 4.15 (q, *J* = 7.1 Hz, 2H, –C**H_2_**CH_3_); 3.52 (m, 2H, –CH_2_N^+^–); 3.19 (s, 9H, –N^+^(CH_3_)_3_); 1.26 (t, *J* = 7.2 Hz, 3H, –CH_2_C**H**_3_); ^13^C-NMR (176 MHz, DMSO-*d_6_*): δ (ppm) = 165.00 (–COO–); 153.22 (–NHCOO–); 143.93 (–**C**_Ar(–NHCOO–)_); 130.54 (–**C**H_Ar(–COO–)_); 122.68 (–**C**_Ar(–COO–)_); 117.23 (–**C**H_Ar(–NHCOO–)_); 67.55 (–CH_2_N^+^–); 66.18 (–COO**C**H**_2_**CH–); 63.51 (–COOCH_2_**C**H–); 60.45 (–**C**H_2_CH_3_); 53.46 (–N^+^(CH_3_)_3_); 14.30 (–CH_3_); HR–MS: C_16_H_25_N_2_O_5_ [M^+^] calculated 325.1753 *m*/*z*; found 325.1814 *m*/*z.*

*(3-{4-[(Ethoxycarbonyl)amino]benzoyloxy}-2-hydroxypropyl)(ethyl)dimethylammonium iodide* (**6e**) Yield: 71%; R_f_: 0.37 (*n*-PrOH/EtOH/HOAc/H_2_O 8:4:4:3); R_f_ (rev.): 0.54 (0.1 M HCl/acetone 3:2); m.p.: 193–196 °C; HPLC pur. 97.26% (254 nm); IR (Zn/Se ATR): ν (cm^−1^) = 3325 w, 1728 s, 1681 s, 1598 m, 1538 m, 1225 s, 1179 m, 1065 m; ^1^H-NMR (700 MHz, DMSO-*d_6_*): δ (ppm) = 10.05 (s, 1H, –NHCOO–); 7.96 (d, *J* = 8.8 Hz, 2H, –C**H**_Ar(–COO–)_); 7.62 (d, *J* = 8.8 Hz, 2H, –C**H**_Ar(–NHCOO–)_); 5.80 (bs, 1H, –OH); 4.43 (m, 1H, –COOCH_2_C**H**–); 4.21 (m, 2H, –COOC**H_2_**CH–); 4.16 (q, *J* = 7.1 Hz, 2H, –C**H_2_**CH_3_); 3.49–3.46 (overlap m, 4H, –C**H_2_**N^+^C**H_2_**CH_3_); 3.12 (s, 3H, –N^+^CH_3_); 3.11 (s, 3H, –N^+^CH_3_); 1.29–1.25 (overlap m, 6H, –CH_2_C**H**_3_ + –N^+^CH**_2_**C**H_3_**); ^13^C NMR (176 MHz, DMSO-*d*_6_): δ (ppm) = 165.05 (–COO–); 153.25 (–NHCOO–); 143.99 (–**C**_Ar(–NHCOO–)_); 130.60 (–**C**H_Ar(–COO–)_); 122.70 (–**C**_Ar(–COO–)_); 117.23 (–**C**H_Ar(–NHCOO–)_); 66.28 (–COO**C**H**_2_**CH–); 64.77 (–CH_2_N^+^–); 63.28 (–COOCH_2_**C**H–); 60.49 (–**C**H_2_CH_3_); 59.98 (–N^+^(**C**H_2_CH_3_); 50.67 and 50.46 (–N^+^(CH_3_)_2_); 14.36 (–CH_3_); 7.92 (–N^+^CH_2_**C**H_3_); HR-MS: C_17_H_27_N_2_O_5_ [M^+^] calculated 339.1909 *m*/*z*; found 339.1906 *m*/*z.*

*(3-{4-[(Ethoxycarbonyl)amino]benzoyloxy}-2-hydroxypropyl)diethylmethylammonium iodide* (**6f**). Yield: 55%; R_f_: 0.44 (*n*-PrOH/EtOH/HOAc/H_2_O 8:4:4:3); R_f_ (rev.): 0.58 (0.1 M HCl/acetone 3:2); m.p.: 173–176 °C; HPLC pur. 99.58% (254 nm); IR (Zn/Se ATR): ν (cm^−1^) = 3320 w, 1703 s, 1690 s, 1591 s, 1537 s, 1416 m, 1224 s, 1178 m, 1063 m; ^1^H-NMR (700 MHz, DMSO-*d_6_*): δ (ppm) = 10.05 (s, 1H, –NHCOO–); 7.96 (d, *J* = 8.8 Hz, 2H, –C**H**_Ar(–COO–)_); 7.61 (d, *J* = 8.8 Hz, 2H, –C**H**_Ar(–NHCOO–)_); 5.77 (d, *J* = 5.4 Hz, 1H, –OH), 4.42 (m, 1H, –COOCH_2_C**H**–); 4.21 (m, 2H, –COOC**H_2_**CH–); 4.15 (q, *J* = 7.1 Hz, 2H, –C**H_2_**CH_3_); 3.48–3.40 (overlap m, 6H, –(C**H_2_**)N^+^(C**H_2_**CH_3_)_2_); 3.04 (s, 3H, –N^+^CH_3_); 1.26–1.24 (overlap m, 9H, –CH_2_C**H**_3_ + –N^+^(CH**_2_**C**H_3_**)_2_); ^13^C-NMR (176 MHz, DMSO-*d_6_*): δ (ppm) = 165.06 (–COO–); 153.27 (–NHCOO–); 143.99 (–**C**_Ar(–NHCOO–)_); 130.60 (–**C**H_Ar(–COO–)_); 122.70 (–**C**_Ar(–COO–)_); 117.25 (–**C**H_Ar(–NHCOO–)_); 66.28 (–COO**C**H**_2_**CH–); 63.09 (–COOCH_2_**C**H–); 61.94 (–CH_2_N^+^–); 60.53 (–**C**H_2_CH_3_); 56.80 and 56.68 (–N^+^(**C**H_2_CH_3_)_2_); 47.59 (–N^+^CH_3_); 14.38 (–CH_3_); 7.64 (–N^+^(CH_2_**C**H_3_)_2_); HR-MS: C_18_H_29_N_2_O_5_ [M^+^] calculated 353.2065 *m*/*z*; found 353.2082 *m*/*z.*

*(2-Hydroxy-3-{4-[(propoxycarbonyl)amino]benzoyloxy}propyl)trimethylammonium iodide* (**6g**). Yield: 58%; R_f_: 0.44 (*n*-PrOH/EtOH/HOAc/H_2_O 8:4:4:3); R_f_ (rev.): 0.53 (0.1 M HCl/acetone 3:2); m.p.: 149–151 °C; HPLC pur. 98.33% (254 nm); IR (Zn/Se ATR): ν (cm^−1^) = 3311 w, 1697 s, 1594 s, 1536 s, 1415 m, 1274 m, 1217 s, 1178 m, 1069 m; ^1^H-NMR (700 MHz, DMSO-*d_6_*): δ (ppm) = 10.05 (s, 1H, –NHCOO–); 7.97 (d, *J* = 8.9 Hz, 2H, –C**H**_Ar(–COO–)_); 7.62 (d, *J* = 8.9 Hz, 2H, –C**H**_Ar(–NHCOO–)_); 5.79 (d, *J* = 5.5 Hz, 1H, –OH); 4.44 (m, 1H, –COOCH_2_C**H**–); 4.20 (m, 2H, –COOC**H_2_**CH–); 4.06 (t, *J* = 6.7 Hz, 2H, –C**H_2_**CH_2_CH_3_); 3.52 (m, 2H, –CH_2_N^+^–); 3.19 (s, 9H, –N^+^(CH_3_)_3_); 1.65 (m, 2H, –CH_2_C**H_2_**CH_3_); 0.93 (t, *J* = 7,4 Hz, 3H, –CH_2_CH**_2_**C**H_3_**); ^13^C-NMR (176 MHz, DMSO-*d*_6_): δ (ppm) = 165.07 (–COO–); 153.37 (–NHCOO–); 144.01 (–**C**_Ar(–NHCOO–)_); 130.64 (–**C**H_Ar(–COO–)_); 122.70 (–**C**_Ar(–COO–)_); 117.25 (–**C**H_Ar(–NHCOO–)_); 67.54 (–CH_2_N^+^–); 66.26 (–COO**C**H**_2_**CH–); 66.02 (–**C**H_2_CH_2_CH_3_); 63.53 (–COOCH_2_**C**H–); 53.47 (–N^+^(CH_3_)_3_); 21.75 (–CH_2_**C**H_2_CH_3_); 10.18 (–CH_3_); ^15^N-NMR (DMSO-*d*_6_): δ (ppm) = 48.2 (–N^+^(CH_3_)_2_); 109.1 (–NHCOO–); HR-MS: C_17_H_27_N_2_O_5_ [M^+^] calculated 339.1909 *m*/*z*; found 339.1870 *m*/*z.*

*Diethyl (2-hydroxy-3-{4-[(propoxycarbonyl)amino]benzoyloxy}propyl)methylammonium iodide* (**6h**). Yield: 49%; R_f_: 0.46 (*n*-PrOH/EtOH/HOAc/H_2_O 8:4:4:3); R_f_ (rev.): 0.53 (0.1 M HCl/acetone 3:2); m.p.: 70–73 °C; HPLC pur. 98.80% (254 nm); IR (Zn/Se ATR): ν (cm^−1^) = 3311 w, 2946 s, 1713 s, 1589 s, 1533 s, 1501 s, 1399 m, 1224 s, 1205 s, 1191 m, 1031 m; ^1^H-NMR (700 MHz, DMSO-*d_6_*): δ (ppm) = 10.05 (s, 1H, –NHCOO–); 7.96 (d, *J* = 8.9 Hz, 2H, –C**H**_Ar(–COO–)_); 7.62 (d, *J* = 8.9 Hz, 2H, –C**H**_Ar(–NHCOO–)_); 5.78 (d, *J* = 5.5 Hz, 1H, –OH), 4.42 (m, 1H, –COOCH_2_C**H**–); 4.22 (m, 2H, –COOC**H_2_**CH–); 4.06 (t, *J* = 6.7 Hz, 2H, –C**H_2_**CH_2_CH_3_); 3.49–3.39 (overlap m, 6H, –C**H_2_**N^+^(C**H_2_**CH_3_)_2_); 3.04 (s, 3H, –N^+^CH_3_); 1.65 (m, 2H, –CH_2_C**H_2_**CH_3_), 1.25 (m, 6H, –N^+^(CH**_2_**C**H_3_**)_2_), 0.93 (t, *J* = 7.4 Hz, 3H, –CH_2_CH_2_C**H_3_**); ^13^C-NMR (176 MHz, DMSO-*d_6_*): δ (ppm) = 165.08 (–COO–); 153.38 (–NHCOO–); 144.00 (–**C**_Ar(–NHCOO–)_); 130.60 (–**C**H_Ar(–COO–)_); 122.73 (–**C**_Ar(–COO–)_); 117.29 (–**C**H_Ar(–NHCOO–)_); 66.30 (–COO**C**H**_2_**CH–); 66.03 (–**C**H_2_CH_2_CH_3_); 63.09 (–COOCH_2_**C**H–); 61.96 (–CH_2_N^+^–); 56.82 and 56.69 (–N^+^(**C**H_2_CH_3_)_2_); 47.61 (–N^+^CH_3_); 21.76 (–CH_2_**C**H_2_CH_3_); 10.18 (–CH_3_); 7.64 (–N^+^(CH_2_**C**H_3_)_2_); HR-MS: C_19_H_31_N_2_O_5_ [M^+^] calculated 367.2222 *m*/*z*; found 367.2933 *m*/*z.*

*(3-{4-[(Butoxycarbonyl)amino]benzoyloxy}-2-hydroxypropyl)trimethylammonium iodide* (**6i**). Yield: 45%; R_f_: 0.45 (*n*-PrOH/EtOH/HOAc/H_2_O 8:4:4:3); R_f_ (rev.): 0.47 (0.1 M HCl/acetone 3:2); m.p.: 93–96 °C; HPLC pur. 88.42% (254 nm); IR (Zn/Se ATR): ν (cm^−1^) = 3317 w, 2958 w, 1710 s, 1699 m, 1597 s, 1544 s, 1410 m, 1236 s, 1172 m, 1068 m; ^1^H-NMR (700 MHz, DMSO-*d_6_*): δ (ppm) = 10.04 (s, 1H, –NHCOO–); 7.97 (d, *J* = 8.9 Hz, 2H, –C**H**_Ar(–COO–)_); 7.62 (m, *J* = 8.8 Hz, 2H, –C**H**_Ar(–NHCOO–)_); 5.80 (d, *J* = 5.1 Hz, 1H, –OH), 4.44 (m, 1H, –COOCH_2_C**H**–); 4.20 (m, 2H, –COOC**H_2_**CH–); 4.11 (t, *J* = 6.6 Hz, 2H, –C**H_2_**CH_2_CH_2_CH_3_); 3.52 (m, 2H, –C**H_2_**N^+^–); 3.19 (s, 9H, –N^+^(CH_3_)_3_); 1.61 (m, 2H, –CH_2_C**H_2_**CH_2_CH_3_), 1.38 (m, 2H, –CH_2_CH_2_C**H_2_**CH_3_), 0.92 (t, *J* = 7.4 Hz, 3H, –CH_2_CH_2_CH_2_C**H_3_**); ^13^C-NMR (176 MHz, DMSO-*d_6_*): δ (ppm) = 165.07 (–COO–); 153.37 (–NHCOO–); 144.01 (–**C**_Ar(–NHCOO–)_); 130.64 (–**C**H_Ar(–COO–)_); 122.70 (–**C**_Ar(–COO–)_); 117.25 (–**C**H_Ar(–NHCOO–)_); 67.55 (–CH_2_N^+^–); 66.26 (–COO**C**H**_2_**CH–); 64.22 (–**C**H_2_CH_2_CH_2_CH_3_); 63.53 (–COOCH_2_**C**H–); 53.47 (–N^+^(CH_3_)_3_); 30.43 (–CH_2_**C**H_2_CH_2_CH_3_); 18.52 (–CH_2_CH_2_**C**H_2_CH_3_); 13.52 (–CH_3_); HR-MS: C_18_H_29_IN_2_O_5_ [M^+^] calculated 353.2066 *m*/*z*; found 353.2082 *m*/*z.*

### 3.3. Molecular Modelling

*X*-ray structure of *Torpedo californica* acetylcholinesterase available in the Protein Data Bank (http://www.rcsb.org; PDB code: 1DX6) [35] was used as the receptor for molecular docking studies. Water and ligand molecules were removed from PDB structure before calculations. From the QTAIM [16] point of view, two interacting atoms shares three topological elements related to each other, a point, a line and a surface. The first element is the bond critical point (BCP), namely the critical point in *ρ*(r) topology that is found between any two interacting nuclei. From each BCP, two unique trajectories of gradient vectors of electronic density, ∇*ρ*(r), originate at that point and terminate at each of the neighbouring nuclei. These trajectories define a line along which *ρ*(r) is a maximum with respect to any neighbouring line. This line, that constitutes the second element, is the bond path, BP. Additionally, the set of trajectories that terminate at a BCP define the interatomic surface that separates the atomic basins of the neighboring atoms [18,19,20,21].

#### 3.3.1. Molecular Docking

The Automatic docking was carried out using the AutoDock version 4.0 [14]. During the docking procedures, water molecules and ligands were removed from the protein. The receptor structure was defined as rigid. The grid dimensions were 60, 60, and 60 for the X, Y, and Z axes, respectively in the active site of the AChE with a resolution of 0.375 Å. Gasteiger charges were assigned for all the compounds and nonpolar hydrogen atoms were merged. All torsions of the ligand were allowed to rotate during docking. All graphic manipulations and visualizations were performed using the AutoDock Tools 1.5.4 and ligand docking with the AutoDock version 4.0 [14]. A total of 200 accepted conformations were collected. Other parameters were set to default values. The docking results were clustered on the basis of root-mean square deviation (rmsd) between the Cartesian coordinates of the ligand atoms and were ranked according to the binding free energy. The structure with relative lower binding free energy and the most cluster members was chosen as the optimum docking conformation.

#### 3.3.2. Molecular Dynamics (MD) Simulations

A series of MD simulations were carried out for all complexes obtained under docking procedures (see Section 2.2). The simulations and the analysis of the trajectories were performed with Amber 12 package [15] using the FF99SB force field [36]. Complexes were constructed using the Leap module, minimized by the sander module, and the “pmemd” module was used for MD simulations. The ligand parameters were estimated with the antechamber module, based on the general AMBER force field (GAFF) [37]. Na^+^ ions were placed by Leap to neutralize the negative charges of areas around the complex model. Complexes were subsequently solvated in a truncated octahedral periodic box using the TIP3P water model [38], with a margin of 10.0 Å in each direction from the solute. The systems were then equilibrated by a 500 ps of energy minimization with position restraints on the protein and ligand to allow for relaxation of the solvent molecules. The equilibration run was followed by a 10 ns MD run without position restraints under periodic boundary conditions.

The non-bonded list was generated using an atom-based cutoff of 8.0 Å. The electrostatic term was described with the particle mesh Ewald algorithm [39]. The time step of the MD simulations was set to 2.0 fs, and the SHAKE algorithm [40] was used to constrain bond lengths at their equilibrium values. All production simulations were performed under NVT conditions. Temperature was maintained at 298 K using the Langevin thermostat [41] with collision frequency of 1 ps^−1^. Coordinates were saved for analyses every 10 ps. Analysis of the trajectories was performed using AMBER analysis tools [15] and PyMOL [42].

#### 3.3.3. Atoms in Molecules Theory

The wave functions of the inhibitors bound to the binding site residues (residues that have at least one heavy atom within 5 Å from the ligand molecule (first shell residues)), generated at the M062X/6-31G(d) level of theory, were subjected to a Quantum Theory Atoms In Molecules (QTAIM) analysis [16] using the Multiwfn software (Sobereva, v. 3.3.9.) [43]. This type of calculations was being used in recent works because it ensures a reasonable compromise between the wave function quality required to obtain reliable values of the derivatives of ρ_(r)_ and the computer power available, due to the extension of the systems in study [18,19,20,21,22].

## 4. Conclusions

Six hydrochlorides of tertiary amines and nine new QUATs have been synthesized. The general structure of synthesized compounds was that of an arylcarbonyloxyaminopropanol with a carbamate group in the *para* position. For tertiary amines, the lipophilicity and acid-base dissociation constant were measured and values were compared with software-calculated data. QUATs were determined by lipophilicity software calculated data and by surface tension. Finally, QUATs **6f**,**h** had promising activity to inhibit acetylcholinesterase enzyme and their structure will be the clue to synthesize more active compounds, particularly the enantiomers of these compounds. Another interesting contribution of this work is the knowledge of some details about structural aspects which are essential to understand the formation of the QUATs-AChE complexes. Thus, our molecular modelling study provides interesting information on possibilities of structural modification of these compounds in order to enhance their biological effect as well as to help in designing new inhibitors of AChE.

## Figures and Tables

**Table 1:** Reaction conditions in microwave reactor related to alkylation of tertiary amines.

Final QUAT	Alkylating Agent	Time (min)	Temperature (°C)	Power (Watt)
**6a**	MeI	15	120	400
**6b**	EtI	20	120	400
**6c**	*i*PrI	60	125	600
**6d**	MeI	15	120	400
**6e**	EtI	20	120	400
**6f**	MeI	40	125	600
**6g**	MeI	15	120	400
**6h**	MeI	40	125	600
**6i**	MeI	15	120	400

**Table 2:** The ability of compounds to inhibit AChE and BuChE.

Compound	AChE ^a^ (%)	AChE IC_50_ (μM)	BuChE ^a^ (%)	BuChE IC_50_ (μM)
**5a**	31.9 ± 2.7	>100	14.8 ± 2.9	>100
**5b**	33.4 ± 2.8	>100	8.7 ± 4.8	>100
**5c**	41.3 ± 3.1	>100	18.4 ± 7.1	>100
**5d**	50.9 ± 2.2	>100	17.8 ± 3.9	>100
**5e**	42.4 ± 3.3	>100	8.3 ± 1.9	>100
**5f**	52.7 ± 2.0	>100	16.2 ± 4.9	>100
**6a**	16.9 ± 1.0	>100	16.4 ± 4.7	>100
**6b**	53.2 ± 1.6	100.0	13.8 ± 2.6	>100
**6c**	59.9 ± 2.7	>100	27.7 ± 1.7	>100
**6d**	52.6 ± 1.5	>100	20.0 ± 1.0	>100
**6e**	58.7 ± 1.0	>100	18.3 ± 1.5	>100
**6f**	64.9 ± 1.7	56.0	19.8 ± 5.7	>100
**6g**	43.0 ± 1.6	>100	21.4 ± 5.6	>100
**6h**	70.6 ± 1.5	58.0	32.9 ± 2.7	>100
**6i**	48.6 ± 1.6	>100	34.1 ± 3.5	>100
**Rivastigmine**	n.d.	5.5 [13]	n.d.	1.6 [13]
**Galantamine**	87.1 ± 1.3	1.1	62.1 ± 1.4	6.8

^a^ Percentage of inhibition at 100 μM expressed as the mean ± SD (*n* = 5); n.d.—not determined.

**Table 3:** Experimentally determined and calculated values of lipophilicity indexes (log *k_w_*) in comparison with software-calculated values of distribution coefficients (log *D*^9.0^) and lipophilicity (log *P*) of tertiary amines. Experimentally determined p*K*_a_ in comparison with calculated values of tertiary amines.

Compounds	log *P* ^a^	log *P* ^b^	log *D*^9.0 a^	log *k_w_* ^c^	log *k_w_*^9.0 c^	p*K*_a_^a^	p*K*_a_^b^	p*K*_a_^c^
**5a**	1.22	1.84 ± 0.57	0.97	0.57 ± 0.08	0.42 ± 0.01	8.90	8.60 ± 0.3	8.62 ± 0.03
**5b**	1.58	2.37 ± 0.57	1.33	0.96 ± 0.12	0.84 ± 0.02	8.90	8.60 ± 0.3	8.52 ± 0.04
**5c**	2.10	2.91 ± 0.57	1.85	1.48 ± 0.13	1.36 ± 0.02	8.90	8.60 ± 0.3	8.52 ± 0.04
**5d**	2.55	3.44 ± 0.57	2.29	2.08 ± 0.15	1.97 ± 0.02	8.90	8.60 ± 0.3	8.44 ± 0.04
**5e**	1.94	2.91 ± 0.57	1.31	1.23 ± 0.08	0.71 ± 0.01	9.46	9.50 ± 0.3	9.35 ± 0.03
**5f**	2.29	3.44 ± 0.57	1.71	1.66 ± 0.10	1.14 ± 0.02	9.46	9.50 ± 0.3	9.36 ± 0.03

^a^ Calculated by MarvinSketch 17.2.20.; ^b^ calculated by ACD Percepta, ver. 2012; ^c^ experimental results.

**Table 4:** Physicochemical properties determination of QUATs.

Compound	log P ^a^	σ	%
**6a**	−2.94	0.07269	n.d. ^b^
**6b**	−2.58	0.07310	n.d. ^b^
**6c**	−2.17	0.07080	2.47
**6d**	−2.58	0.07331	n.d. ^b^
**6e**	−2.23	0.07252	0.10
**6f**	−1.87	0.72400	0.26
**6g**	−2.06	0.07235	0.33
**6h**	−1.35	0.07234	0.33
**6i**	−1.62	0.07210	0.68

^a^ Calculated by MarvinSketch 6.0.6.; n.d.—not determined; ^b^ compounds increased surface tension.

## Supplementary Materials

Supplementary materials are available online.

## References

[1] World Health Organization (WHO) Dementia. http://www.who.int/mediacentre/factsheets/fs362/en/.

[2] Pisani L., Farina R., Catto M., Iacobazzi R.M., Nicolotti O., Cellamare S., Mangiatordi G.F., Denora Nunzio S.R., Siragusa L., Altomare C.D. (2016). Exploring Basic Tail Modifications of Coumarin-Based Dual Acetylcholinesterase-Monoamine Oxidase B Inhibitors: Identification of Water-Soluble, Brain-Permeant Neuroprotective Multitarget Agents. J. Med. Chem..

[3] Reitz Ch., Brayne C., Mayeux R. (2011). Epidemiology of Alzheimer disease. Nat. Rev. Neurol..

[4] Novak P., Schmidt R., Kontsekova E., Zilka N., Kovacech B., Skrabana R., Vince-Kazmerova Z., Katina S., Fialova L., Prcina M. (2017). Safety and immunogenicity of the tau vaccine AADvac1 in patients with Alzheimer’s disease: A randomised, double-blind, placebo-controlled, phase 1 trial. Lancet Neurol..

[5] Wang D.-M., Feng B., Fu H., Liu A.L., Wang L., Du G.H., Wu S. (2017). Design, Synthesis, and Biological Evaluation of a New Series of Biphenyl/Bibenzyl Derivatives Functioning as Dual Inhibitors of Acetylcholinesterase and Butyrylcholinesterase. Molecules.

[6] De Oliveira J.S., Husein A.F., Lopes D.G., Adeniyi A.S., Vidal P.T., Signor C., Da Silva B.J., Baldissarelli J., Suéling L.L., Pereira M.R. (2016). Berberine protects against memory impairment and anxiogenic-like behavior in rats submitted to sporadic Alzheimer’s-like dementia: Involvement of acetylcholinesterase and cell death. NeuroToxicology.

[7] Dvir H., Silman I., Harel M., Rossenbery T., Sussman J. (2010). Acetylcholinesterase: From 3D structure to function. Chem.-Biol. Interact..

[8] Padrtova T., Marvanova P., Mokry P. (2017). Quaternary Ammonium Salts—Synthesis and Use. Chem. Listy.

[9] Mokry P., Zemanova M., Csöllei J., Racanska E., Tumova I. (2003). Synthesis and pharmacological evaluation of novel potential ultrashort-acting β-blockers. Pharmazie.

[10] Connors S.P., Dennis P.D., Gill E.W., Terrar D.A. (1991). The Synthesis and Potassium Channel Blocking Activity of some (4-Methanesulfonamidophenoxy)propanolamines as Potential Class III Antiarrhythmic Agents. J. Med. Chem..

[11] Acevedo O., Jorgensen W.L. (2010). Exploring Solvent Effects upon the Menshutkin Reaction Using a Polarizable Force Field. J. Phys. Chem..

[12] Fan P., Terrier L., Hay A.-E., Marston A., Hosttetmann K. (2010). Antioxidant and enzyme inhibition activities and chemical profiles of *Polygonum sachalinensis* F.Schmidt ex Maxim (*Polygonaceae*). Fitoterapia.

[13] Xie Q., Zheng Z., Shao B., Fu W., Xia Z., Li W., Sun J., Zheng W., Zhang W., Sheng W. (2017). Pharmacophore-based design and discovery of (−)-meptazinol carbamates as dual modulators of cholinesterase and amyloidogenesis. J. Enzym. Inhib. Med. Chem..

[14] Morris G., Huey R., Lindstrom W., Sanner M., Belew R., Goodsell D., Olson A. (2009). AutoDock4 and AutoDockTools4: Automated docking with selective receptor flexibility. J. Comput. Chem..

[15] Case D.A., Darden T.A., Cheatham T.E., Simmerling C.L., Wang J., Duke R.E., Luo R., Walker R.C., Zhang W., Merz K.M. (2012). AMBER 12 OR.

[16] Bader R. (1994). Atoms in Molecules: A Quantum Theory.

[17] Graham L.P. (2009). An Introduction to Medicinal Chemistry.

[18] Andujar S., Tosso R., Suvire F., Angelina E., Peruchena N., Cabedo N., Cortes D., Enriz D. (2012). Searching the “Biologically Relevant” Conformation of dopamine: A computational approach. J. Chem. Inf. Model..

[19] Tosso Rodrigo D., Andujar S.A., Gutierrez L., Angelina E., Rodríguez R., Nogueras M., Baldoni H., Suvire F.D., Cobo J., Enriz D. (2013). Molecular modeling study of dihydrofolate reductase inhibitors. Molecular dynamics simulations, quantum mechanical calculations, and experimental corroboration. J. Chem. Inf. Model..

[20] Párraga J., Cabedo N., Andujar S., Piqueras L., Moreno L., Galán A., Angelina E., Enriz D., Ivorra M.D., Sanz M.J. (2013). 2,3,9- and 2,3,11-Trisubstituted tetrahydroprotoberberines as D_2_ dopaminergic ligands. Eur. J. Med. Chem..

[21] Angelina E., Andujar S., Tosso R., Enriz D., Peruchena N. (2014). Non-covalent interactions in receptor-ligand complexes. A study based on the electron charge density. J. Phys. Org. Chem..

[22] Párraga J., Andujar S.A., Rojas S., Gutierrez L.J., El Aouad N., Sanz M.J., Enriz D., Cabedo N., Cortes D. (2016). Dopaminergic isoquinolines with hexahydrocyclopenta[*ij*]-isoquinolines as D_2_-like selective ligands. Eur. J. Med. Chem..

[23] Bar-On P., Millard C.B., Harel M., Dvir H., Enz A., Sussman J.L., Silman I. (2002). Kinetic and structural studies on the interaction of cholinesterases with the anti-Alzheimer drug rivastigmine. Biochemistry.

[24] Wilson I., Hatch M.A., Ginsburg S. (1960). Carbamylation of acetylcholinesterase. J. Biol. Chem..

[25] Wilson I., Harrison M.A., Ginsburg S. (1961). Carbamyl Derivatives of Acetylcholinesterase. J. Biol. Chem..

[26] Deák K., Takács-Novák K., Kapás M., Vastag M., Tihanyi K., Noszál B. (2008). Physico-chemical characterization of a novel group of dopamine D_3_/D_2_ receptor ligands, potential atypical antipsychotic agents. J. Pharm. Biomed. Anal..

[27] Giaginis C., Tsantilli-Kakoulidou A. (2007). current state of the art in HPLC methodology for lipophilicity assessment of basic drugs. A review. J. Liquid Chromatogr. Relat. Technol..

[28] Lombardo F., Shalaeva M., Bissett B., Chistokhodova N. (2006). Physicochemical and biological profiling in drug research. ElogD^7.4^ 20,000 compounds later: Refinements, observations, and applications. Pharmacokinet. Profil. Drug Res..

[29] Poole S.K., Patel S., Dehring K., Workman H., Poole C.F. (2004). Determination of acid dissociation constants by capillary electrophoresis. J. Chromatogr. A.

[30] Nowak P., Woźniakiewicz M., Kościelniak P. (2015). Application of capillary electrophoresis in determination of acid dissociation constant values. J. Chromatogr. A.

[31] Andrasi M., Buglyo P., Zekany L., Gaspar A. (2007). A comparative study of capillary zone electrophoresis and pH-potentiometry for determination of dissociation constants. J. Pharm. Biomed. Anal..

[32] Tengler J., Kapustikova I., Pesko M., Govender R., Keltosova S., Mokrý P., Kollár P., O´Mahony J., Coffey A., Kráĺová K. (2013). Synthesis and biological evaluation of 2-Hydroxy-3-[(2-aryloxyethyl)amino]propyl 4-[(Alkoxycarbonyl)amino]benzoates. Sci. World J..

[33] Luan F., Ma W., Zhang H., Zhang X., Liu M., Hu Z., Fan B. (2005). Prediction of p*K*_a_ for neutral and basic drugs based on radial basis function neural networks and the heuristic method. Pharm. Res..

[34] (2014). Reference Method for Measuring the Surface Tension of Chromium Electroplating, Chromium Anodizing and Reverse Etching Solutions with a Stalagmometer. https://www.ec.gc.ca/lcpe-cepa/E4899604-74FB-46D1-8E41-E3E24D6A7916/Methode-Reference-Method-eng.pdf.

[35] Greenblatt H.M., Kryger G., Lewis T., Silman I., Sussman J.L. (1999). Structure of acetylcholinesterase complexed with (−)-galanthamine at 2.3 Å resolution. FEBS Lett..

[36] Lindorff-Larsen K., Piana S., Palmo K., Maragakis P., Klepeis J., Dror R., Shaw D. (2010). Improved side-chain torsion potentials for the Amber ff99SB protein force field. Proteins Struct. Funct. Bioinform..

[37] Wang J., Wolf R., Caldwell J., Kollman P., Case D. (2004). Development and testing of a general amber force field. J. Comput. Chem..

[38] Jorgensen W., Chandrasekhar J., Madura J., Impey R., Klein M. (1983). Comparison of simple potential functions for simulating liquid water. J. Chem. Phys..

[39] Essmann U., Perera L., Berkowitz M., Darden T., Lee H., Pedersen L. (1995). A smooth particle mesh Ewald method. J. Chem. Phys..

[40] Ryckaert J.-P., Ciccotti G., Berendsen H. (1977). Numerical integration of the cartesian equations of motion of a system with constraints: Molecular dynamics of n-alkanes. J. Comput. Phys..

[41] Izaguirre J., Catarello D., Wozniak J., Skeel R. (2001). Langevin stabilization of molecular dynamics. J. Chem. Phys..

[42] (2015). The PyMOL Molecular Graphics System, Version 1.8.

[43] Lu T., Chen F. (2012). Multiwfn: A multifunctional wavefunction analyzer. J. Comput. Chem..

