# Supplementary material for: Synthesis, Analysis, Cholinesterase-Inhibiting Activity and Molecular Modelling Studies of 3-(Dialkylamino)-2-hydroxypropyl 4-[(Alkoxy-carbonyl)amino]benzoates and Their Quaternary Ammonium Salts
Source: Molecules. 2017 Nov 23;22(12):2048. doi: 10.3390/molecules22122048 (PMC6149889; doi:10.3390/molecules22122048)
Supplement: Supplementary file 1 [file molecules-22-02048-s001.pdf]

Supplementary material

Synthesis, Analysis, Cholinesterase-Inhibiting Activity and Molecular Modelling Studies of 3-(Dialkylamino)-2-hydroxypropyl 4-[(Alkoxy-carbonyl)amino]benzoates and Their Quaternary Ammonium Salts

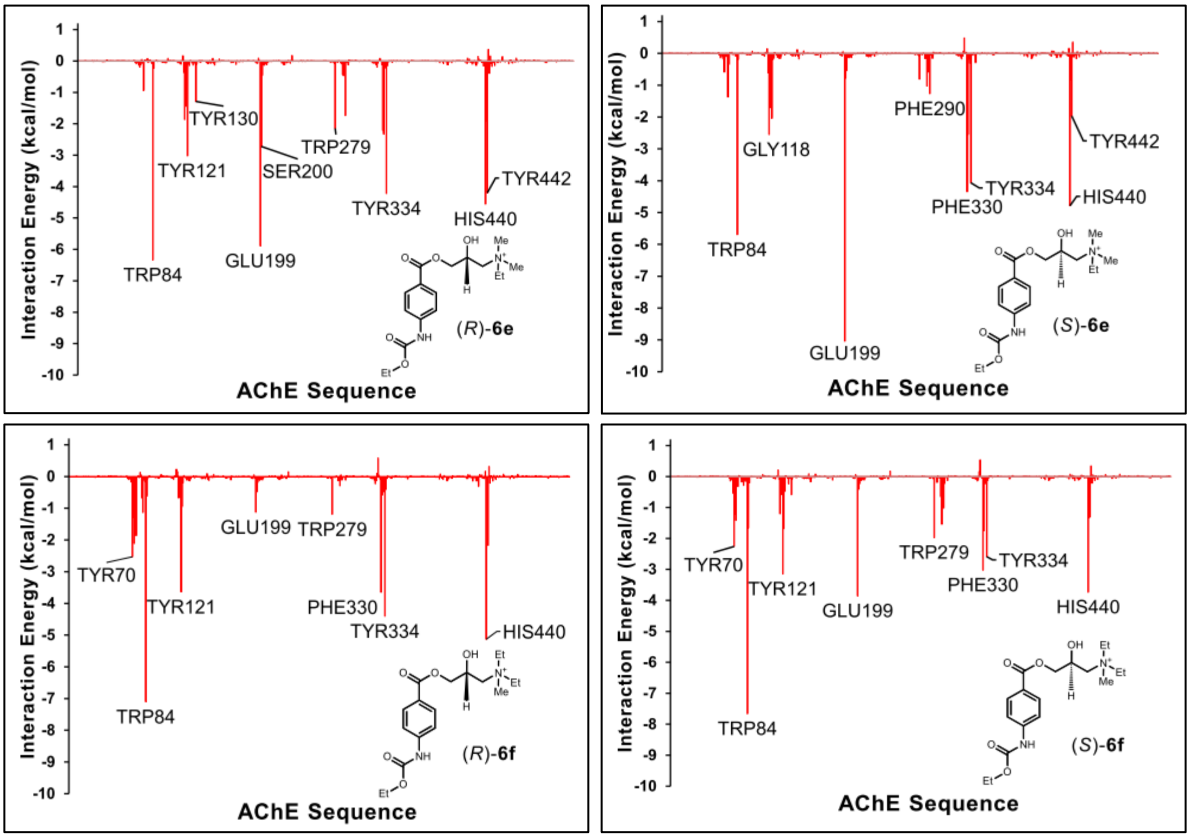

Figure S1. Histograms of interaction energies partitioned with respect to the AChE, amino acids in complex with (*R*)-**6e**, (*S*)-**6e,** (*R*)-**6f** and (*S*)-**6f**.

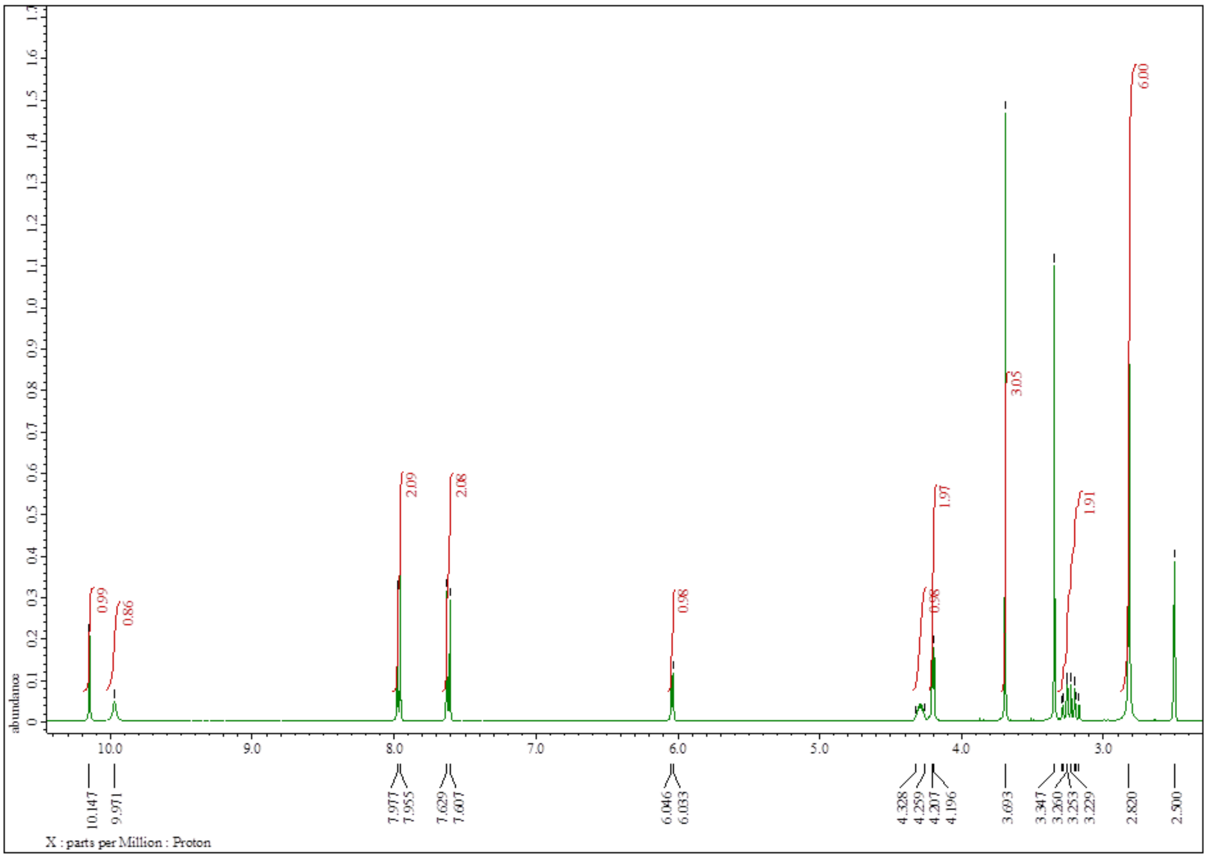

Figure S2. ^{1}H-NMR spectrum of **5a**

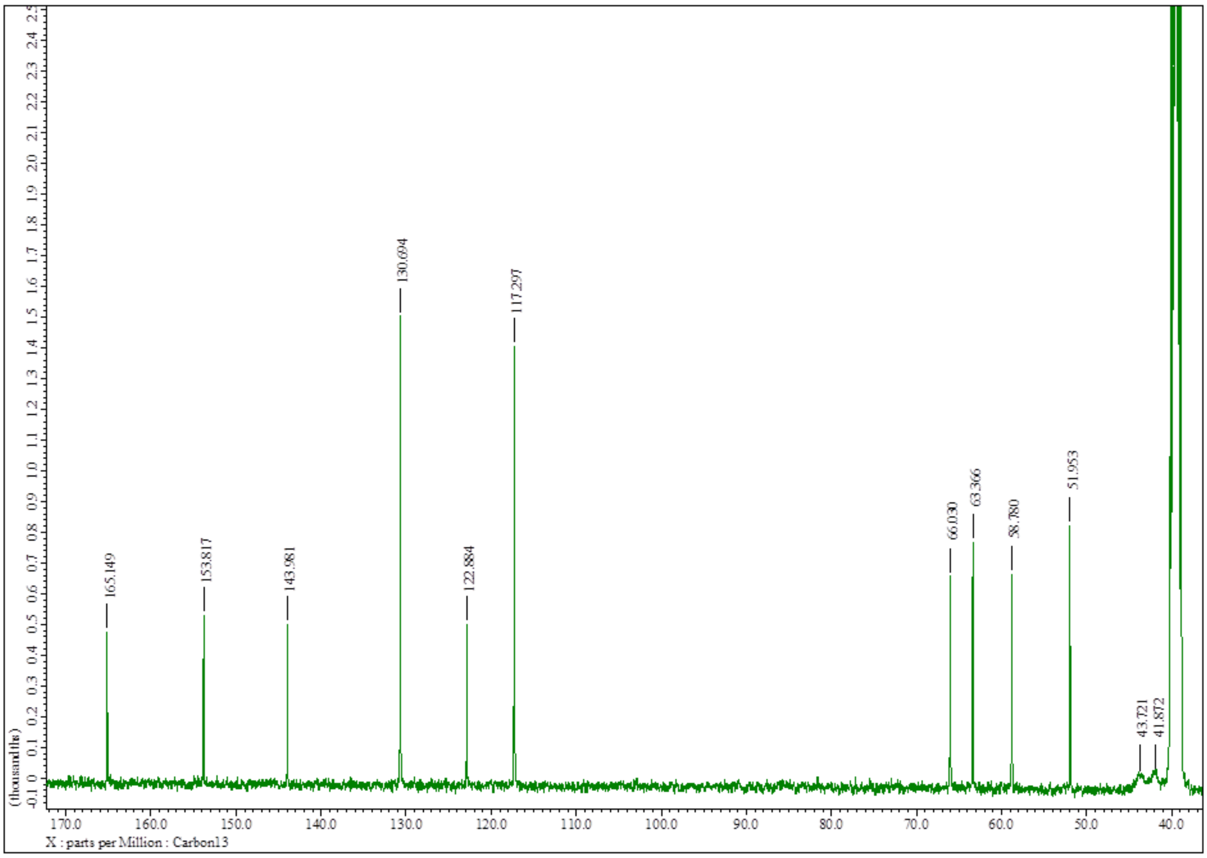

Figure S3. ^{13}C-NMR spectrum of **5a**

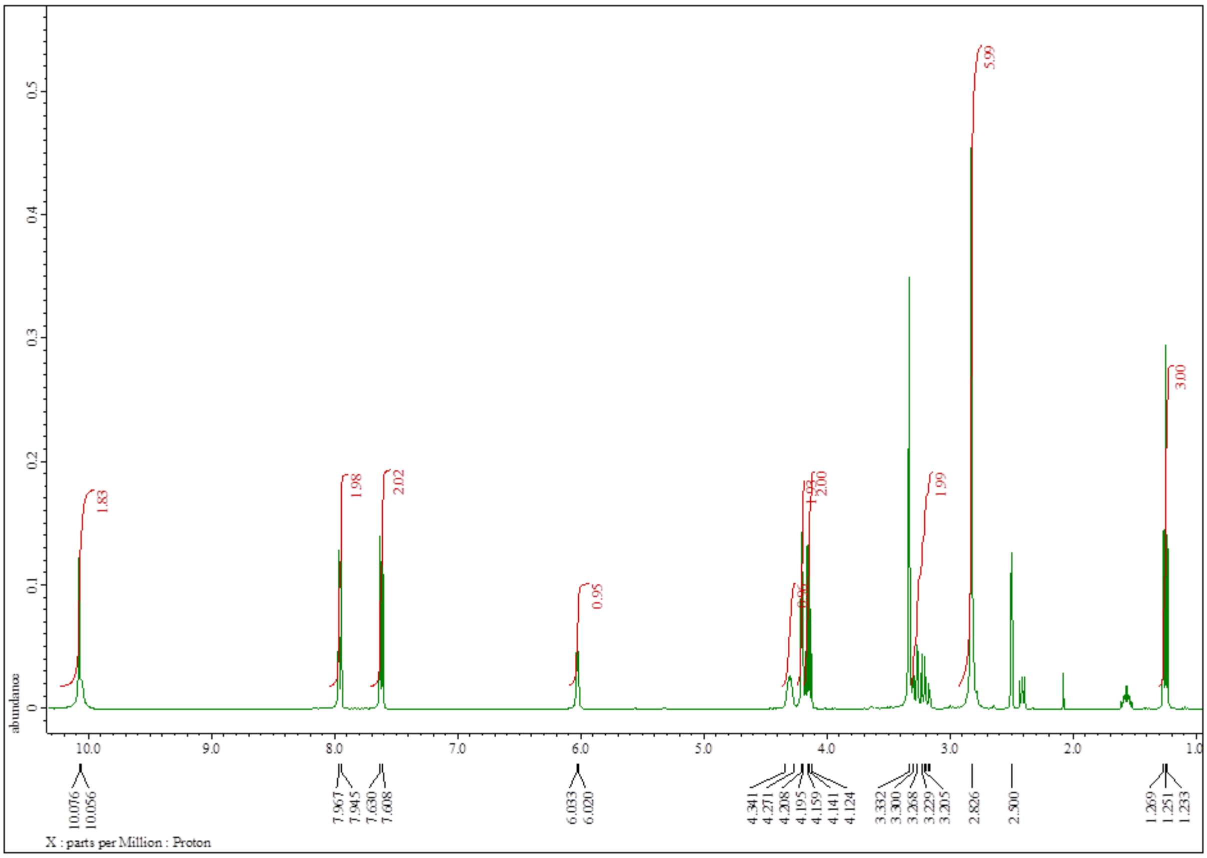

Figure S4. ^{1}H-NMR spectrum of **5b**

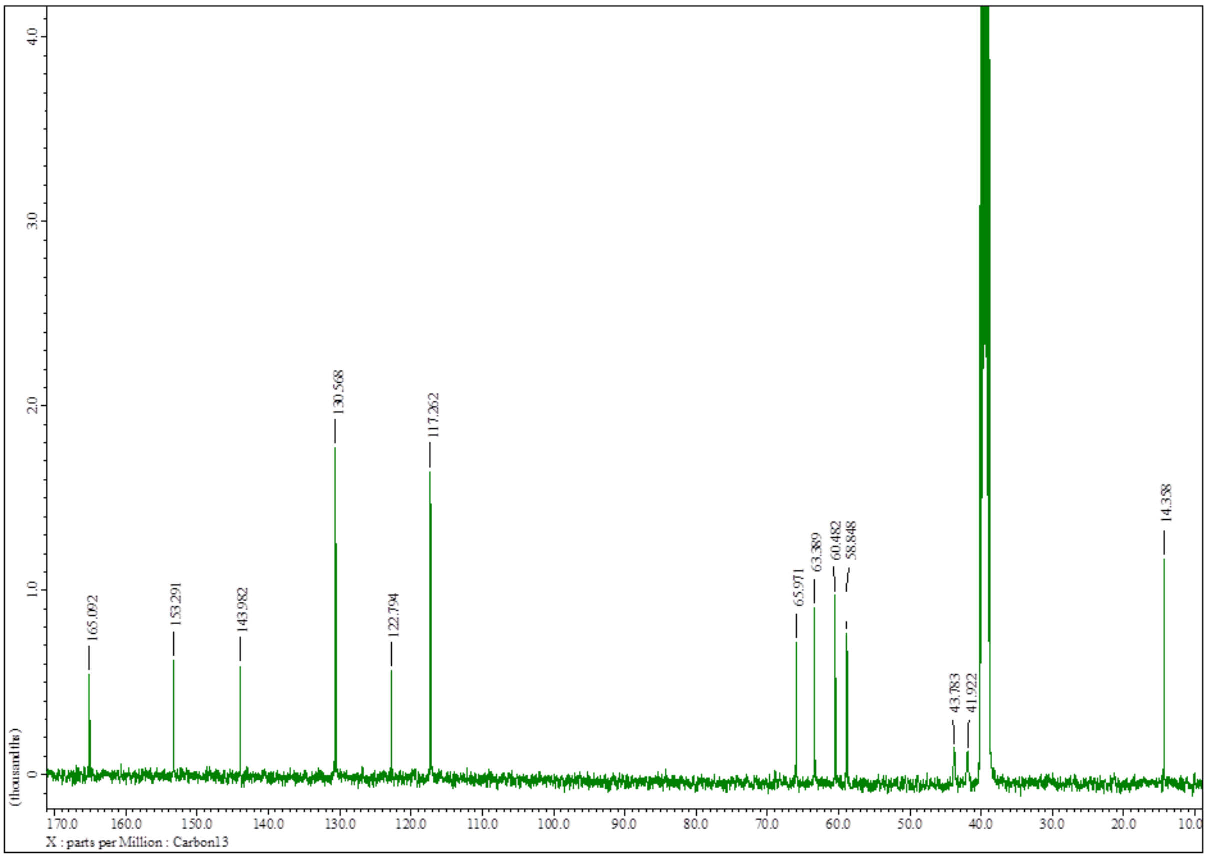

Figure S5. ^{13}C-NMR spectrum of **5b**

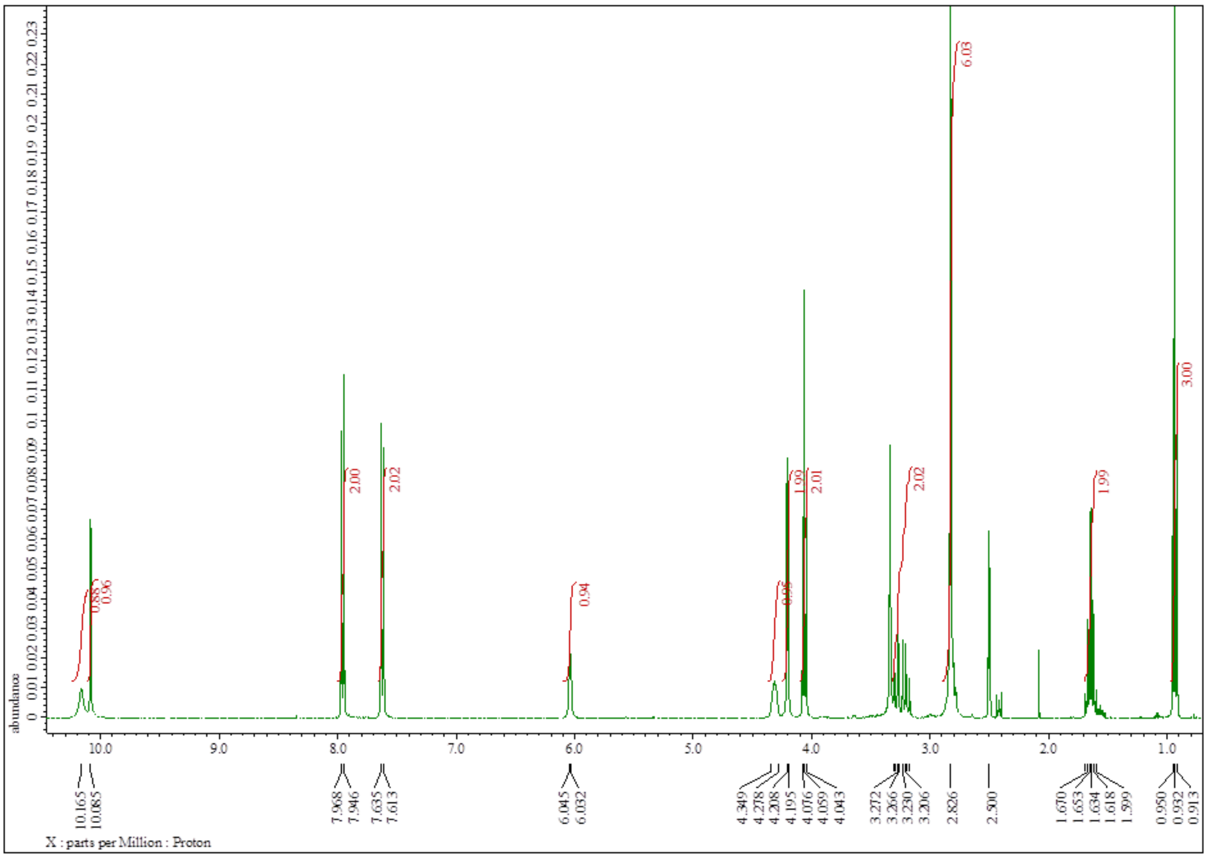

Figure S6. ^{1}H-NMR spectrum of **5c**

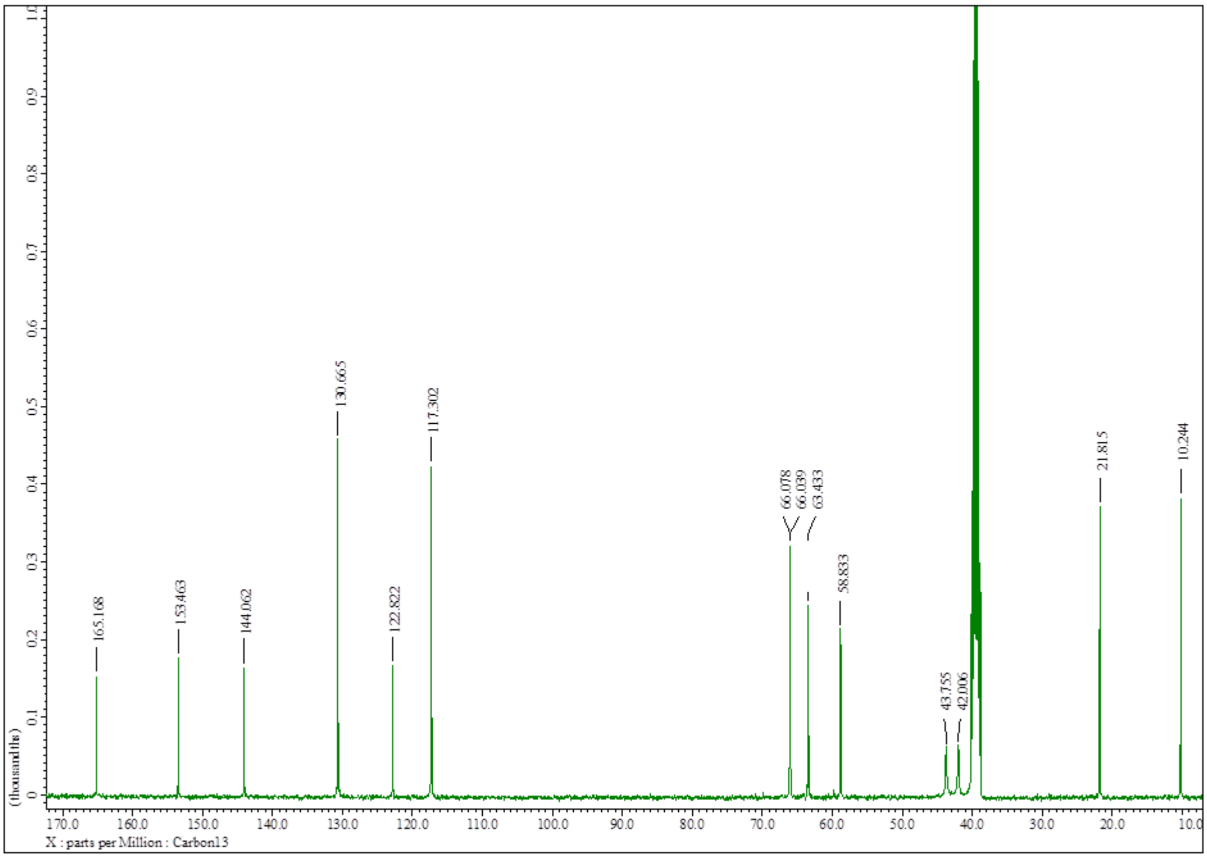

Figure S7. ^{13}C-NMR spectrum of **5c**

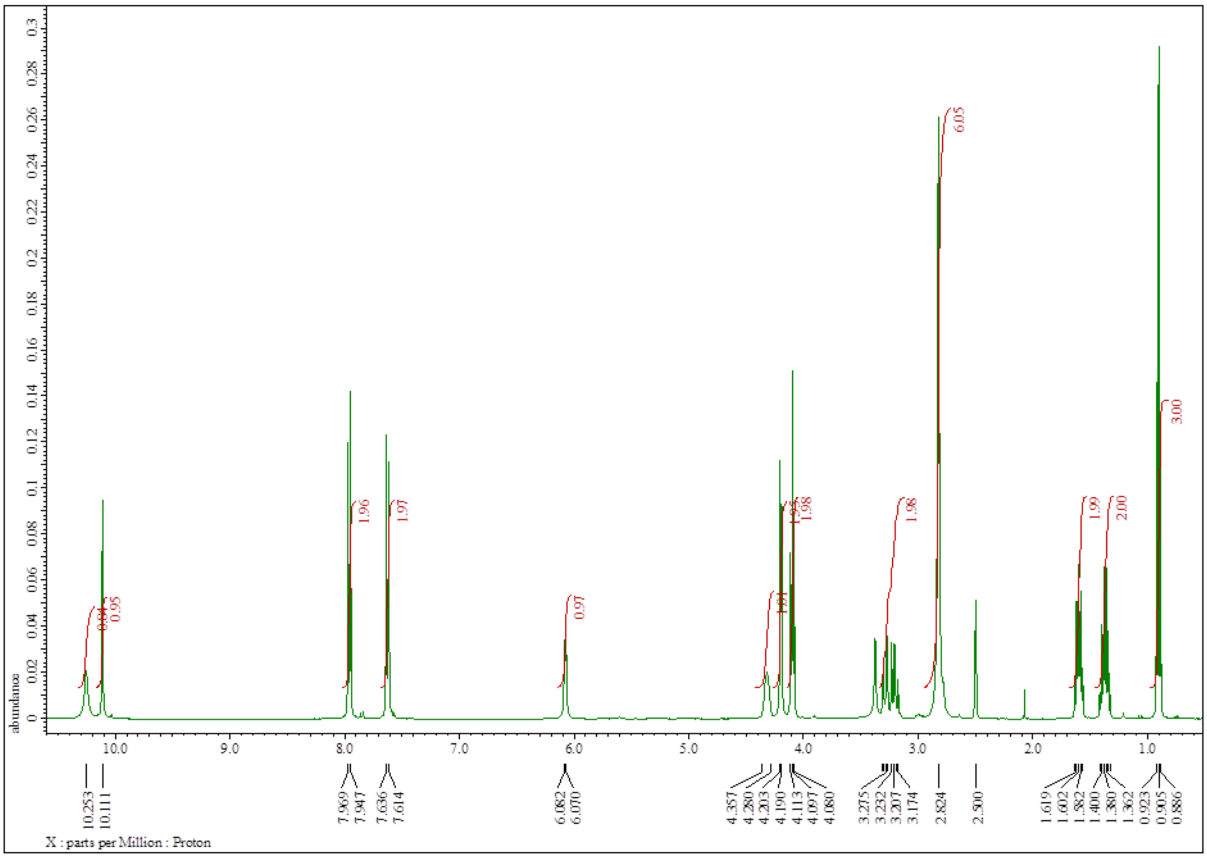

Figure S8. ^{1}H-NMR spectrum of **5d**

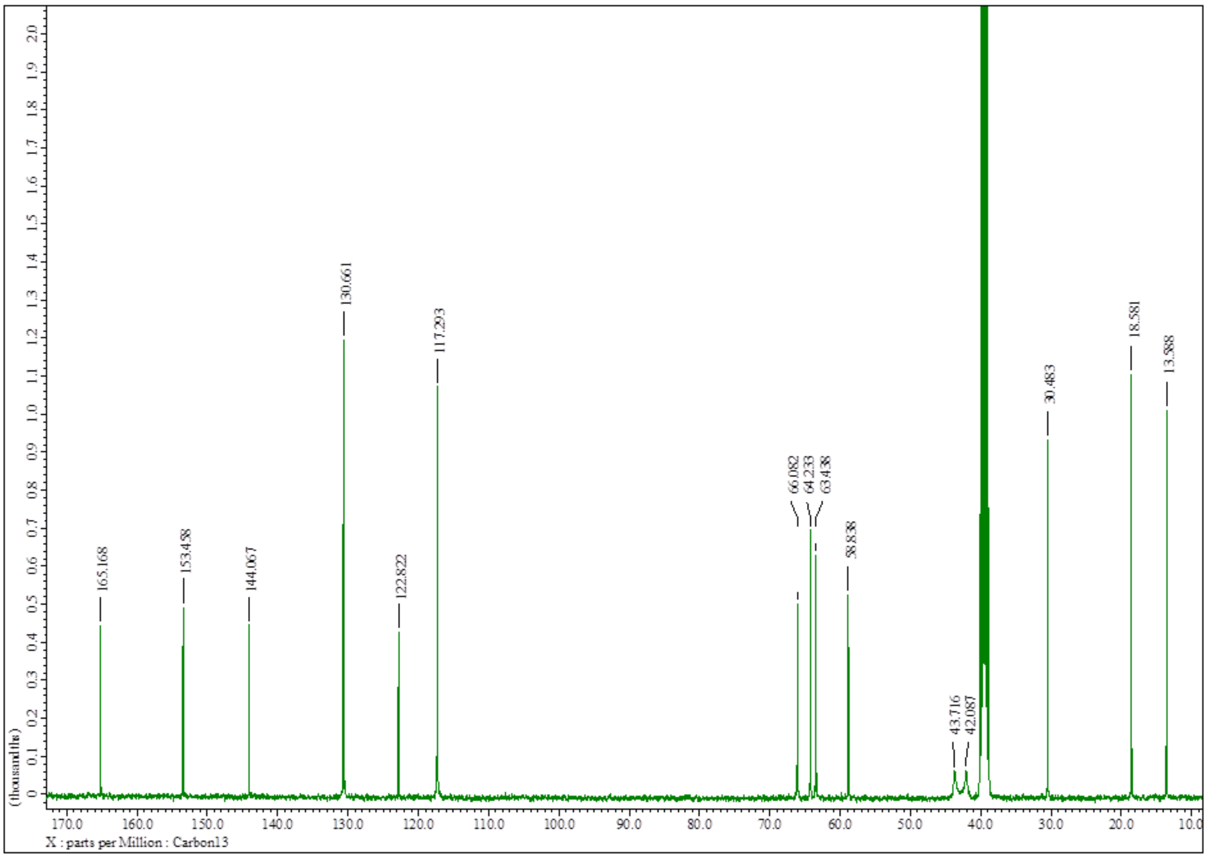

Figure S9. ^{13}C-NMR spectrum of **5d**

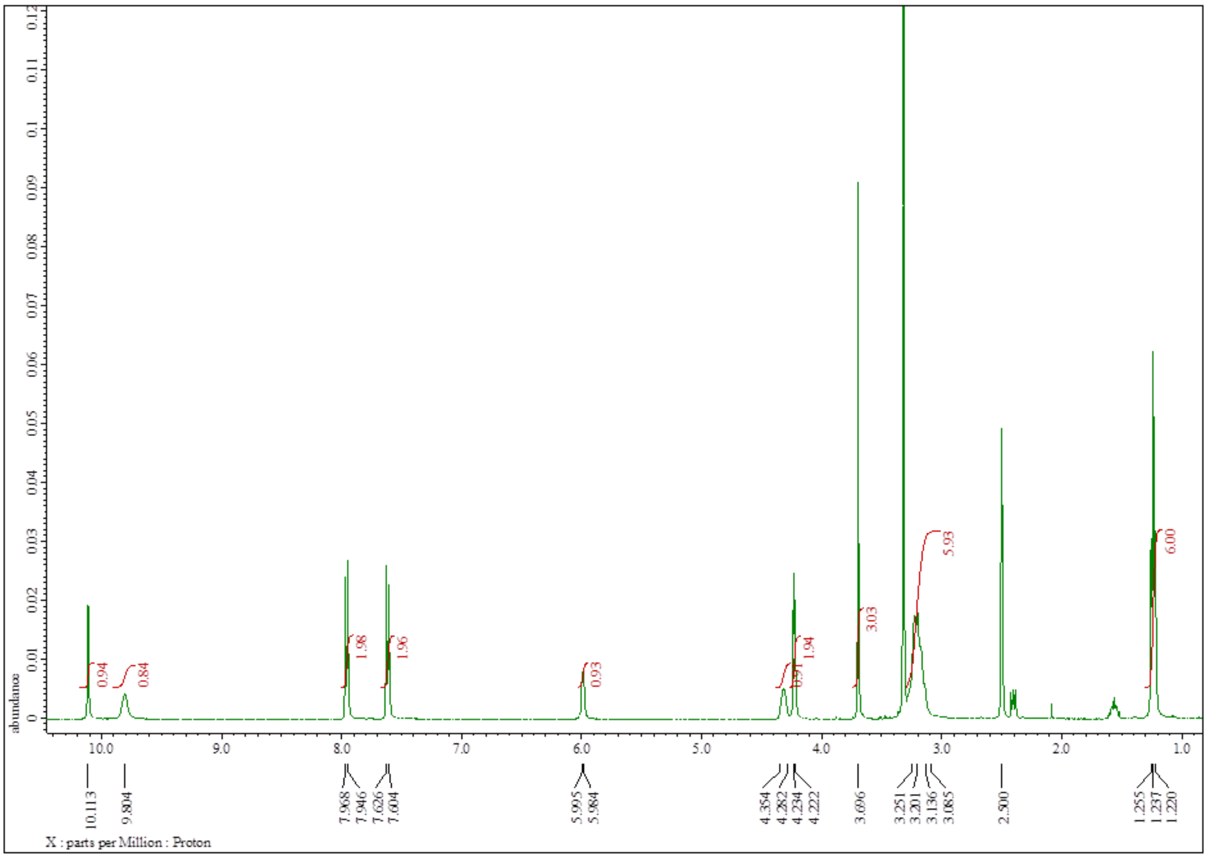

Figure S10. ^{1}H-NMR spectrum of **5e**

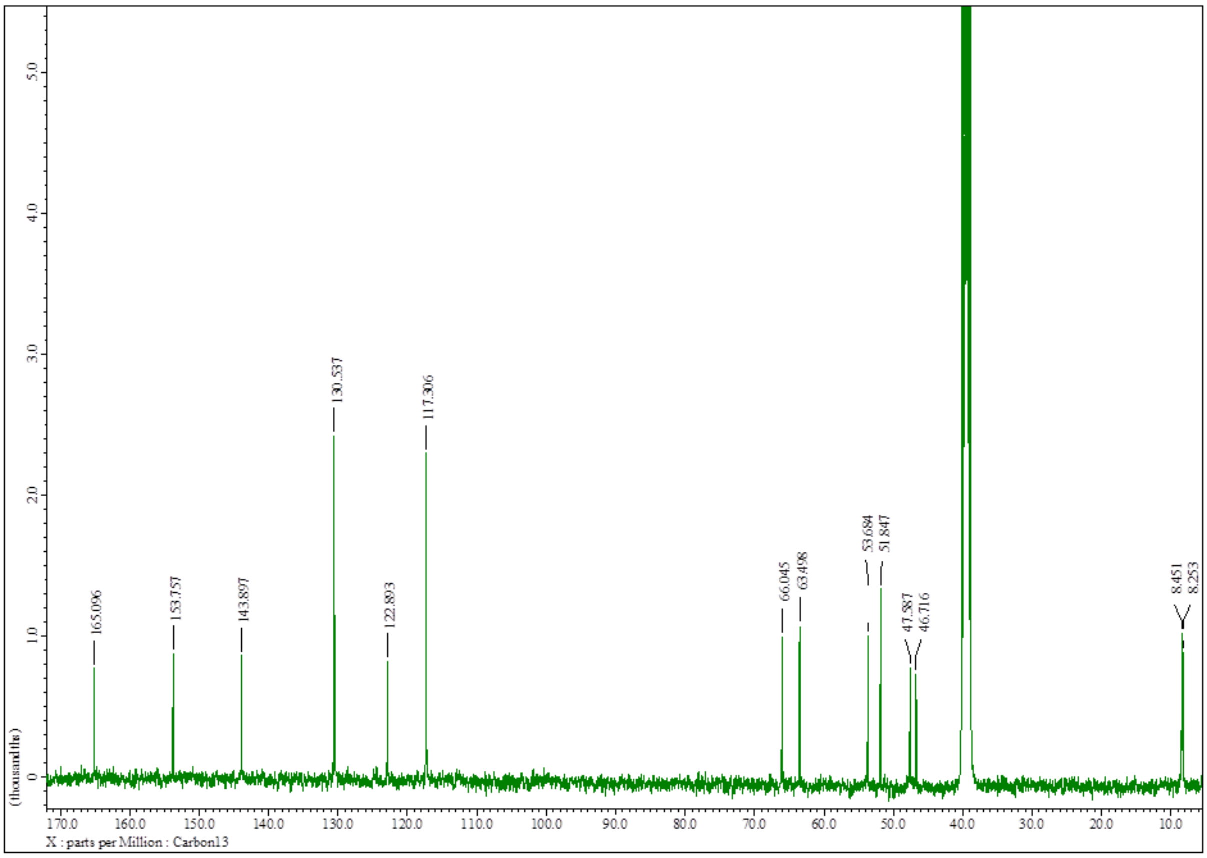

Figure S11. ^{13}C-NMR spectrum of **5e**

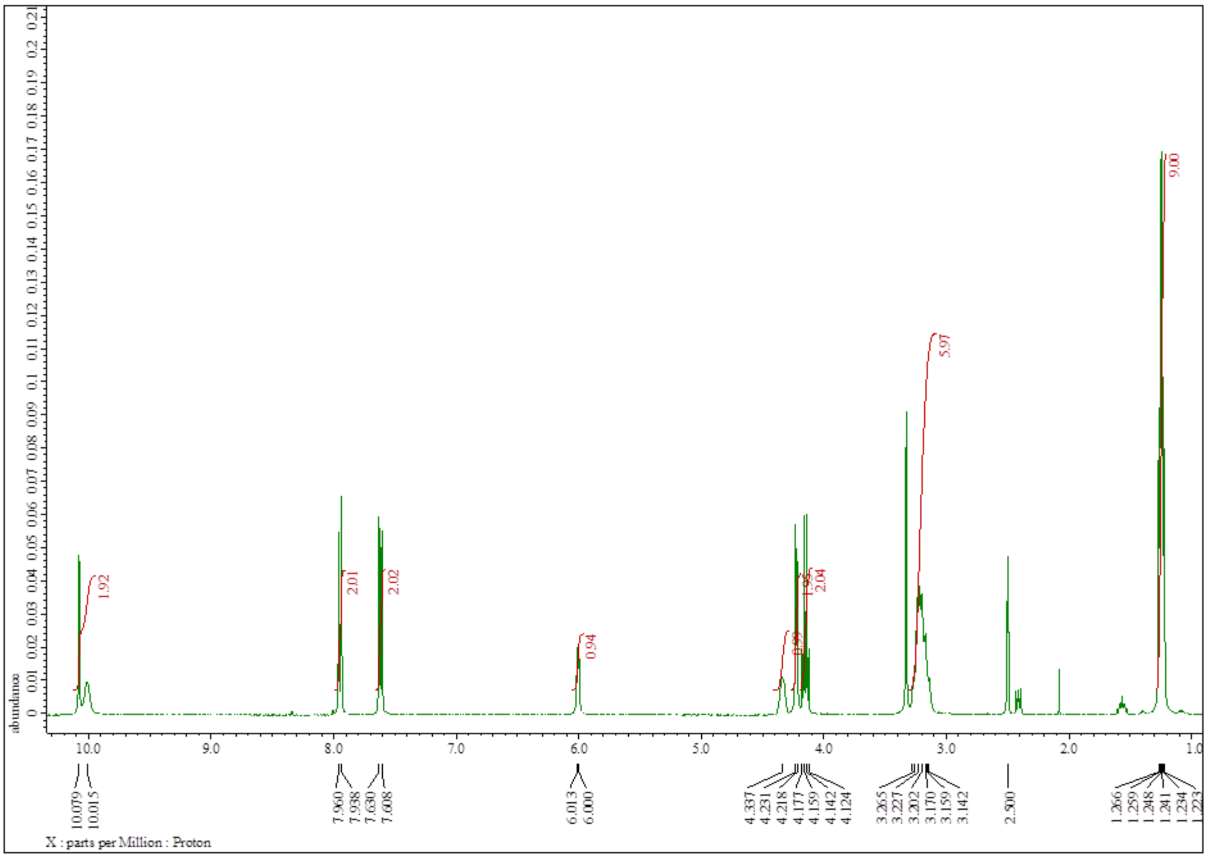

Figure S12. ^{1}H-NMR spectrum of **5f**

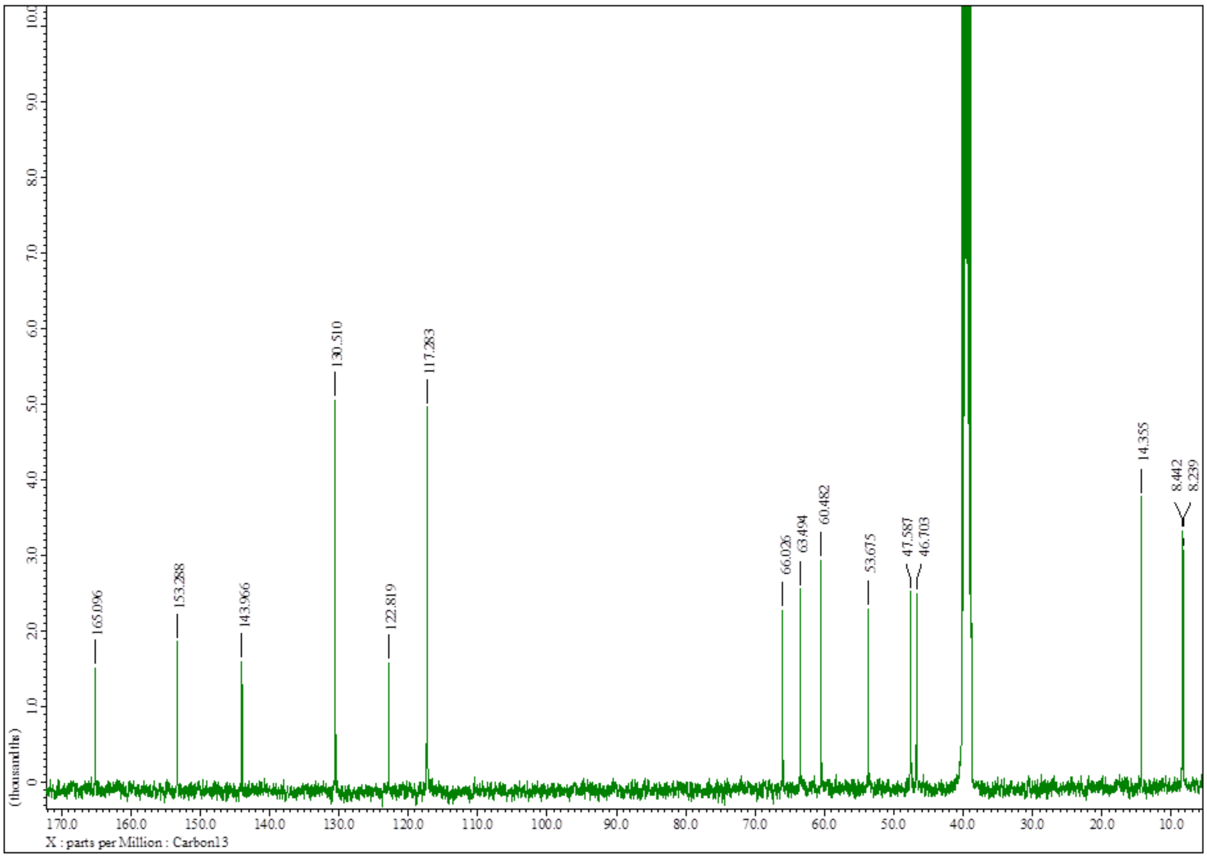

Figure S13. ^{13}C-NMR spectrum of **5f**

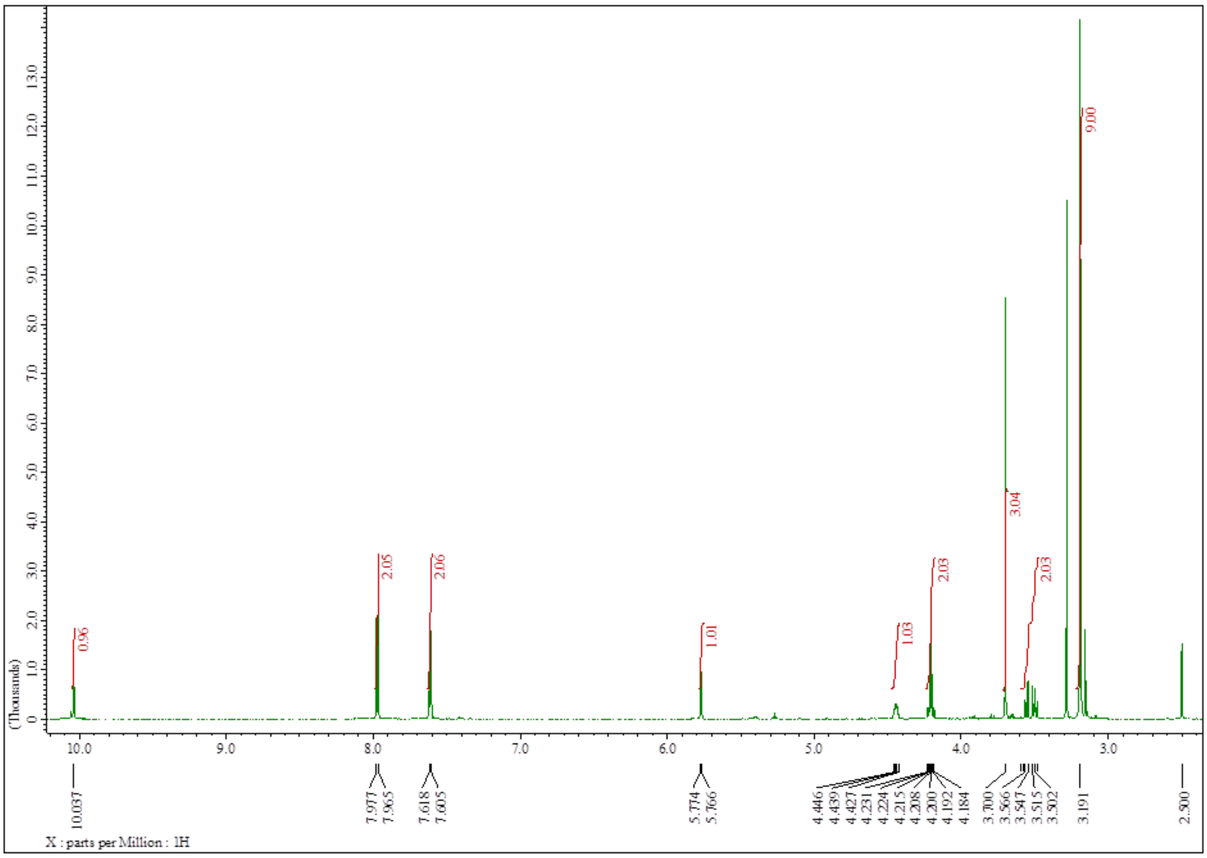

Figure S14. ^{1}H-NMR spectrum of **6a**

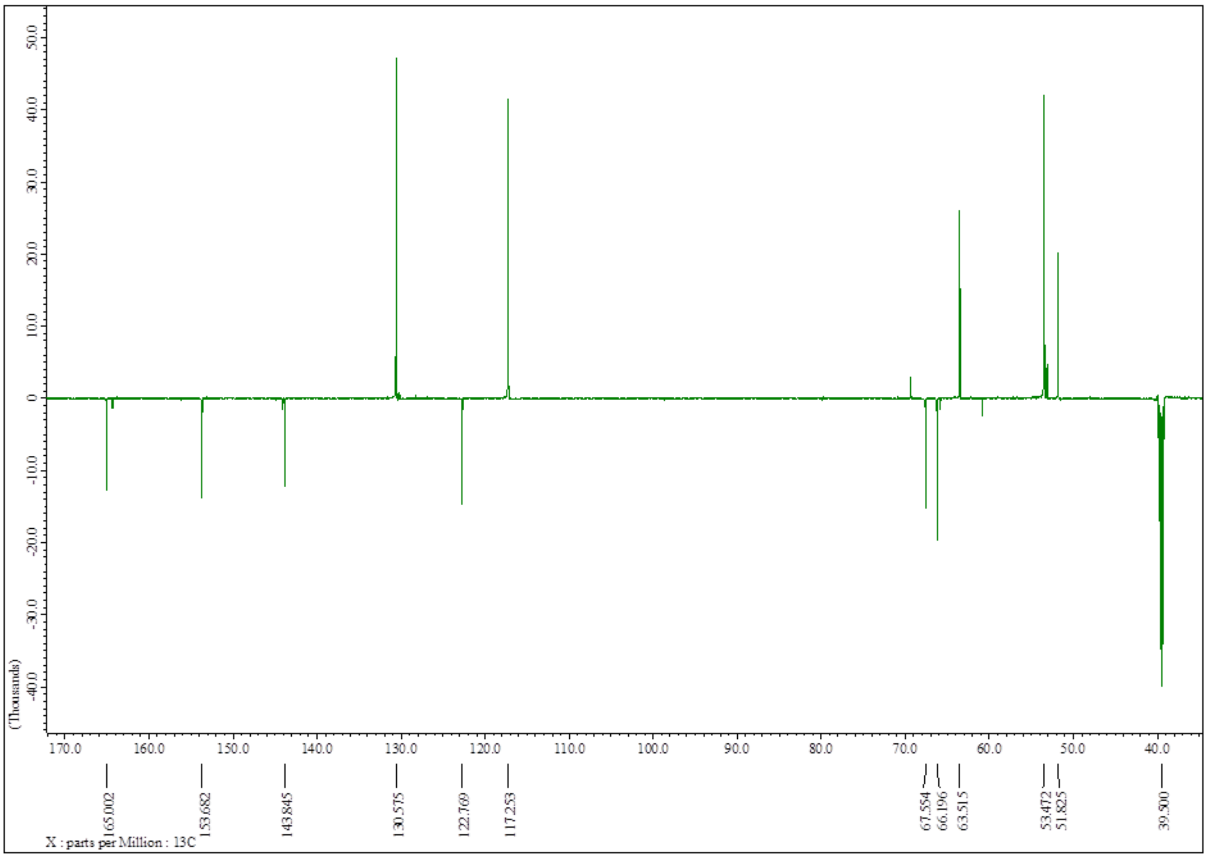

Figure S15. ^{13}C-NMR (APT) spectrum of **6a**

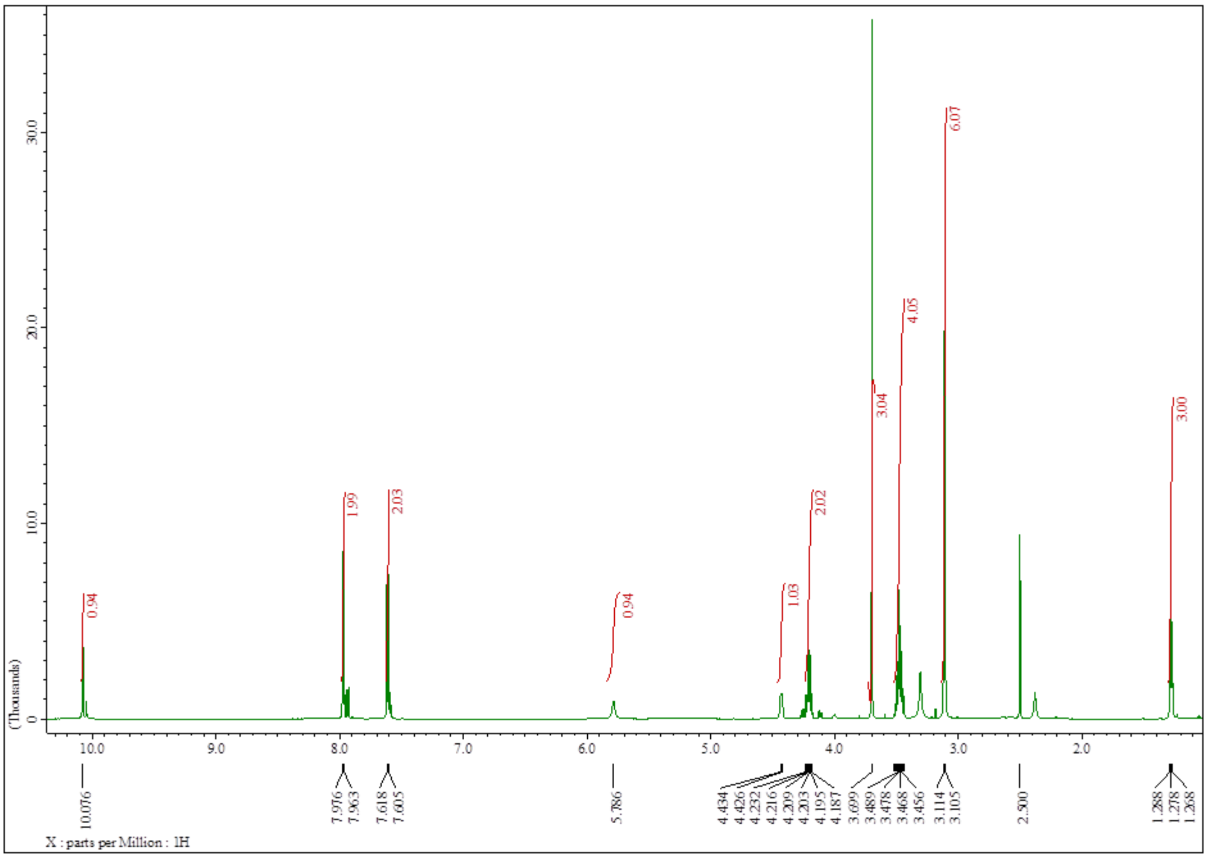

Figure S16. ^{1}H-NMR spectrum of **6b**

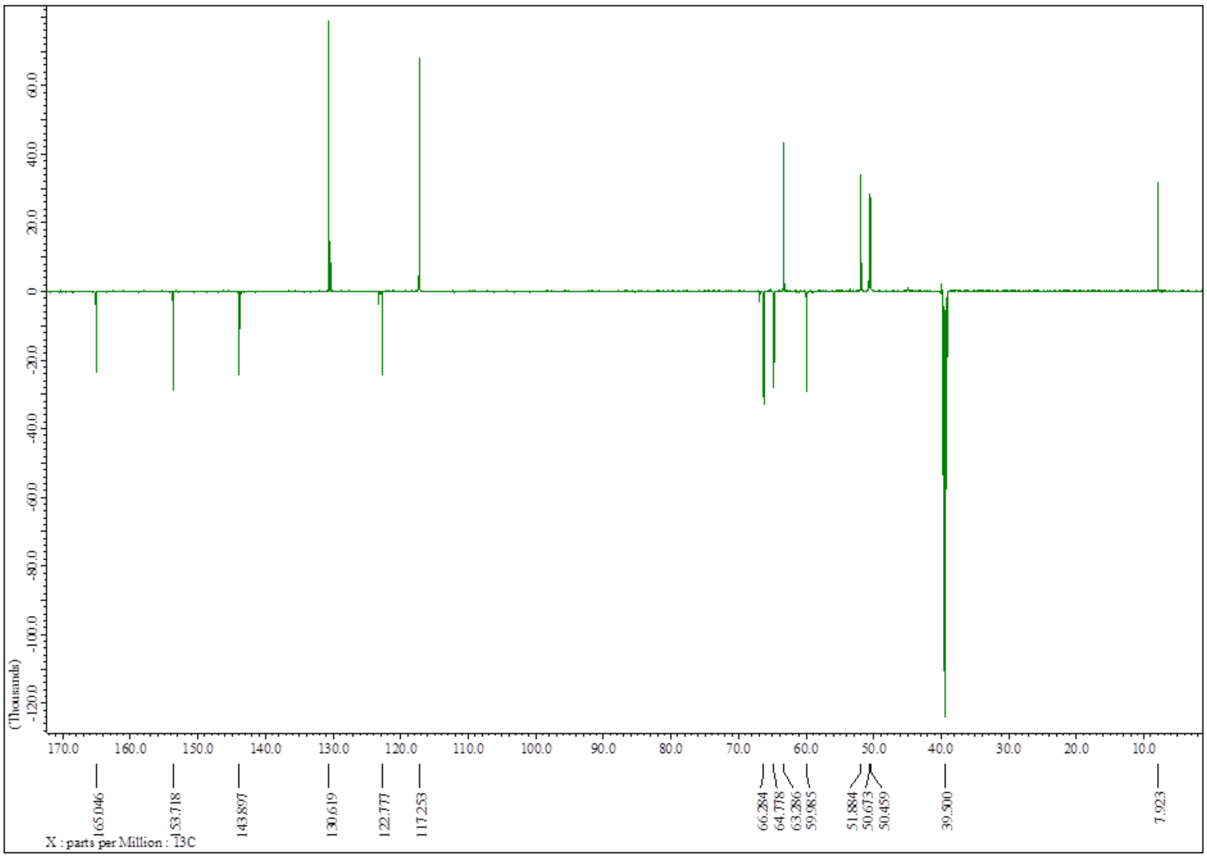

Figure S17. ^{13}C-NMR (APT) spectrum of **6b**

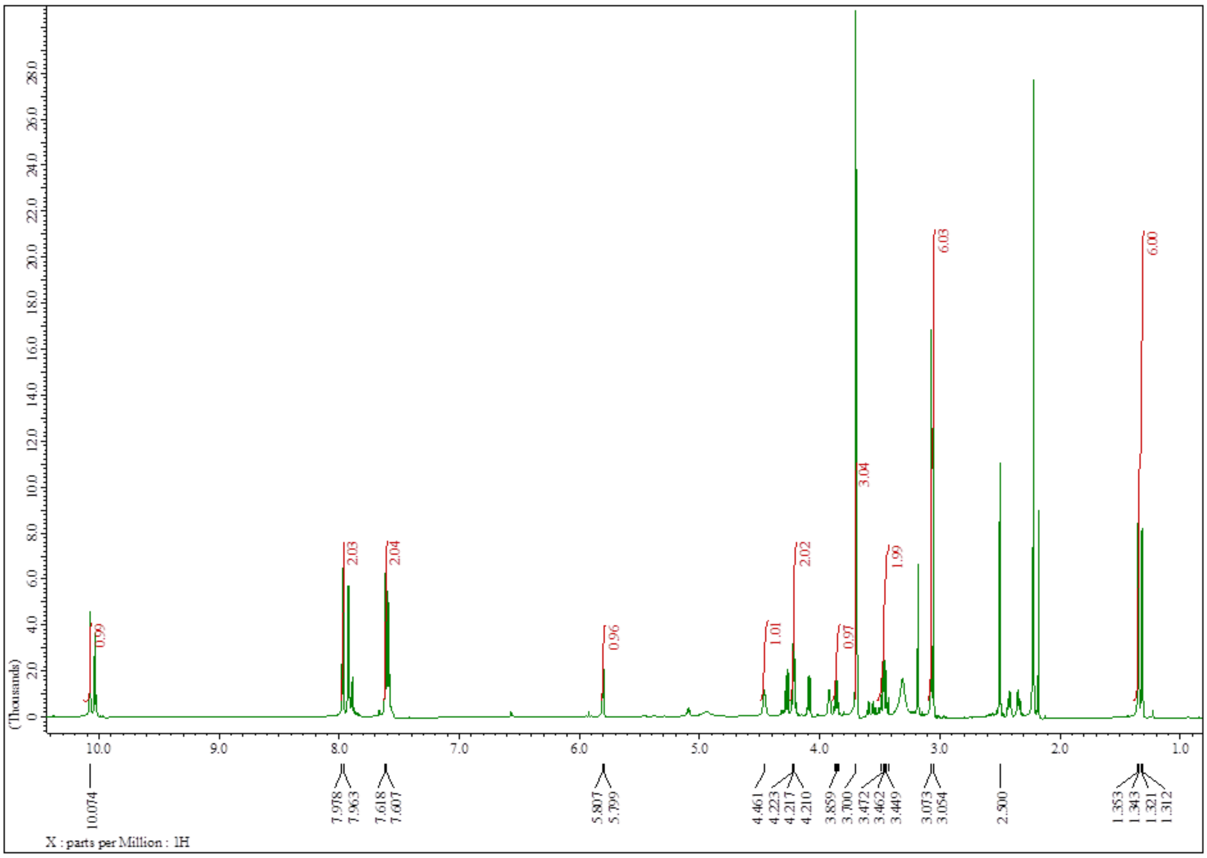

Figure S18. ^{1}H-NMR spectrum of **6c**

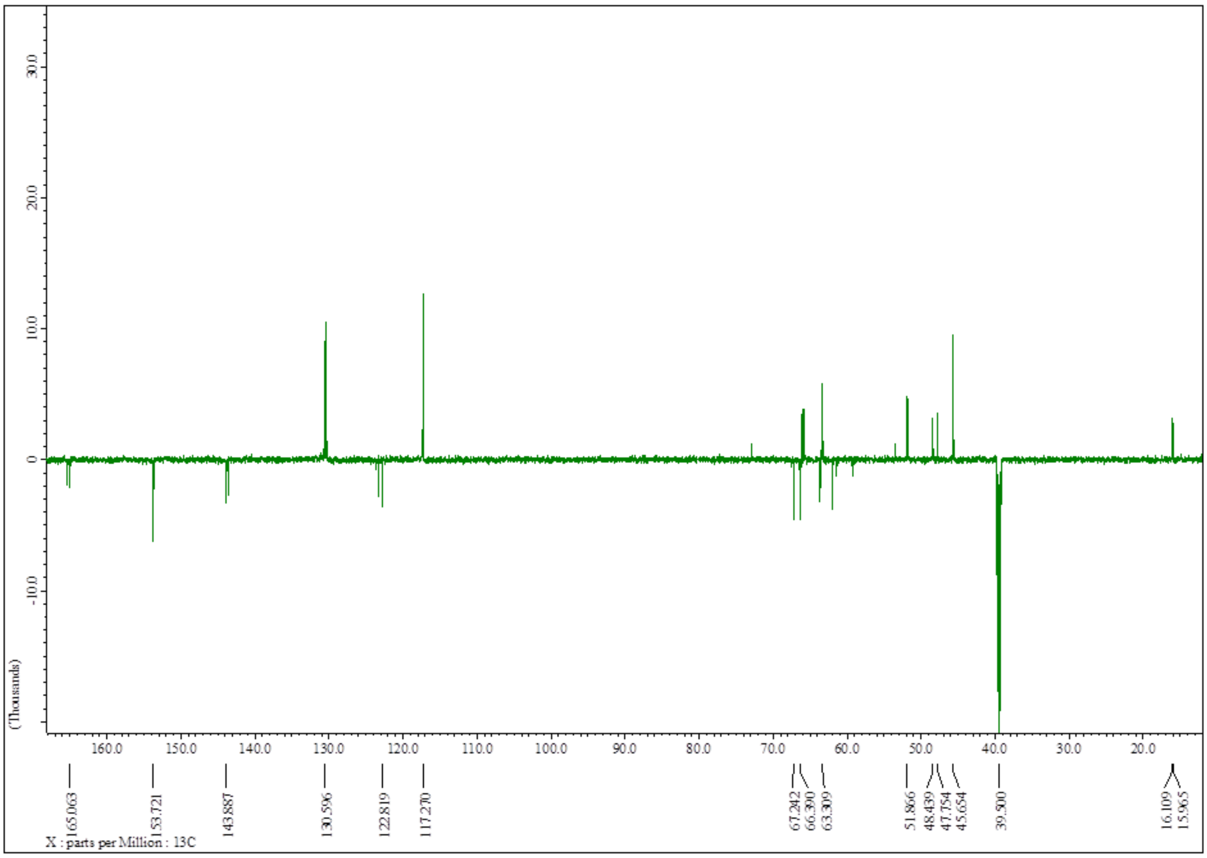

Figure S19. ^{13}C-NMR (APT) spectrum of **6c**

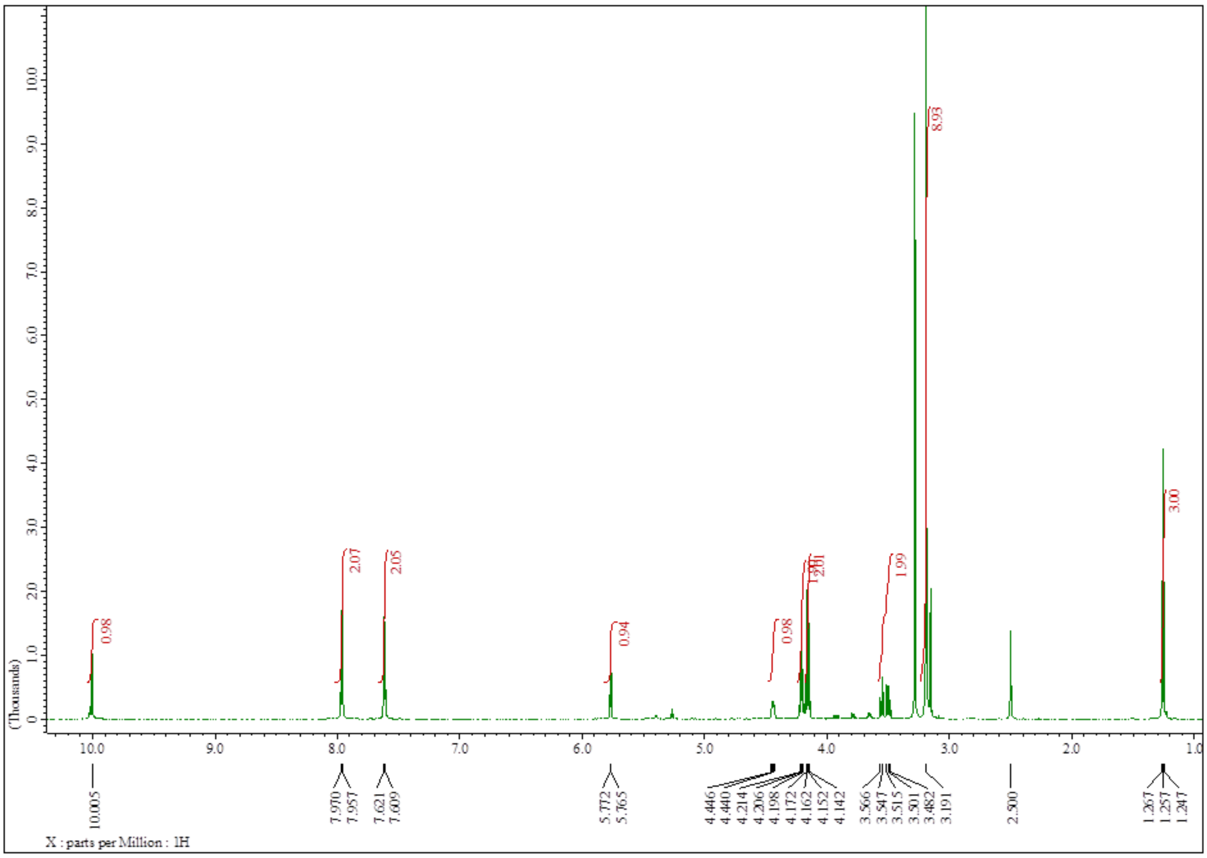

Figure S20. ^{1}H-NMR spectrum of **6d**

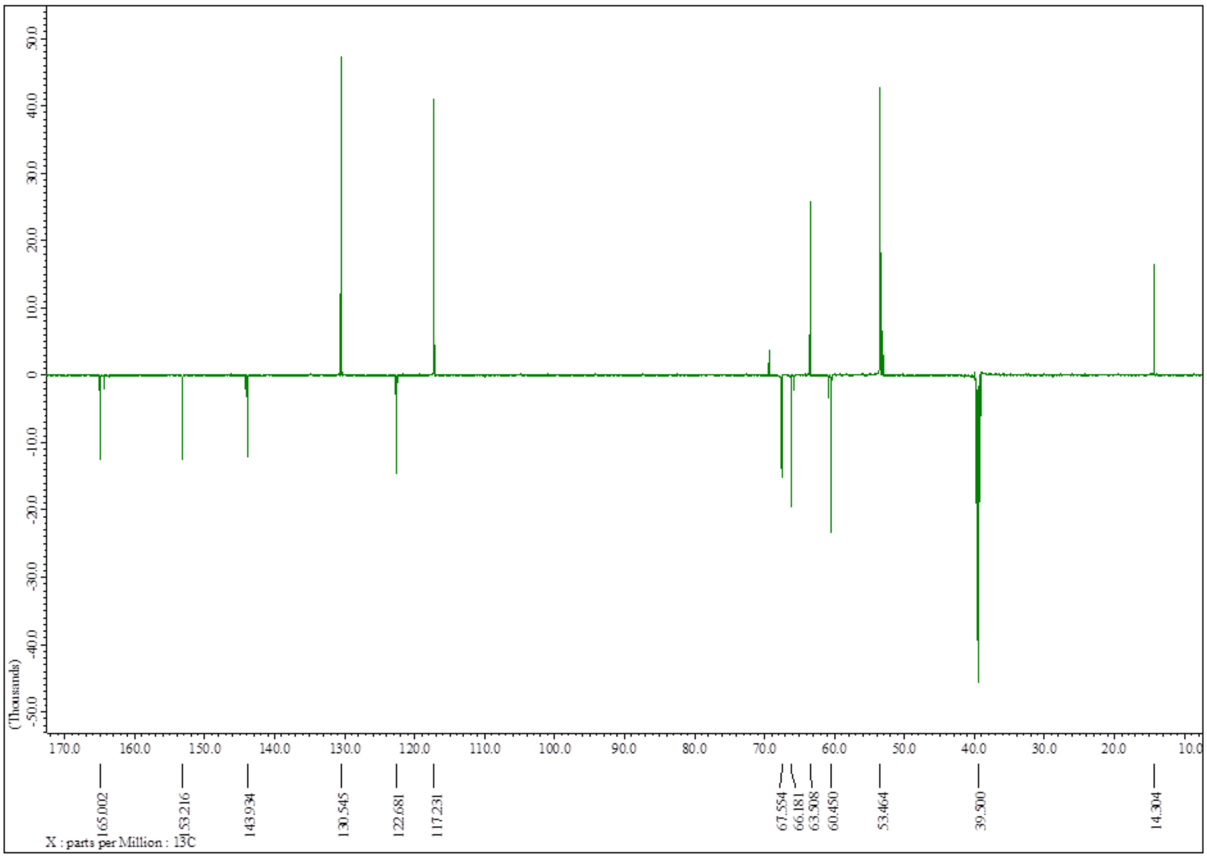

Figure S21. ^{13}C-NMR (APT) spectrum of **6d**

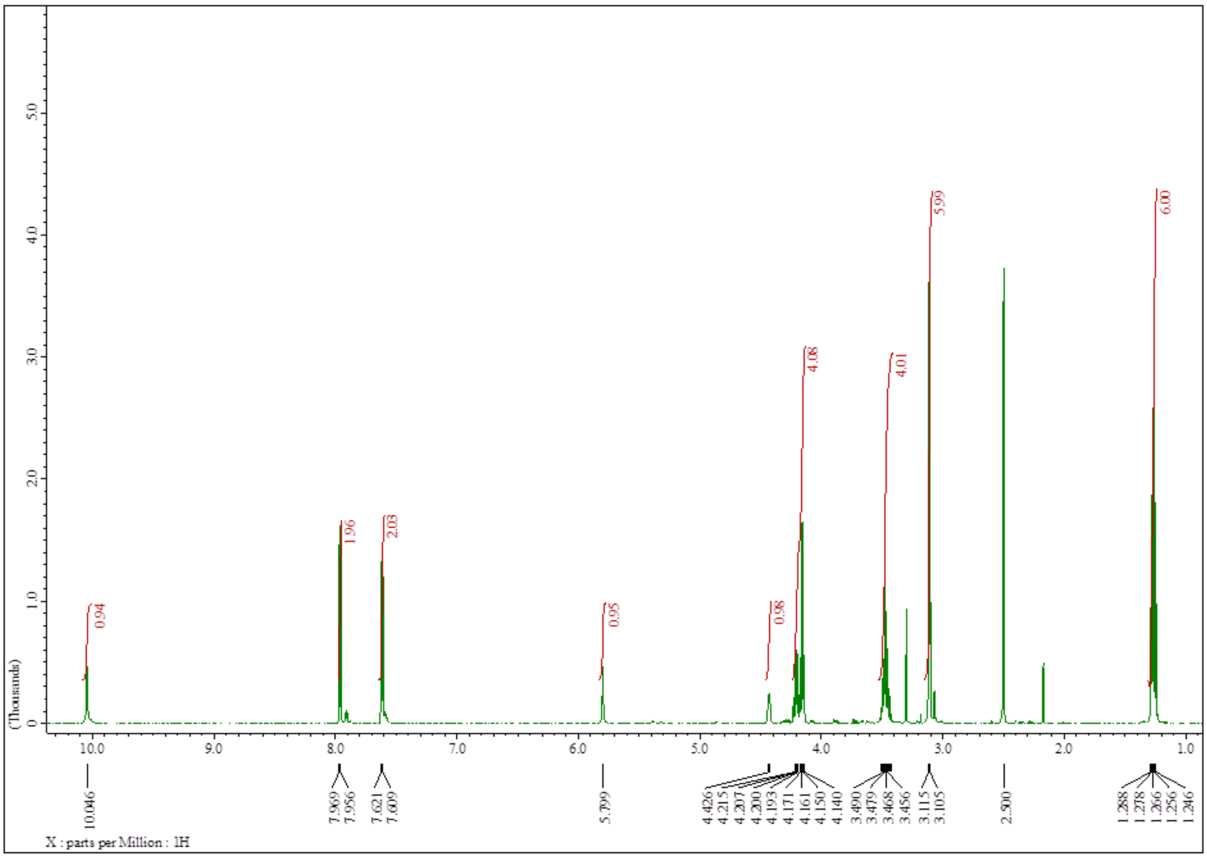

Figure S22. ^{1}H-NMR spectrum of **6e**

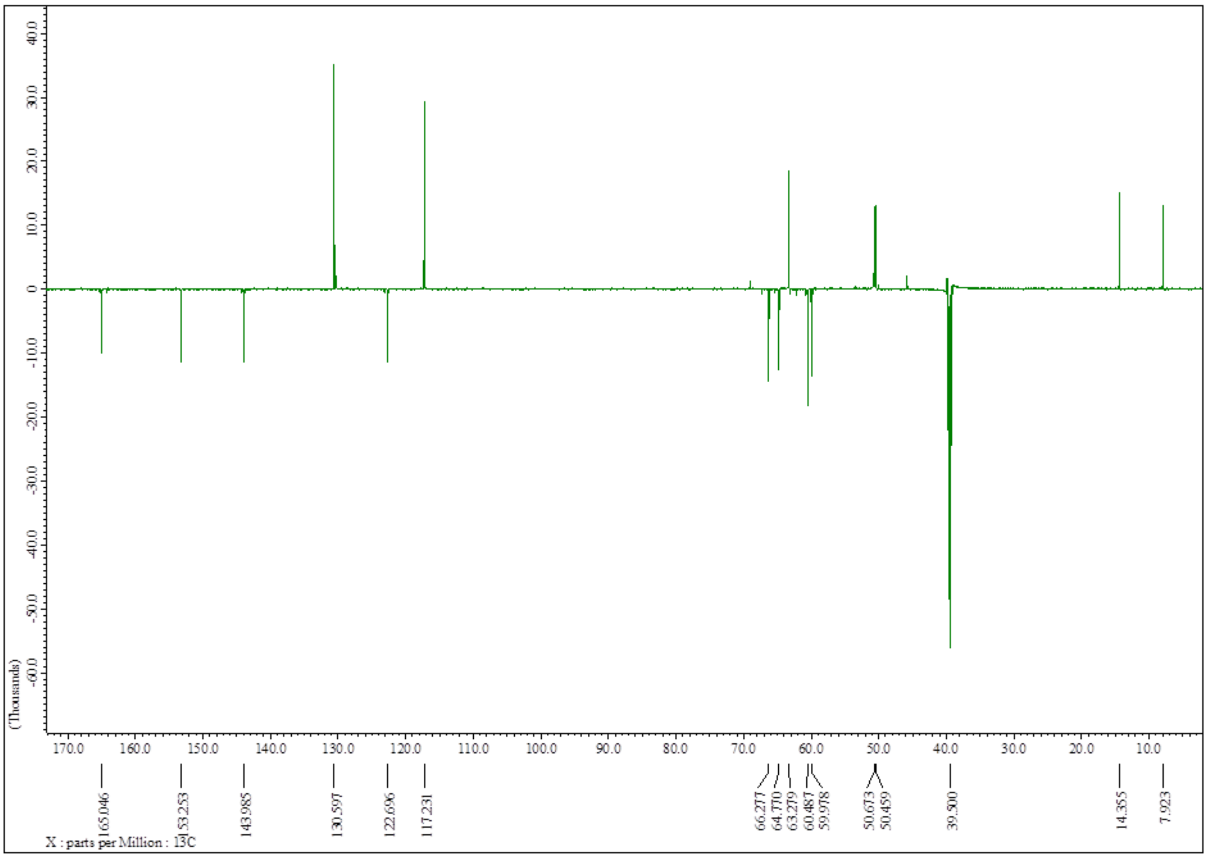

Figure S23. ^{13}C-NMR (APT) spectrum of **6e**

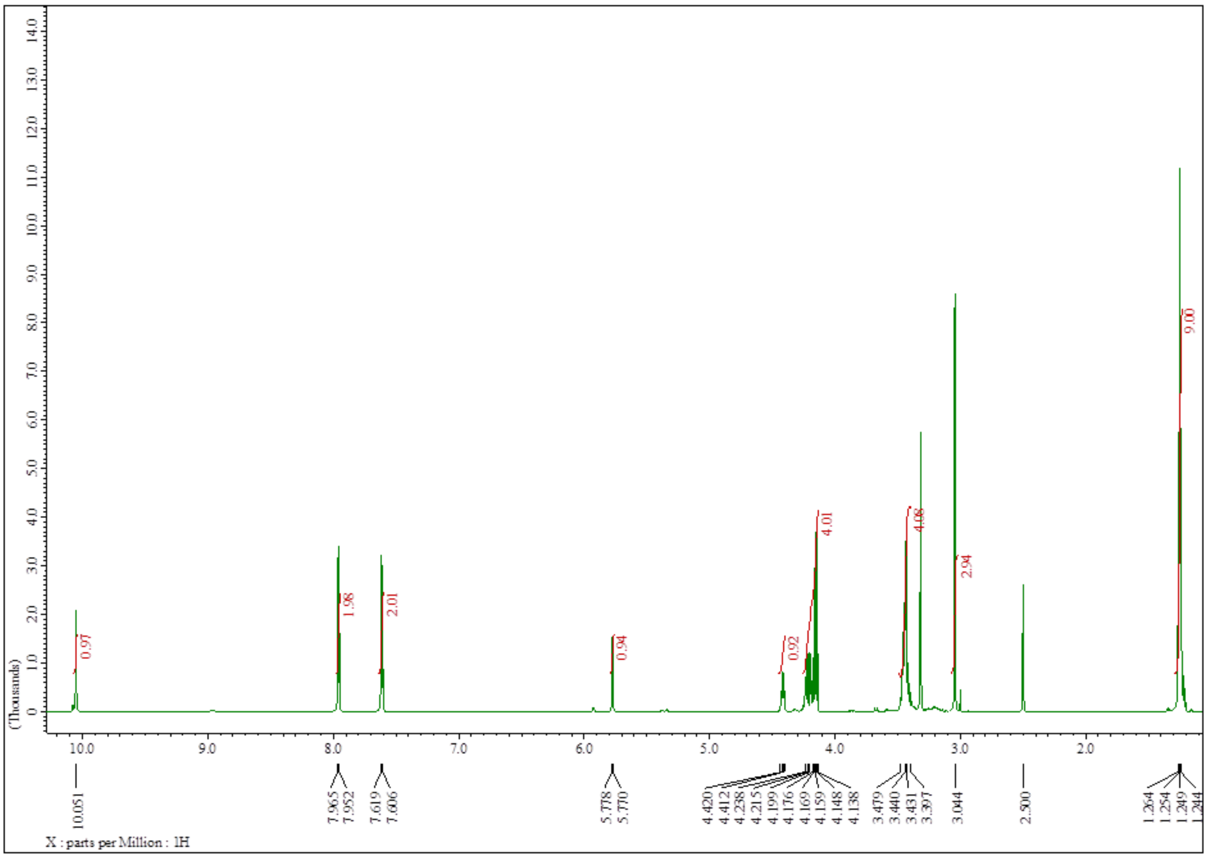

Figure S24. ^{1}H-NMR spectrum of **6f**

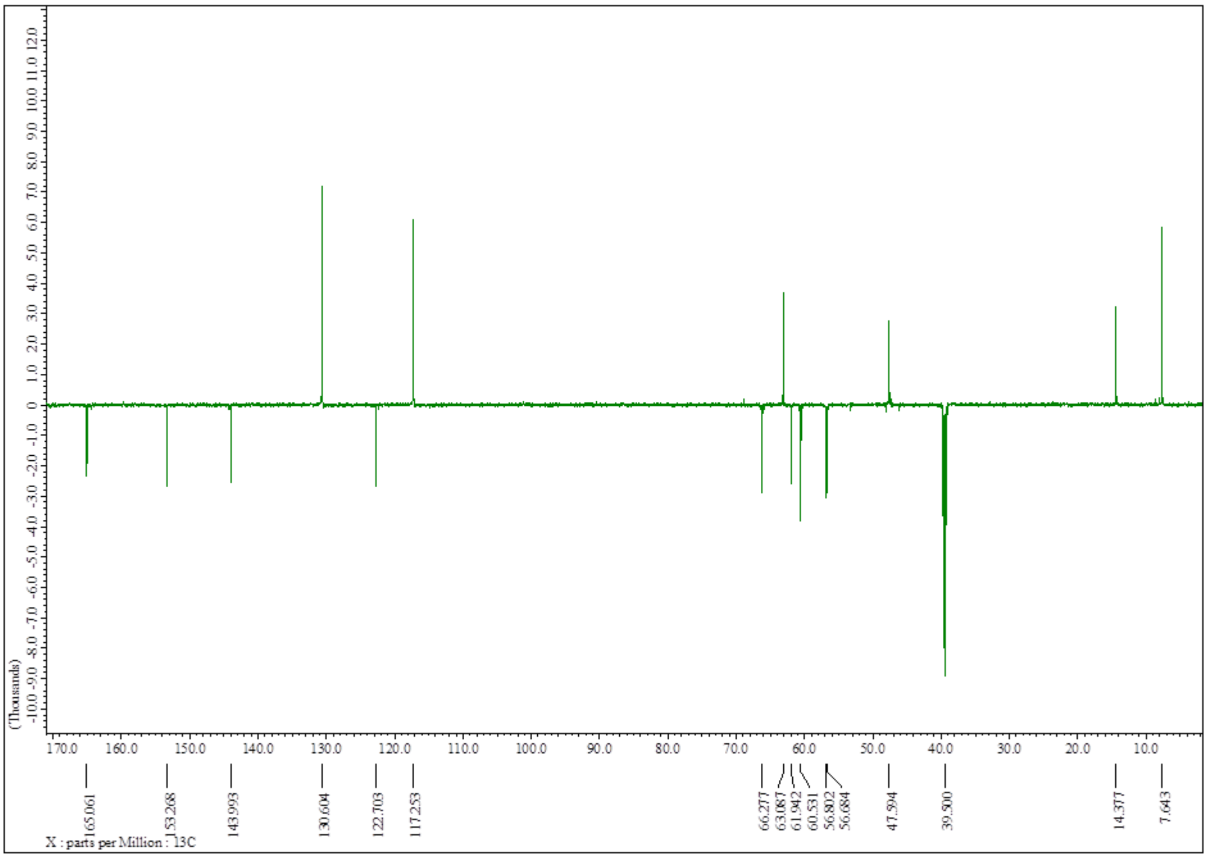

Figure S25. ^{13}C-NMR (APT) spectrum of **6f**

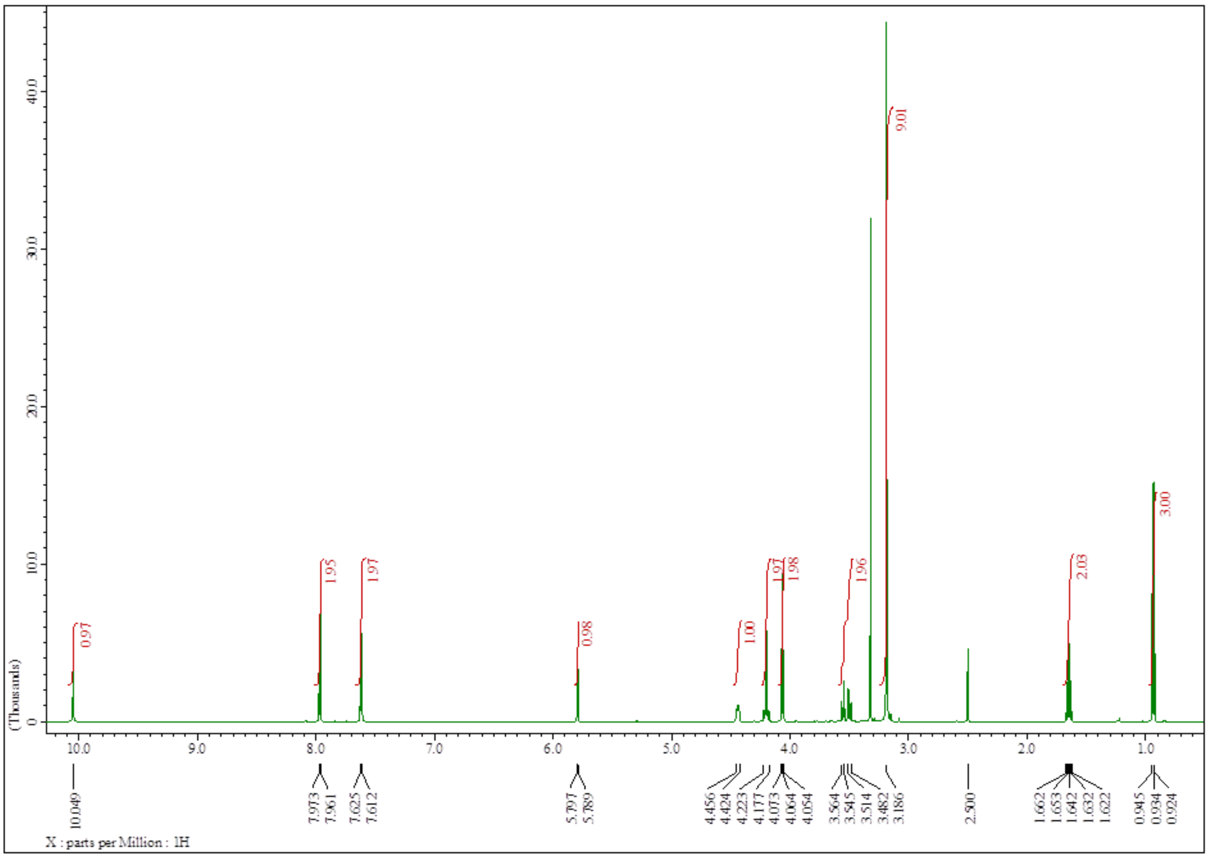

Figure S26. ^{1}H-NMR spectrum of **6g**

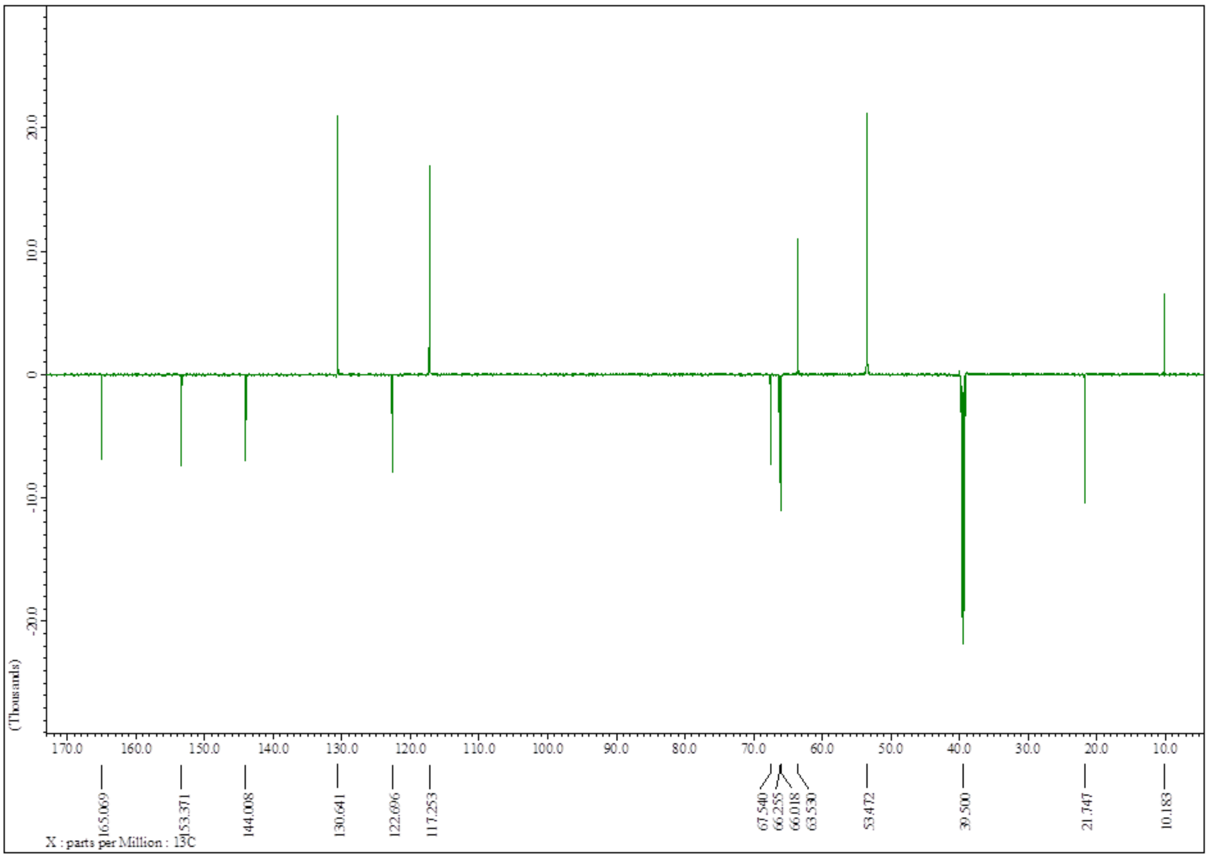

Figure S27. ^{13}C-NMR (APT) spectrum of **6g**

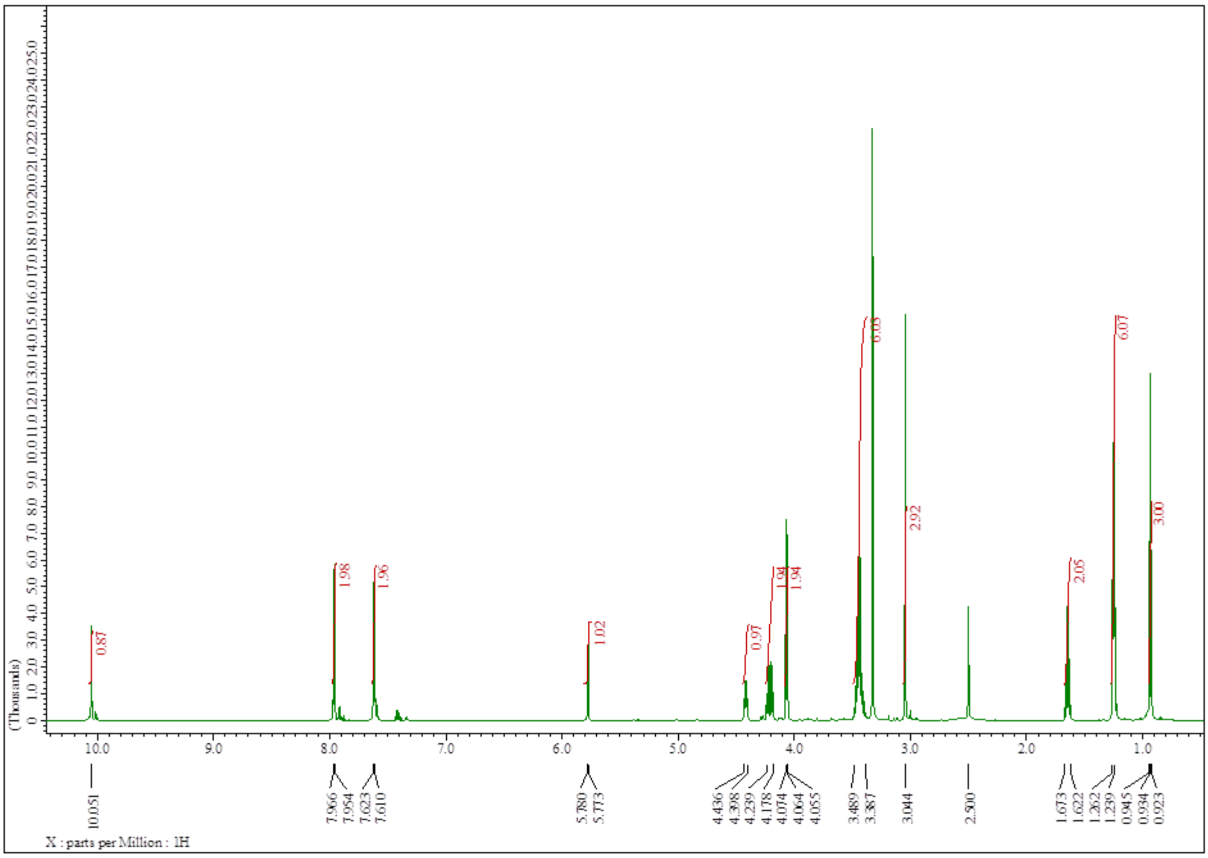

Figure S28. ^{1}H-NMR spectrum of **6h**

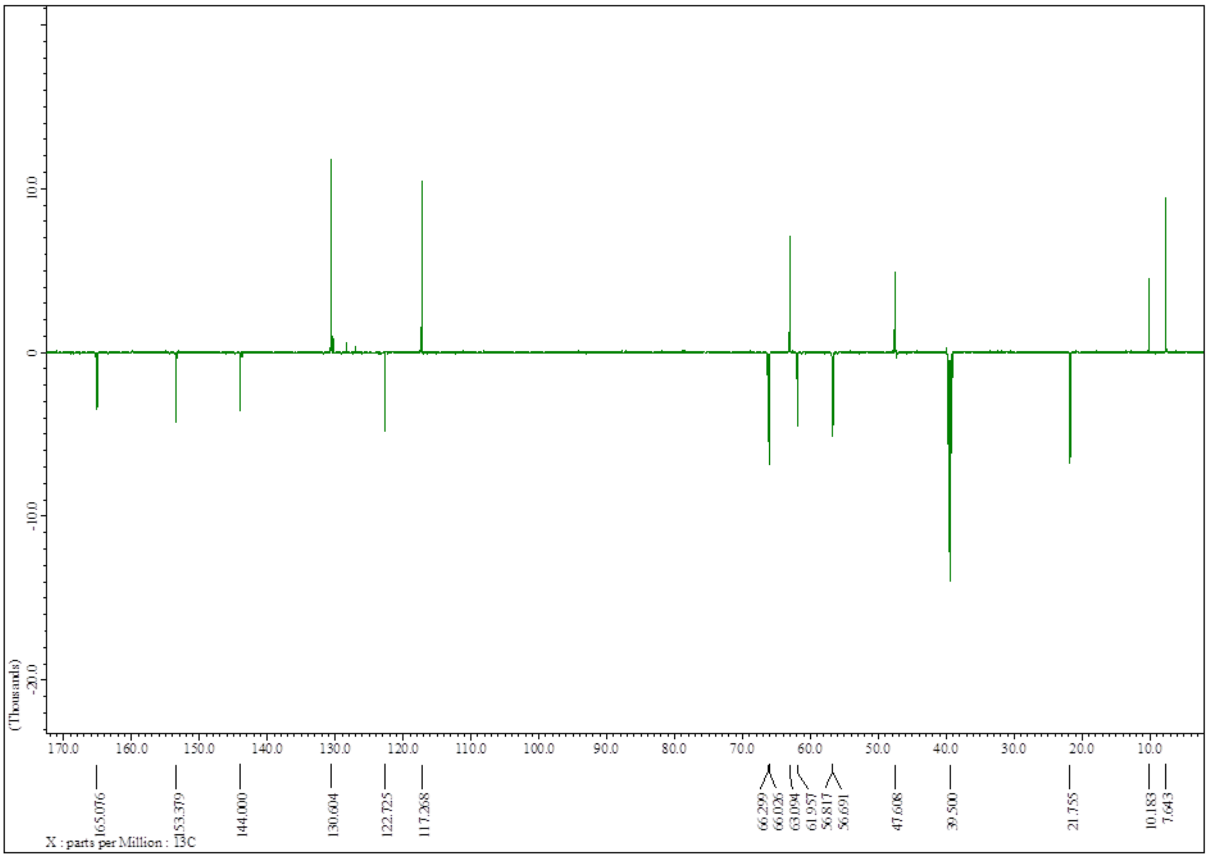

Figure S29. ^{13}C-NMR (APT) spectrum of **6h**

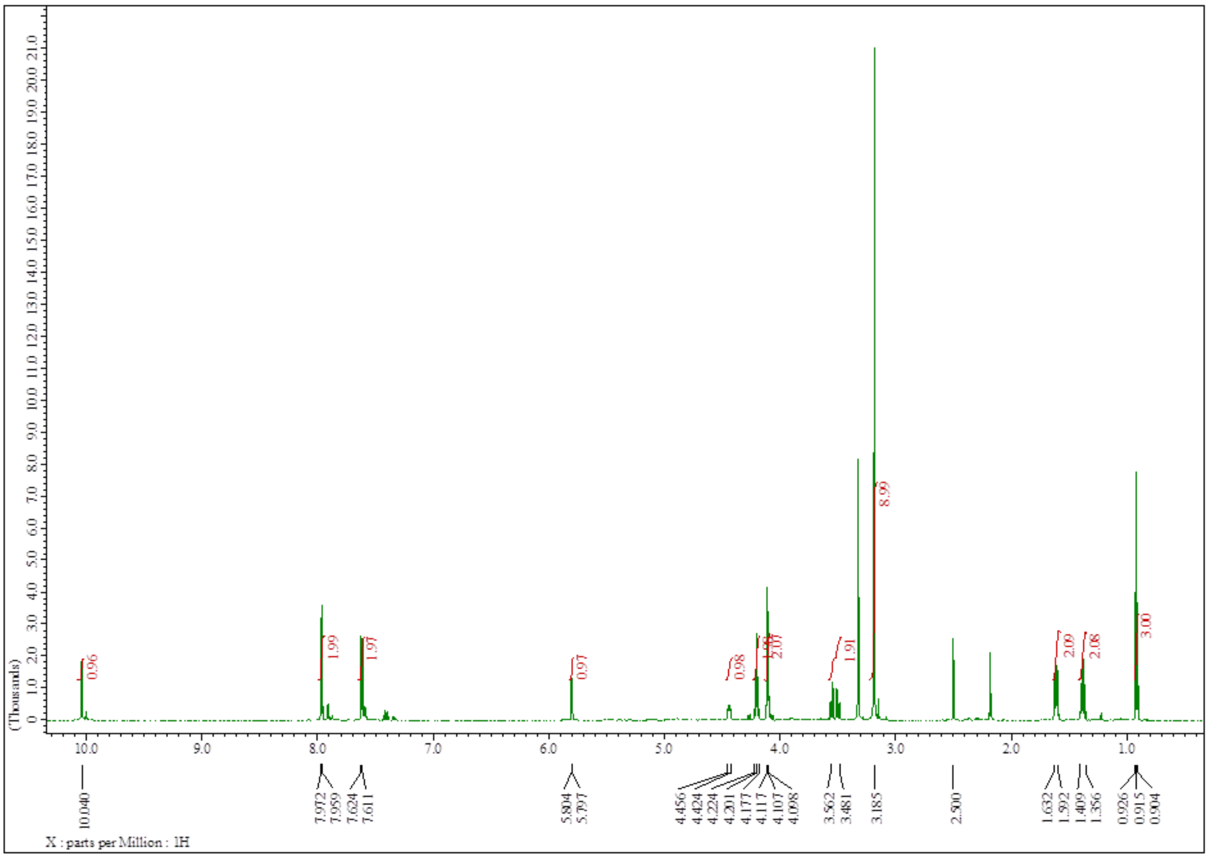

Figure S30. ^{1}H-NMR spectrum of **6i**

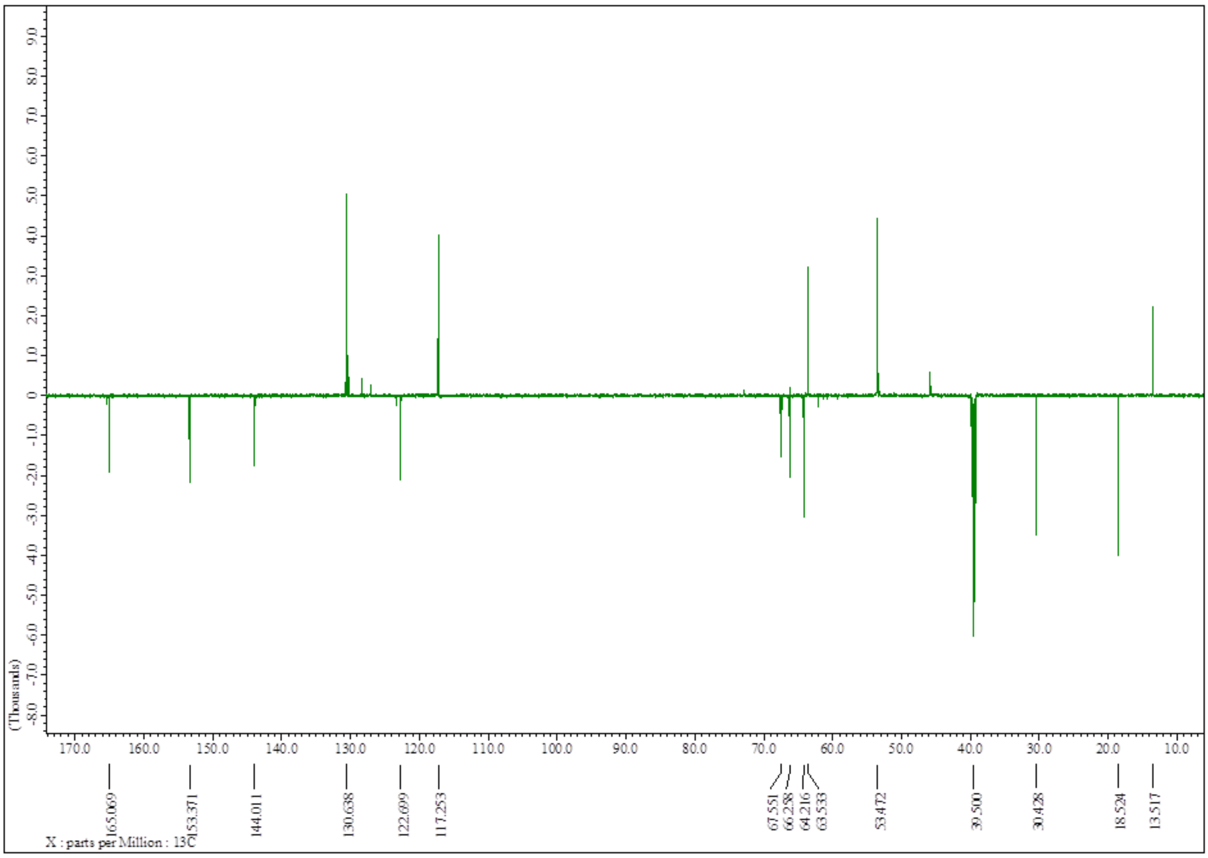

Figure S31. ^{13}C-NMR (APT) spectrum of **6i**
